# Supplementary material for: Occurrence and nature of questionable research practices in the reporting of messages and conclusions in international scientific Health Services Research publications: a structured assessment of publications authored by researchers in the Netherlands
Source: BMJ Open. 2019 May 15;9(5):e027903. doi: 10.1136/bmjopen-2018-027903 (PMC6530378; doi:10.1136/bmjopen-2018-027903)
Supplement: Supplementary file 1 [file bmjopen-2018-027903supp001.pdf]

Manuscript assessment & data extraction form (DEF)

Item

1 Assessor

1.1	Name	
1.2	Assessor role	
1.3	Assessor code	

2 General information

2.1	Title of the study	
2.2	Journal	
2.3	Number of authors	
2.4	HSR (main) domain	
2.5	Involved institutions	
2.6	Funder(s) of the study	
2.7	Role of funder in the study	
2.8	Contribution of authors is stated	
2.9	Competing interests	
2.10	EQUATOR checklist available in additional materials	
2.11	Trial registration/protocol published	

**3**	**Introduction**		**Specify**	**Evaluation/comments**
3.1	The objective(s) of the study are reported in the introduction			
3.2	The research question(s) are reported in the introduction			
3.3	The context of the study is explained			

**4**	**Methods**		**Specify**	**Evaluation/comments**
4.1	Methodological approach			
4.2	Type of research			
4.3	Research design			
4.4	Data source is reported			
4.5	Selection of participants/sample is reported			
4.6	Non-response is reported			
4.7	Size of the study is reported			
4.8	Main outcome measure(s) are reported			
4.9	Secondary outcome measure(s) are reported			
4.10	Independent variable(s) are reported			

4.11	Description of quantitative and/or qualitative methods of analyses is reported			
4.12	Handling of missing data is reported			
4.13	Comparator is explained			

**5**	**Results**		**Specify**	**Evaluation/comments**
5.1	Tables properly represent results			
5.2	Graphs properly represent results			
5.3	(Statistical) uncertainty is reported			

**6**	**Questionable messages and conclusions**	**QRP observed** (0=no; 1= yes; -8 not applicable; -9=not assessable)	**Evaluation/ comments** (rationale for assessment of QRP)	**Consulted project member** (X= consulted for advice concerning methods, specifics about study, etc.)
6.1	*Conclusions and key messages do not adequately reflect the objectives, design and actual findings*			
6.1.1	The title does not adequately reflect the main findings.			
6.1.2	The abstract does not adequately reflect the main findings.			
6.1.3	The conclusions in the abstract do not adequately reflect the conclusions in the main text.			
6.1.4	The objectives/research questions of the study are differently phrased in the introduction and the discussion.			
6.1.5	The outcome measure does not adequately reflect the objectives/research questions of the study.			
6.1.6	The main results discussed in the discussion paragraph do not adequately address the original objectives/research questions as posed in the introduction.			
6.1.7	The order of presenting the results in de discussion is inconsistent with the ordering of the objectives/research questions as posed in the introduction.			
6.1.8	The conclusions do not adequately reflect the objectives of the study.			

6.1.9	The conclusions do not adequately reflect the findings as presented in the results paragraph.			
6.1.10	The outcome measure used does not allow the conclusions that are stated.			
6.1.11	The conclusion/discussion distracts from main outcomes by overstating the relevance of secondary outcomes.			
6.1.12	The conclusions are not supported by the results as presented in context of the referenced literature.			
6.1.13	Recommendations do not adequately reflect the results in context of the referenced literature.			
6.1.14	Implications for policy and practice do not adequately reflect the results in the context of the referenced literature.			
6.1.15	Lack of distinction between results and discussion. The results section contains elements of discussion and interpretation beyond the scope of explaining the results.			

6.2	*Main results are not or inadequately interpreted into the context of evidence*			
6.2.1	Supporting evidence is poorly documented.			
6.2.2	Contradicting evidence is poorly documented.			
6.2.3	Evidence is used inappropriately to support the findings (i.e. the argument is not supported by the actual message of the cited evidence). Will be measured as: Evidence seems to be used selectively to support the findings, given the title of the referenced evidence.			
6.2.4	The main source of evidence to support the results is based on the same underlying data.			

6.3	*Limitations are not adequately mentioned*			
6.3.1	Sources, direction and magnitude of bias are not or poorly discussed, or just listed without further discussion.			
6.3.2	The possible impact of the limitations on the results (i.e., magnitude and direction of any potential sources of bias) is not or poorly discussed.			

6.4	*Unjustified generalisations*			
6.4.1	The sampling methodology does not allow the type of generalization provided.			
6.4.2	Generalization of findings to populations not included in the original sample is not justified.			
6.4.3	Generalization of findings to time periods not included in the original study is not justified.			
6.4.4	Generalization of findings to geographical locations not included in the original study is not justified.			
6.4.5	Generalization of findings to settings/institutions not included in the original study is not justified.			

6.5	*Unjustified causation*			
6.5.1	Causative wording is used in the hypothesis/research question, although there is no theory supporting causation.			
6.5.2	A causal relationship is claimed, although the research design is not appropriate to determine causation (methods lack control of potential confounding or systematic bias).			
6.5.3	A causal relationship is claimed although potential sources of bias and their potential impact on the findings were not discussed.			
6.5.4	A potential causal relationship claimed in the discussion paragraph is not justified.			

6.6	*Effect size*			
6.6.1	The relevance of statistically significant results with small effect size is overstated.			
6.6.2	The possible clinical relevance of statistically nonsignificant results is not addressed.			
6.6.3	Non-significant results are discussed without addressing significance			

6.7	*Inappropriate use of language*			
6.7.1	Hyperboles and exaggerating adjectives are unjustifiably used (such as: key, groundbreaking, ideal, excellent, great, brilliant,			

	extraordinary, impressive, completely, absolutely, entirely, everywhere, everything, nothing, beyond any doubt, definitely).			
6.7.2	Jargon, technical and complex language, that does not fit the journal audience, are used without properly explaining the meaning.			

7 Miscellaneous

7.1	Overall qualitative evaluation of the study (e.g. quality, reporting style).		
7.2	Other comments.		

8 Advice needed from second assessor

8.1	About the contents of the article		
8.2	Second assessment recommended		

Instructions per item

1 Assessor

1.1	Name	
1.2	Assessor role	
1.3	Assessor code	

2 General information

		**Instructions**
2.1	Title of the study	
2.2	Journal	
2.3	Number of authors	
2.4	HSR (main) domain	*Choose main discipline from list, add other disciplines in entry field*
2.5	Involved institutions	*List all-in*
2.6	Funder(s) of the study	
2.7	Role of funder in the study	*Copy funder declaration*
2.8	Contribution of authors is stated	
2.9	Competing interests	*Copy competing interest declaration*
2.10	EQUATOR checklist available in additional materials	
2.11	Trial registration/protocol published	*As mentioned in the article*

3 Introduction

3.1	The objective(s) of the study are reported in the introduction	
3.2	The research question(s) are reported in the introduction	
3.3	The context of the study is explained	

4 Methods

4.1	Methodological approach	
4.2	Type of research	
4.3	Research design	
4.4	Data source is reported	*e.g. registration, scientific or grey literature, survey data, interview data*
4.5	Selection of participants/sample is reported	*Selection of study enrolees also included case studies*
4.6	Non-response is reported	
4.7	Size of the study is reported	
4.8	Main outcome measure(s) are reported	

4.9	Secondary outcome measure(s) are reported	
4.10	Independent variable(s) are reported	
4.11	Description of quantitative and/or qualitative methods of analyses is reported	
4.12	Handling of missing data is reported	
4.13	Comparator is explained	

**5**	**Results**	
5.1	Tables properly represent results	*Tables give a reflection of actual results instead of cherry picking*
5.2	Graphs properly represent results	*Scaling is appropriate*
5.3	(Statistical) uncertainty is reported	*Confidence intervals are provided for the main results*

**6**	**Questionable messages and conclusions**	**Instructions**
6.1	*Conclusions and key messages do not adequately reflect the objectives, design and actual findings*	
6.1.1	The title does not adequately reflect the main findings.	*Title includes a quote or statement that does not accurately reflect/refers to the main findings, or deviates from the findings.*
6.1.2	The abstract does not adequately reflect the main findings.	*The abstracts contents deviate from / contradict with the main findings in the article text. Messy writing is not considered a QRP. Specifically for the conclusion in the abstract, causative wording misses: the conclusion in the abstract suggests causation, although the conclusions as discussed in the discussion paragraph report correlation. For instance, it is an unbalanced representation of the main results by focussing on secondary findings, while reducing the importance of the main findings, or reflects cherry-picking from the most conspicuous results. Or the stated results in the abstract in qualitative studies do not appear in the main text.*
6.1.3	The conclusions in the abstract do not adequately reflect the conclusions in the main text.	*The conclusions in the abstract are short-sighted compared to the actual conclusions in the main text. Conclusions can be stated in the discussion paragraph and/or the conclusion paragraph.*

6.1.4	The objectives/research questions of the study are differently phrased in the introduction and the discussion.	*When reporting objectives/research questions in the discussion. Different wording: does not need to include the exact wording, however the meaning/connotation should be similar. Different ordering of objectives/research questions.*
6.1.5	The outcome measure does not adequately reflect the objectives/research questions of the study.	*The objectives /research questions cannot be answered with the outcome measure that is studied*
6.1.6	The main results discussed in the discussion paragraph do not adequately address the original objectives/research questions as posed in the introduction.	*The research questions and/or objectives that were stated in the introduction section are not or only partly answered by the main results*
6.1.7	The order of presenting the results in de discussion is inconsistent with the ordering of the objectives/research questions as posed in the introduction.	*Not an actual QRP, but it does conflict with transparency in presenting the study's findings. If there's just one objective/research question, this item is not applicable (no structuring possible) and should be scored -8.*
6.1.8	The conclusions do not adequately reflect the objectives of the study.	*The objectives of the study are not met by the conclusions the study arrives at. Conclusions can be stated in the discussion paragraph and/or the conclusion paragraph. Either the study along the way shifted perspective, however no justification is provided. Or the write-up of the conclusions is flawed. Framing conclusion as extension to the discussion is not a QRP (undesirable, however beyond the scope if this indicator).*
6.1.9	The conclusions do not adequately reflect the findings as presented in the results paragraph.	*The conclusions deviate from the the main findings. Conclusions can be stated in the discussion paragraph and/or the conclusion paragraph. The conclusion section does often not contain actual conclusions. The actual conclusion is often presented in the discussion section. Hence, conclusions in the discussion section are considered conclusions as well. Concluding statements will be marked, those statements that are only used to frame results (emphasizing importance of the study) are not considered conclusions. Key messages (in a box as seperate section in some journals) are also considered conclusions.*<br>*For instance, it is an unbalanced representation of the main results by focussing on secondary findings, while reducing the importance of the main findings, or reflects cherry-picking from the most conspicuous*

		results. *If new results are presented in the discussion section, then this is a QRP. (Assessors should not recalculate results)*
6.1.10	The outcome measure used does not allow the conclusions that are stated.	*For instance: the conclusions are about the quality of the health care system, whereas the outcome measure was 'satisfaction with home-care for elderly'*
6.1.11	The conclusion/discussion distracts from main outcomes by overstating the relevance of secondary outcomes.	*The main outcomes are ignored or their importance reduced, while favouring secondary outcomes. Most space is taken by discussing these secondary outcomes.*
6.1.12	The conclusions are not supported by the results as presented in context of the referenced literature.	*If the conclusion is not based on the results, but only on referenced literature, then this is noted as QRP (as aligns with 6.1.9). The extent of the conclusions is broader/more far fetching than the findings of the study, backed-up by discussed literature, justify. Conclusions can be stated in the discussion paragraph and/or the conclusion paragraph. For instance, a relationship between IV and DV is exaggerated. Conclusions cannot be stated based on referenced literature alone, main results are the fundament for the conclusions, that may be extended based on referenced literature.*
6.1.13	Recommendations do not adequately reflect the results in context of the referenced literature.	*Recommendations: what can/should be done with the studies findings? Recommendations are based on the results from the study, not only on the referenced literature. The extent of the recommendations is broader/more far fetching than the findings of the study, backed-up by discussed literature, justify. For instance, a relationship between IV and DV is exaggerated. QRP if no justification for the suggested recommendation is provided. QRP if no recommendation is provided.*
6.1.14	Implications for policy and practice do not adequately reflect the results in the context of the referenced literature.	*Implications: what are the consequences for policy and practice if the recommendations are followed-up? What would happen if the recommendations are carried out. (e.g. recommendations = implement the intervention in this setting, implication = the outcomes may improve by this much.) QRP if no justification for suggested implication is provided, QRP if no implication is provided. Originally: implications for*

		policy and practice are poorly mentioned. Instruction: implications for practise and policy are well-balanced and give actual meaning to the findings of the study in context of practice and/or policy.
6.1.15	Lack of distinction between results and discussion. The results section contains elements of discussion and interpretation beyond the scope of explaining the results.	*Applicable to all designs. Pilot included qualitative study, but also applies to quantitative studies. Results are placed in the context of literature beyond the theoretical model of the study.*

6.2	*Main results are not or inadequately interpreted into the context of evidence*	
6.2.1	Supporting evidence is poorly documented.	*Only limited evidence to support the main results is provided and only superficially discussed. No thorough reflection of the findings in perspective of supporting evidence.*
6.2.2	Contradicting evidence is poorly documented.	*Only limited evidence to oppose against the main results is provided and only superficially discussed. No thorough reflection of the findings in perspective of contradicting evidence.*
6.2.3	Evidence is used inappropriately to support the findings (i.e. the argument is not supported by the actual message of the cited evidence). Will be measured as: Evidence seems to be used selectively to support the findings, given the title of the referenced evidence.	*State inappropriately cited references, and explain why inappropriate: the evidence ascribed to the reference deviates from what could be assumed based on the title of the reference. Includes supporting results through self-citation (without further explanation of self-citation). Self-citation is not a QRP if clearly stated "in an earlier study we found..." If no references are used to support the results (QRP 6.2.1/2), then this is no QRP (QRP is avoided by not using literature), thus assessment is not possible ans should be scored -9.*
6.2.4	The main source of evidence to support the results is based on the same underlying data.	*Most supporting evidence is grounded in the same data source as was used for the reviewed study (not necessarely self-citing), inducing circularity in argumentation.*

6.3	*Limitations are not adequately mentioned*	
6.3.1	Sources, direction and magnitude of bias are not or poorly discussed, or just listed without further discussion.	*Are the (relevant) limitations mentioned? The implications of the study design, methodology, sampling, context, etc. for risk of biasing study findings are not thoroughly discussed.*

6.3.2	The possible impact of the limitations on the results (i.e., magnitude and direction of any potential sources of bias) is not or poorly discussed.	*Is the impact of limitations discussed (if no limitations are mentioned then this is considered a QRP). The extent to which potential risks of bias affect the interpretation of the findings is not thoroughly discussed.*

6.4	*Unjustified generalisations*	
6.4.1	The sampling methodology does not allow the type of generalization provided.	*The sample is too specific, small, or flawed (for instance by attrition, selection bias) for the generalization that is made.*
6.4.2	Generalization of findings to populations not included in the original sample is not justified.	*The included sample is too specific, small or flawed (for instance by attrition, selection bias) and no or inadequate evidence is provided to support the generalization that is made. Population does not include geographical location (this is a separate QRP). Population includes population characteristics such as gender, ethnicity, age, etc.*
6.4.3	Generalization of findings to time periods not included in the original study is not justified.	*The characteristics of the included time period are too specific (for instance in election period, affecting the policy that was studied) and no or inadequate evidence is provided to support the generalization that is made*
6.4.4	Generalization of findings to geographical locations not included in the original study is not justified.	*The characteristics of the included igeographical location(s) are too specific to generalise to other geographical locations (for instance very urbanised area to rural setting) and no or inadequate evidence is provided to support the generalization that is made*
6.4.5	Generalization of findings to settings/institutions not included in the original study is not justified.	*The characteristics of the included institutions are too specific to generalise to other institutions (for instance hospital regulations to nursing homes) and no or inadequate evidence is provided to support the generalization that is made*

6.5	*Unjustified causation*	
6.5.1	Causative wording is used in the hypothesis/research question, although there is no theory supporting causation.	*Quantitative: hypothesis is not justified/allowed since there's no theory to support a causal relationship*
6.5.2	A causal relationship is claimed, although the research design is not appropriate to determine causation	*No causation based on the results of the present study may be assumed if no RCT is conducted… (or longitudinal cohort?)*

	(methods lack control of potential confounding or systematic bias).	
6.5.3	A causal relationship is claimed although potential sources of bias and their potential impact on the findings were not discussed.	*No or inadequate discussion is included concerning the impact of potential sources of bias on the possible causation that was found in the results*
6.5.4	A potential causal relationship claimed in the discussion paragraph is not justified.	*When a causal relation may not be assumed solely based on the study's findings, no or inadequate supporting and contradicting evidence is used to discuss the possible causation that was found in the results.*

6.6	*Effect size*	
6.6.1	The relevance of statistically significant results with small effect size is overstated.	*Importance of findings is exaggerated. Although (some) results are statistically significant, the clinical/practical relevance is minor due to small effect size/causation is unlikely.*
6.6.2	The possible clinical relevance of statistically nonsignificant results is not addressed.	*Importance of findings is dismissed, since no statistical significance was reached. Although the findings reflect likely causation and non-significance was likely due to lack of power.*
6.6.3	Non-significant results are discussed without addressing significance	*Results are discussed as if they were significant, without addressing they are not, or what the uncertainty is.*

6.7	*Inappropriate use of language*	
6.7.1	Hyperboles and exaggerating adjectives are unjustifiably used (such as: key, groundbreaking, ideal, excellent, great, brilliant, extraordinary, impressive, completely, absolutely, entirely, everywhere, everything, nothing, beyond any doubt, definitely).	*The use of adjectives that exaggerate the relevance of the findings, conclusions and messages. Not actually counting adjectives, if one hyperbole is used and attracted the attention. Hyperbolic adjective use per se is no QRP, only in relation to results/conclusions, to exaggerate the study's findings.*
6.7.2	Jargon, technical and complex language, that does not fit the journal audience, are used without properly explaining the meaning.	*The journal audience is not properly addressed by the language used. Language use seems to be overly complex to impress or distract the reader.*

7 Miscellaneous

7.1	Overall qualitative evaluation of the study (e.g. quality, reporting style).	*If a certain aspect impacts the answer to multiple questions, specify in "other comments". E.g. if the discussion section does not contain main results, then this item cannot be assessed.*

7.2	Other comments.	

8 Advice needed from second assessor

8.1	About the contents of the article	*What advice is needed, state question.*
8.2	Second assessment recommended	*First assessor doubts about assessment and requests second opinion.*
