# Supplementary material for: Occurrence and nature of questionable research practices in the reporting of messages and conclusions in international scientific Health Services Research publications: a structured assessment of publications authored by researchers in the Netherlands
Source: BMJ Open. 2019 May 15;9(5):e027903. doi: 10.1136/bmjopen-2018-027903 (PMC6530378; doi:10.1136/bmjopen-2018-027903)
Supplement: Supplementary file 2 [file bmjopen-2018-027903supp002.pdf]

February 5th, 2019

Supplement to the methods section of:

The occurrence and nature of questionable research practices in the reporting of messages and conclusions in international scientific Health Services Research publications: A structured assessment of publications authored by researchers in the Netherlands

Additional information to the methods of the development of the definition and measurement instrument for "questionable research practices in the reporting of messages and conclusions in scientific health services research publications"

This document describes the methods used to develop a definition of questionable research practices (QRPs) in the reporting of messages and conclusions, and to construct a measurement instrument that allows for the identification of questionable research practices in the reporting of messages and conclusions in Health Services Research (HSR).

Methodology
Methods included an explorative review of definitions in literature, a consultation meeting with the project group, institution/department leaders of Dutch HSR institutions and project advisors (n=13), semi-structured interviews with 13 HSR institutes (n=19) and an expert consultation (n=5).

Setting
13 HSR groups, departments, or institutions (hereafter referred to as "HSR institutions") in the Netherlands, including both academic and non-academic institutions participated in this study. These institutions all agreed to participate in an effort to assure the overall quality of HSR publications in the Netherlands.

Literature review
First, a literature review was conducted searching for existing definitions of questionable research practices in the reporting of conclusions and messages, and operationalisations of QRPs. Search terms included in different order and combination: 'questionable research practices', 'spin', 'over interpretation', 'discordant conclusions', 'QRPs', 'outcome reporting bias', 'questionable conclusions' and 'responsible conclusions'. Documents were included if they described methods to measure questionable research practices in scientific publication, or provided definitions of the above key terms. Referred documents that fit the criteria were also included in the review.

After identifying the main literature that suited our aim, we came to a preliminary definition of QRPs based on Boutron 2010, Ochobo 2013, and Horton 1995 [1-3].

An extensive list of possible types of QRPs in the reporting of messages and conclusions was developed, based on the EQUATOR checklists[4] and instruments from previous studies. For example, instruments for identifying 'spin', reporting of qualitative research and other QRPs such as [3,5-7]. Spin in this context refers to "a way to distort science reporting without actually lying")

Consultation meeting
Second, we presented the preliminary QRP definition and the first draft of items referring to QRPs (see page 3) during a consultation meeting of participating HSR institutions on 6 June 2017. The meeting lasted three hours, during which the research project and the preliminary definition and draft of QRP items was discussed. Representatives of the participating HSR institutions (n=7), project advisors (n=2) and project group members (n=4) attended the meeting. The attendees discussed their thoughts about the definition and its operationalisation. Detailed notes from this meeting were summarized and shared with the representatives of all participating institutes (including those who did not attend).

The central conclusion of the meeting was to focus on the 'measurability' of the QRPs. An important consideration in developing the instrument for the assessment of scientific publication is to focus on the possibility to measure the QRPs. Therefore, the focus should be on QRPs that can be quantified. These should be distinguished from QRPs that, although possibly important, are not quantifiable.

Semi-structured interviews
Third, we conducted fourteen semi-structured interviews with nineteen leaders/representatives of the thirteen HSR institutions. These representatives had to have a clear overview of the process of reporting research in their institute. One of the institutions was represented by two separate departments, hence two representatives were separately interviewed. Three interviews were conducted with both the institute leader and a second representative. One of the interviews included three representatives of an institution. The aim of the interviews was to discuss our draft of QRP items and identify additional measurable QRPs in the reporting of messages and conclusions in HSR, explore potential causes of QRPs in messages and conclusions, and to discuss experiences of the institute leaders with these QRPs. A semi-structured interview guide was developed by the project team (see page 4-5). During the interview, we presented the interviewees with a draft of QRP items. The draft list was iteratively adjusted, i.e. after each interview we drafted a new version including the findings of the previous interviews.

Interviewees were approached through e-mail to schedule an appointment. Two researchers conducted the interviews of which thirteen took place at the participating institutions and one interview took place in a public space. During the first interview, both researchers were present to align the approach. The remainder of the interviews were equally divided between them. The interviews lasted one hour. In concordance with ethical guidelines, the goal of the interview was explained at the start of the interview and permission to audio-record the interview was obtained.

With the support of the recordings, a report was written and shared with the interviewees for validation. All interviewees confirmed the reports, after mostly minor edits to the report. From the interview reports, we drew up a new draft of the list of QRP items (see page 6-7). In the research group, we specifically paid attention to correct wording of the QRPs.

Expert consultation
Fourth, ten leading international health services researchers were asked to provide feedback on this list of QRP items. These HSR experts were invited through e-mail in which we explained the aim of the study, and included the definition of QRPs and the list of QRP items. Five experts provided their comments to the items. Five experts did not respond after a reminder, or indicated not having time to review the QRP items. Feedback was summarized, and comments were used to adapt the QRP definition and list of QRP items.

Measurement instrument
We developed the measurement instrument in Excel format by taking items from earlier developed checklists (EQUATOR and COREQ) and the list of QRPs. The measurement instrument was completed after a final consensus meeting of the research group. The measurement instrument exists of three sections: 1) bibliographic information of the publication (eg. funder, journal, number of authors), 2) basic methodological information (eg. included population, analyses method) and 3) possible QRPs in messages and conclusions. A pilot was conducted to assess the feasibility and usability of the instrument. In the pilot, two project members independently assessed five international HSR publications to identify modifications needed to improve the items in the instrument, and to align the interpretation of the items. The project group discussed the proposed modifications, resulting in the final version: the data extraction form (see supplementary material 1.)

List of possible questionable research practices presented during the consultation meeting and the interviews

With each interview, new QRP's were added to the list which were then presented during the next interview.

Definition: Questionable reporting of messages and conclusions:

> *"The use of reporting, from whatever motive, consciously or unconsciously, to make conclusions or messages weaker or stronger than results justify."*

Potential

1. Poorly set results into context of totality of evidence
2. No mention of contradictory evidence
3. Discrepancy between the aim of the study and the conclusion
4. No reporting objectives, aim or research question
5. Concealing limitations
6. Lack of transparency of methods used
7. *Not reporting a hypothesis*
8. Selective reporting of results in conclusion
9. No mention of [statistical] uncertainty

Actual

1. Discrepancy between the title, abstract and the article
2. Describing unjustified causation
3. Inappropriate citing
4. Authorial rhetoric
5. Misleading graphs and tables
6. Unjustified generalisations (mismatch between study population, sex, geographical entities and time period)
7. *Stating the [intervention/measure] is beneficial despite statistically nonsignificant difference for the primary outcome*
8. *Distract the reader from statistically nonsignificant results*
9. Not explaining the comparator/context of the intervention

Interview guide used during the semi-structured interviews (in Dutch)

Interviewprotocol eerste consultatieronde juni/juli 2017

Toelichting op het interview

Het doel van het ZonMw Project is om te komen tot aanbevelingen ter bevordering van verantwoord rapporteren over gezondheidszorgonderzoek (responsible conclusions and messages in health services research).

Hoewel de projectleiding primair bij het AMC ligt, is het binnen het project nadrukkelijk een gedeelde verantwoordelijkheid van alle dertien betrokken instituten om te komen tot voorstellen ter bevordering van verantwoord rapporteren van gezondheidszorgonderzoek. We houden in deze eerste fase interviews met de hoofden en vertegenwoordigers van de betrokken HSR instituten. Tijdens het interview worden de volgende onderwerpen besproken:

1) Potentiele oorzaken van QRPs in het vormen van conclusies en berichten
2) Het meten van QRP in conclusies en berichten in HSR
3) Uw ervaring met Responsible en Questionable Research Practices.

Het interview zal 1 uur in beslag nemen. Indien u daarvoor toestemming geeft, zal het gesprek worden opgenomen, en notities van het gesprek zullen worden uitgewerkt. Het gespreksverslag zal vervolgens ter verificatie aan u worden voorgelegd. Het gesprek wordt vertrouwelijk behandeld; alleen de onderzoekers op dit project zullen inzicht hebben in de inhoud van dit gesprek. De rapportage van de bevindingen zal op geaggregeerd niveau plaatsvinden. Uitspraken zullen daarbij niet-herleidbaar tot persoon en/of instituut worden gerapporteerd.

Wij zullen eerst onze bevindingen tot nu toe kort toelichten, en vervolgens verdergaan met het interview.

1) Bevindingen startbijenkomst
    a. Positieve start
    b. Nadruk op betrokkenheid alle instituten (veel feedbackloops)

Vragen

Probleem onderkenning

1. Acht U het zinvol om naar ongeoorloofd rapporteren (QRP) bij gezondheidszorg onderzoek in NL te kijken?
    a. Indien ja, waarom denkt U dat het een reëel probleem is
    In de omvang, neemt het toe of af?

Beleid & structuur (wat doe u in uw rol als instituutshoofd om dit probleem aan te pakken)

2. Kunt u iets vertellen over de wijze waarop er binnen uw instituut wordt omgegaan met het stimuleren van verantwoord rapporteren van (HSR) onderzoek?
    a. Is er specifiek beleid op het verantwoord rapporteren van HSR?
    Indien ja, kunt u dat toelichten? (Open doorvragen, voorbeelden)

    b. Is er specifiek beleid, procedures, werkwijzen om QRPs in rapporteren van HSR te voorkomen?
    Indien ja, kunt u dat toelichten? (Open doorvragen, voorbeelden)

Definitie QRPs

Tot nu toe hebben wij de volgende QRPs geïdentificeerd in het rapporteren van onderzoek in wetenschappelijke publicaties [lijst QRPs].

3. Bent u het eens met (de formulering van) deze QRPs en heeft u opmerkingen en aanvullingen op deze lijst?
    a. Wat wilt u veranderen en of toevoegen?
4. Wij willen een keuze maken uit specifieke, goed te meten QRPs in het rapporteren van conclusies en berichten in HSR. Welke QRPs voldoen naar uw mening aan deze criteria?

Ervaringen

5. Wat zijn uw ervaringen met het rapporteren van resultaten van gezondheidszorgonderzoek?
    a. Wat gaat naar uw ervaring goed?
    b. Wat zijn in uw ervaringen knelpunten? Kunt u voorbeelden noemen waarin deze knelpunten naar voren kwamen?

Toelichting framework

Op dit moment hebben wij de factoren van invloed QRP als volgt weergeven [framework].

6. Zijn dit volgens u juiste factoren?
7. Wat zijn naar uw mening (nog meer) belangrijke factoren van invloed op RRPs en QRPs?
8. Welke factoren zou u als eerste aanpakken? Waarom deze factoren? Wat verwacht u daarvan?

Afsluiting

9. Heeft U nog aanvullende suggesties hoe in samenwerking met de andere gezondheidszorgonderzoeksinstituten in NL kan worden bijgedragen aan verantwoord rapporteren?

February 5th, 2019

QRP list and comment form used for expert consultation

Experts provided comments in the comment boxes

Questionable reporting of conclusions and messages in Health Services Research

Expert consultation

Definition:

"To frame, from whatever motive, consciously or unconsciously, conclusions or messages as an answer to the research question that are not justified by the results"

[Comments concerning definition]

Measuring questionable reporting of conclusions & messages

Title, abstract, main text, and conclusions do not align

1.1. The title does not align with the main text.
1.2. The abstract does not align with the main text.
1.3. The conclusions in the abstract do not align with the conclusions in the main text.
1.4. The objectives/research questions of the study are differently phrased in the introduction. and the discussion.

[Comments concerning category 1]

Conclusions do not reflect the objectives and results properly

2.1. The main results in the discussion do not follow from the research questions.
2.2. The conclusions do not align with the results in the main text.
2.3. The order of presenting the results is inconsistent with the research questions.
2.4. The conclusion/discussion distracts from main outcomes by overstating the relevance of secondary outcomes.
2.5. The relevance of statistically significant results with small effect sizes is overstated.
2.6. Possible clinical relevance of statistically insignificant results is not addressed.
2.7. The conclusions do not reflect the objectives of the study.
2.8. The conclusions are not supported by the results in context of the discussed literature.
2.9. Recommendations do not follow from the results and discussed literature.
2.10. Implications for policy and practice are poorly mentioned.

[Comments concerning category 2]

Main results are poorly put into the context of evidence

3.1. Supporting evidence is poorly mentioned.
3.2. Contradicting evidence is poorly mentioned.
3.3. Citations are used inappropriately to support the conclusions (i.e. the actual message of the cited source does not align with the conclusion it should support).
3.4. Self-citations or studies based on the same data are the main source of supporting evidence.

[Comments concerning category 3]

Limitations are not properly mentioned

4.1. Limitations are discussed only superficially (for instance only on one level, e.g. the measurement level, design, sample).
4.2. No sources of bias are mentioned.
4.3. The possible effect of the limitations on the results is not discussed.

[Comments concerning category 4]

Unjustified generalizations

5.1. The time of data collection does not align with the time for which the conclusions are presented.
5.2. The study sample does not align with the population the conclusions are generalized to.
5.3. No justifications are offered for generalizations:
  5.3.1. In time.
  5.3.2. In geographical location.
  5.3.3. To setting/institution.

[Comments concerning category 5]

Unjustified causation

6.1. Causative wording that is used that is not allowed by the study design.
6.2. A causal relationship is claimed without mentioning any theoretical explanation of the relation.

[Comments concerning category 6]

Inappropriate language use

7.1. Hyperboles and exaggerating adjectives are used without justification (such as: ideal, excellent, great, brilliant, extraordinary, impressive, completely, absolutely, entirely, everywhere, everything, nothing, beyond any doubt, definitely).
7.2. Jargon, technical and complex language are used without properly explaining the meaning.

[Comments concerning category 7]

References

1. Boutron I, Dutton S, Ravaud P, Altman DG: 'Spin' in reports of randomized controlled trials with nonstatistically significant primary outcomes. 6th International Congress on Peer Review and Biomedical Publication, Vancouver. (Accessed 30 March 2010), [http://www.ama-assn.org/public/peer/abstracts_2009.html#30].
2. Horton R. The rhetoric of research. *BMJ* 1995; **310**(6985): 985.
3. Ochodo EA, de Haan MC, Reitsma JB, Hooft L, Bossuyt PM, Leeflang MM. Overinterpretation and misreporting of diagnostic accuracy studies: evidence of "spin". *Radiology* 2013; **267**(2): 581-8.
4. Altman DG, Simera I, Hoey J, Moher D, Schulz K. EQUATOR: reporting guidelines for health research. *Lancet* 2008; **371**.
5. Stevens GA, Alkema L, Black RE, et al. Guidelines for Accurate and Transparent Health Estimates Reporting: the GATHER statement. *PLoS Med* 2016; **13**(6): e1002056.
6. Tong A, Sainsbury P, Craig J. Consolidated criteria for reporting qualitative research (COREQ): a 32-item checklist for interviews and focus groups. *Int J Qual Health Care* 2007; **19**(6): 349-57.
7. Boutron I, Dutton S, Ravaud P, Altman DG. REporting and interpretation of randomized controlled trials with statistically nonsignificant results for primary outcomes. *JAMA* 2010; **303**(20): 2058-64.
