# Supplementary material for: Occurrence and nature of questionable research practices in the reporting of messages and conclusions in international scientific Health Services Research publications: a structured assessment of publications authored by researchers in the Netherlands
Source: BMJ Open. 2019 May 15;9(5):e027903. doi: 10.1136/bmjopen-2018-027903 (PMC6530378; doi:10.1136/bmjopen-2018-027903)
Supplement: Supplementary file 3 [file bmjopen-2018-027903supp003.pdf]

Supplementary material 3

Table 1. Occurrence of QRPs in the reporting of messages and conclusions in HSR publications (n=116), ordered from most frequently to least frequently occurring (%).

**Questionable research practices (QRPs) in reporting messages and conclusions**	**% publications with QRP**	**% publications without QRP**	**% publications for which QRP not assessable**
Implications for policy and practice do not adequately reflect the results in the context of the referenced literature.	**69·0	31·1	0·0
Recommendations do not adequately reflect the results in the context of the referenced literature.	***65·5	34·5	0·0
Contradicting evidence is poorly documented.	63·8	36·2	0·0
Conclusions do not adequately reflect the findings as presented in the results section.	46·6	51·7	1·7
Possible impact of the limitations on the results is not or poorly discussed.	44·0	56·0	0·0
Conclusions are not supported by the results as presented in the context of the referenced literature.	43·1	54·3	2·6
The conclusions do not adequately reflect the objectives of the study.	35·3	61·2	3·4
Supporting evidence is poorly documented.	31·9	68·1	0·0
Sources. direction and magnitude of bias are not or poorly discussed. or just listed without further discussion.	27·6	72·4	0·0
The conclusions in the abstract do not adequately reflect the conclusions in the main text.	22·4	75·0	2·6
The main results discussed in the discussion paragraph do not adequately address the original objectives/research questions as posed in the introduction.	20·7	75·9	3·4
The outcome measure used does not allow the conclusions that are stated. *	18·1	81·9	0·0
Lack of distinction between results and discussion. The results section contains elements of discussion and interpretation beyond the scope of explaining the results.	17·2	82·8	0·0
The sampling methodology does not allow the type of generalization provided.	15·5	84·5	0·0
The objectives/research questions of the study are differently phrased in the introduction and the discussion.	14·7	36·2	49·1
The order of presenting the results in de discussion is inconsistent with the ordering of the objectives/research questions as posed in the introduction.	14·7	75·0	10·3
Hyperboles and exaggerating adjectives are unjustifiably used	12·1	87·9	0·0
The title does not adequately reflect the main findings.	11·2	88·8	0·0
The abstract does not adequately reflect the main findings.	10·3	89·7	0·0
A potential causal relationship claimed in the discussion paragraph is not justified.	10·3	89·7	0·0
The outcome measure does not adequately reflect the objectives/research questions of the study. *	9·6	90·4	0·0
A causal relationship is claimed. although the research design is not appropriate to determine causation.	9·6	90·4	0·0
The relevance of statistically significant results with small effect size is overstated. *	9·6	90·4	0·0
Generalising findings to settings/institutions not included in the original study is not justified.	9·5	89·7	1·0
The conclusion/discussion distracts from main outcomes by overstating the relevance of secondary outcomes. *	8·4	91·6	0·0

Non-significant results are discussed without addressing significance.	8·4	91·6	0·0
Generalising findings to geographical locations not included in the original study is not justified.	6·0	94·0	0·0
Evidence is used inappropriately to support the findings.	5·2	94·9	0·0
A causal relationship is claimed although potential sources of bias and their potential impact on the findings were not discussed. *	3·6	96·4	0·0
Jargon. technical and complex language. that does not fit the journal audience. are used without properly explaining the meaning.	3·4	96·6	0·0
The main source of evidence for supporting the results is based on the same underlying data.	2·6	96·6	0·9
Generalising findings to populations not included in the original sample is not justified.	2·6	97·4	0·0
Causative wording is used in the hypothesis/research question, although there is no theory to support causation. *	2·4	97·6	0·0
Possible clinical relevance of statistically non-significant results is not addressed. *	2·4	97·6	0·0
Generalising findings to time periods not included in the original study is not justified.	0·0	100·0	0·0

** QRPs only applicable to quantitative research-based publications (n=83)*
*** 50 ·0% of publications did not mention implications for policy or practice.*
**** 34·5% of publications did not mention recommendations for policy or practice.*
