# Supplementary material for: Occurrence and nature of questionable research practices in the reporting of messages and conclusions in international scientific Health Services Research publications: a structured assessment of publications authored by researchers in the Netherlands
Source: BMJ Open. 2019 May 15;9(5):e027903. doi: 10.1136/bmjopen-2018-027903 (PMC6530378; doi:10.1136/bmjopen-2018-027903)
Supplement: Author's manuscript [file bmjopen-2018-027903.draft_revisions.pdf]

BMJ Open is committed to open peer review. As part of this commitment we make the peer review history of every article we publish publicly available.

When an article is published we post the peer reviewers' comments and the authors' responses online. We also post the versions of the paper that were used during peer review. These are the versions that the peer review comments apply to.

The versions of the paper that follow are the versions that were submitted during the peer review process. They are not the versions of record or the final published versions. They should not be cited or distributed as the published version of this manuscript.

BMJ Open is an open access journal and the full, final, typeset and author-corrected version of record of the manuscript is available on our site with no access controls, subscription charges or pay-per-view fees (http://bmjopen.bmj.com).

If you have any questions on BMJ Open's open peer review process please email editorial.bmjopen@bmj.com

BMJ Open

Towards responsible messages and conclusions in scientific Health Services Research publications: Exploring questionable research practices

Journal:	*BMJ Open*
Manuscript ID	bmjopen-2018-027903
Article Type:	Research
Date Submitted by the Author:	13-Nov-2018
Complete List of Authors:	Gerrits, Reinie; Amsterdam UMC, University of Amsterdam, Amsterdam Public Health research institute, Public Health<br>Jansen, Tessa; Amsterdam UMC, University of Amsterdam, Amsterdam Public Health research institute, Public Health<br>Mulyanto, Joko; Amsterdam UMC, University of Amsterdam, Amsterdam Public Health research institute, Public Health<br>van den Berg, Michael; Amsterdam UMC, University of Amsterdam, Amsterdam Public Health research institute, Public Health<br>Klazinga, Niek; Amsterdam UMC, University of Amsterdam, Amsterdam Public Health research institute, Public Health<br>Kringos, Dionne; Amsterdam UMC, University of Amsterdam, Amsterdam Public Health research institute, Public Health
Keywords:	Health Services Research, Responsible Research Practices, Questionable Research Practices, Scientific Reporting

SCHOLARONE™
Manuscripts

Towards responsible messages and conclusions in scientific Health Services Research publications: Exploring questionable research practices

Reinie G. Gerrits1, MSc, Tessa Jansen1 MSc, Joko Mulyanto1 MD, Michael J. van den Berg1 PhD, Niek S. Klazinga1 PhD, Dionne S. Kringos1 PhD.

1 Amsterdam UMC, University of Amsterdam, Department of Public Health, Amsterdam Public Health research institute, P.O. Box 22660, 1100 DD Amsterdam, The Netherlands

Corresponding author
Reinie G. Gerrits
Address: P.O. Box 22660, 1100 DD Amsterdam, the Netherlands
Telephone: +31683555977
E-mail: r.g.gerrits@amc.uva.nl

For peer review only

For peer review only - http://bmjopen.bmj.com/site/about/guidelines.xhtml

Abstract

Objectives: Explore the occurrence and nature of questionable research practices (QRPs) in the reporting of messages and conclusions in international scientific HSR publications authored by researchers from health services research (HSR) institutions in the Netherlands.
Design: In a joint effort to assure the overall quality of HSR publications in the Netherlands, thirteen HSR institutions in the Netherlands participated in this study. Together with these institutions, we constructed and validated an assessment instrument covering 35 possible QRPs in the reporting of messages and conclusions. Two reviewers independently assessed a random sample of 116 HSR articles authored by researchers from these institutions published in international peer-reviewed scientific journals in 2016.
Setting: Netherlands, 2016.
Sample: 116 international peer-reviewed HSR publications.
Main outcome measures: Median number of QRPs per publication, the percentage of publications with observed QRP frequencies, occurrence of specific QRPs, and difference in total number of QRPs by methodological approach, type of research, and study design.
Results: We identified a median of six QRPs per publication, out of 35 possible QRPs. QRPs occurred most frequently in the reporting of implications for practice, recommendations for practice, contradictory evidence, study limitations, and conclusions based on the results and in the context of the literature. We identified no differences in total number of QRPs in papers based on different methodological approach, type of research or study design.
Conclusions Given the applied nature of HSR, both the severity of the identified QRPs, and the recommendations for policy and practice in HSR publications warrant discussion. We recommend that the HSR field further define and establish its own scientific norms in publication practices to improve scientific reporting and strengthen the impact of HSR. The results of our study can serve as an empirical basis for continuous critical reflection on the reporting of messages and conclusions.

Funding: ZonMw grant number 445001003.

Strengths and limitations of the study

- We applied a broad and sensitive definition of ‘questionable’, for instance by considering the absence of contradictory evidence or the absence of implications and recommendations for policy and practice as a QRP.

- With our broad definition encompassing 35 possible QRPs we bring to light the areas that offer possibilities for further enhancing publication practices in HSR. Consequently, this definition allows for a discussion in the field of HSR on the extent to which the identified QRPs are acceptable. This is an important strength of our applied approach.

- Although we endeavoured to develop a reliable measurement instrument that would guide the review process, the instrument allowed latitude for the reviewer’s interpretation. Consequently, a different group of reviewers might arrive at somewhat different scoring frequencies for observed QRPs.

- Our consensus method contains a degree of subjectivity, and there is the risk that one reviewer’s opinion will dominate. To counteract this, NK and DK performed random checks on 10% of all assessments. By recording the motivation for every identified QRP, we supported the consistency of our measurement and justified our results.

- Because publications were selected based on the title, selection bias might have occurred. Considering we found no relationship between study characteristics and number of QRPs, it is unlikely that a different sample would have led to different results.

For peer review only - http://bmjopen.bmj.com/site/about/guidelines.xhtml

Introduction

In 2009, Chalmers and Glasziou estimated that 85% of research funding in biomedical sciences was wasted avoidably,[1] resulting in *The Lancet*'s series "Increasing value: reducing waste". This series has stirred the international scientific community, prompting funders, regulators, academic institutions, and scientific publishers to act. Funders of biomedical research have responded by organising conferences on research waste, and journal editors have initiated discussions on data sharing and open access.[2] While evidence for questionable research practices (QRPs) in biomedical sciences is mounting,[1] little is known about the occurrence and nature of QRPs in the policy- and management-oriented field of health services research (HSR). The term 'questionable' covers a wide range of practices. A questionable practice is not necessarily wrongful, but does 'raise questions'.

The HSR field is an applied field of research, and produces evidence on topics such as co-payments, evaluation of quality improvement efforts, cost-effectiveness of medications, patient empowerment, therapy compliance, and effects of policies. Given the growing evidence for the prevalence of QRPs in the reporting of messages and conclusions in the biomedical field,[3,4] QRPs may also occur in the HSR field. Just like biomedical researchers, health services researchers are under pressure to publish in high-impact journals to increase their citation scores and attract media attention to augment their prestige and chances for future research funding and job security.[5-8] Unlike biomedical research, HSR findings are not easily generalised from one local or national health services setting to another, and messages and conclusions tend to be limited to a specific national context.[9] A broad spectrum of quantitative and qualitative methods is used in HSR, including designs that are less subject to strict codes of execution than randomized controlled trials, such as observational and case study designs. Furthermore, HSR has difficulty creating alignment between the construction of scientific knowledge and the implementation of that knowledge in policy and practice.[10]

Although reporting in scientific publications is highly standardised, the discussion and conclusion sections offer researchers relative freedom when deriving messages and conclusions from study results.[4] We explored the occurrence and nature of QRPs in the reporting of messages and conclusions in international scientific HSR publications authored by researchers from HSR institutions in the Netherlands. We also examined the relationship between study type, methodology, and design and the occurrence of QRPs. With our study, we want to fuel the debate on fostering responsible messages and conclusions, and provide a basis for the discussion on QRPs in the international HSR field.

What is already known about this topic

- In the biomedical field, estimates for the occurrence of questionable research practices (QRPs) in the interpretation of results in scientific publications vary from 10% of publications deriving discordant conclusions from study results to 100% of publications containing rhetorical practices resulting in spin.
- The debate on fostering responsible reporting practices to date mainly focusses on the biomedical field. Knowledge on the scientific reporting in the applied field of Health Services Research (HSR) is lacking.

Added value of this study

- With this explorative study, we identify a broad scope of QRPs in the reporting of messages and conclusions in HSR publications. Furthermore, we demonstrate that recommendations for policy and practice are not commonly reported in Health Services Research publications, despite the policy- and management-oriented nature of Health Services Research (HSR).
- To ensure the applicability of HSR, those in this field should reflect on the severity of the nature of identified QRPs, and the inclusion and form of recommendations for policy and practice.
- The results of our study open the debate on the current state of research, and form an empirical basis for the discussion on how to systematically advance the reporting of messages and conclusions in the field of HSR.

For peer review only - http://bmjopen.bmj.com/site/about/guidelines.xhtml

Methods

Setting

This study assessed scientific publications authored by researchers from 13 HSR groups, departments, or institutions (hereafter referred to as "HSR institutions") in the Netherlands, including both academic and non-academic institutions. These institutions all agreed to participate in an effort to assure the overall quality of HSR publications in the Netherlands.

Defining QRPs in the reporting of messages and conclusions in HSR

We conducted a literature review on QRPs in the reporting of messages and conclusions in biomedical research and HSR.[11-13] An initial definition of QRPs in the reporting of messages and conclusions in HSR was proposed and discussed at a consensus meeting with the directors/leaders of the 13 participating institutions. This was then validated through inputs from five leading international health services researchers (10 were invited; 50% non-response), and resulted in the following amended definition:

"To report, either intentionally or unintentionally, conclusions or messages that may lead to incorrect inferences and do not accurately reflect the objectives, the methodology or the results of the study."

Measurement instrument

We developed an extensive list of QRPs in the reporting of messages and conclusions. Items were based on the EQUATOR checklists[14] and earlier checklists for identifying "spin" (ie, "a way to distort science reporting without actually lying")[4] or other QRPs.[12,13,15,16] The proposed list of QRPs was reviewed, refined, and complemented using 14 semi-structured interviews with the directors/leaders and representatives (n=19) of the 13 participating HSR institutions. Next, the five participating international health services researchers provided email feedback on the list resulting from these interviews; the list was adapted accordingly, resulting in 35 possible QRPs in the reporting of messages and conclusions in HSR publications.

We developed a data extraction form (DEF) in Excel that contained the list of QRPs and bibliometric information, and conducted a pilot to evaluate its feasibility and usability. In the pilot, two assessors (RG, TJ) independently assessed five international HSR publications to identify modifications needed to improve the form, and to align the interpretation of the items. The project group discussed the proposed modifications, resulting in the final version (see supplementary material).

Sample

We aimed to include ten HSR publications from each participating HSR institution. Inclusion criteria were: published in 2016 in an international peer-reviewed scientific journal, written in English, reporting HSR findings, and first- and/or last-authored by researchers affiliated with the respective HSR institution.

Publication lists of the HSR institutions were retrieved either by searching publicly accessible online sources (eg, annual reports, open repositories or the research groups' website) or obtained from secretaries or librarians. All lists were verified by the respective HSR institutions.

Two researchers (RG, TJ) selected all titles from the 13 publication lists that were likely to indicate empirical or systematic assessment studies in HSR, using the definitions of HSR by Juttmann (2007)[17] and Lohr & Steinwachs (2002).[18] These definitions are commonly used by HSR institutions (eg, in education) in the Netherlands.

The HSR publications (n=717) were assigned a random number. Per institution, the publications with unique first authors with the lowest assigned number were included in the sample. Three HSR institutions did not have enough publications with unique first authors, resulting in a selection of nine, eight, and two publications for these institutions. Furthermore, two publications were excluded during assessment because they concerned research protocols. These publications were replaced by another publication authored by the same institution. One publication was excluded because its methodology was considered incomprehensible by the reviewers. Ultimately, 116 HSR publications were included (16% of selected publications).

Assessment process

Two reviewers independently assessed all publications (RG and TJ or RG and JM). RG has primarily qualitative HSR experience and is trained in health economics. TJ and JM have primarily quantitative HSR experience and are trained in public health, management, economics, and law; and medicine, respectively.

For peer review only - http://bmjopen.bmj.com/site/about/guidelines.xhtml

The assessment started with a test phase. During this phase, agreements and disagreements in assessments of the first 30 publications were thoroughly discussed (by RG, TJ, NK, and DK) to increase the accuracy of the assessments; agreement was 81%, which increased to 82% during the second round. The notion emerged that it was necessary having two reviewers with complementary expertise assess each publication independently, followed by a consensus procedure and random check by the project leaders. RG trained the third reviewer (JM).

RG assessed all included publications, while TJ assessed the first 59 publications, and JM the remaining 57. All data were entered in the DEF. QRPs were coded as either 1, “present”; 0, “not present”; -8, “not applicable to this study” (primarily used for items not applicable for qualitative research); or -9, “not assessable”. To justify their assessments, the reviewers recorded their motivation for every identified QRP. At a later stage, QRPs in implications and recommendations for policy and practice were further refined into “not mentioned” if no implication or recommendation was included in the publications, and “not sufficiently justified”, if the authors did not provide any explanation for their implications or recommendations. The reviewers held regular consensus meetings to discuss and reach agreement on all identified QRPs. Any remaining disagreements (n=2) were resolved by a senior researcher (DK). NK and DK reassessed a random sample of six publications, so 10% of all included publications. As a result, two identified QRPs were retracted, and two QRPs were added.

Analysis

The characteristics of the included publications were described by calculating their occurrence with the percentage or mean number of publications subject to the scale.

We counted the total number of QRPs per publication, and the percentage of HSR publications with number of observed QRPs. The latter was visualised in a histogram. Occurrence of specific QRPs was calculated as a percentage of publications containing this particular QRP. The percentage of publications containing QRPs that were not applicable to qualitative research was calculated only for quantitative and mixed-methods-based publications (n = 83).

We used a Kruskal-Wallis test to calculate the difference in total number of QRPs applicable to all research designs by methodological approach (quantitative, qualitative, and mixed), type of research (descriptive, exploratory, hypothesis testing, and measurement instruments), and study design (observational, (quasi) experimental, systematic review, economic evaluation, case study, and meta-analyses). We used the STROBE checklist for observational studies in the reporting of this research.[19] Analyses were conducted using SPSS version 24.[20]

Patient and Public Involvement

This study was designed with the input provided by the participating HSR institutions at a consensus meeting at the onset of the study, and individual interviews with the directors/leaders of the 13 participating institutions. During a progress meeting with the participating institutions, preliminary (aggregated level) results were discussed to validate and complement the interpretation of findings.

Ethics approval

A waiver for ethical approval was obtained for this study from the medical ethics review committee at Amsterdam UMC. To avoid negative consequences for the authors of the included publications, each publication was assigned a unique identification number. Extracted data were entered in SPSS using this number to separate author information from the study data.

For peer review only

For peer review only - http://bmjopen.bmj.com/site/about/guidelines.xhtml

Results

Characteristics of included publications

Table 1 presents the characteristics of the 116 included publications from the 13 participating HSR institutions. To summarise, 54.3% of the publications were quantitative, 28.4% were qualitative, and 17.2% applied a mixed-methods approach. Sixteen percent of the publications were based on a published study protocol. The mean impact factor of the journals was 2.81, and the average number of authors was six.

Table 1: Characteristics of included publications

**Total (N= 116)**		**n (%)**
HSR domain	Policy	19 (16·4)
	Social factors	11 (9·5)
	Financing Systems	10 (8·6)
	Organizational structures & processes	43 (37·1)
	Health technologies	11 (9·5)
	Personal Behaviours	22 (19·0)
Methodological approach	Quantitative	63 (54·3)
	Qualitative	33 (28·4)
	Mixed methods	20 (17·2)
Type of research	Descriptive	31 (26·7)
	Exploratory	59 (50·9)
	Hypothesis testing	19 (16·4)
	Measurement instruments	5 (4·3)
	Other	2 (1·7)
Design	Observational	59 (50·9)
	(Quasi) experimental	9 (7·8)
	Systematic review	17 (14·7)
	Economic evaluation	5 (4·3)
	Other	1 (0·9)
Protocol published		19 (16·4)
Funder of study stated		98 (84·5)
Contributions stated		57 (49·1)
		Mean
Impact factor journal (n =93)		2·81 (SD 1·45)
Number of authors (n=116)		6·12 (SD 5·53)

Occurrence of QRPs per publication

Of the 116 HSR publications, the median number of QRPs per publication was six (interquartile range, 5·75), out of 35 possible QRPs. The distribution of the observed frequency of QRPs across publications is visualised in figure 1.

For peer review only

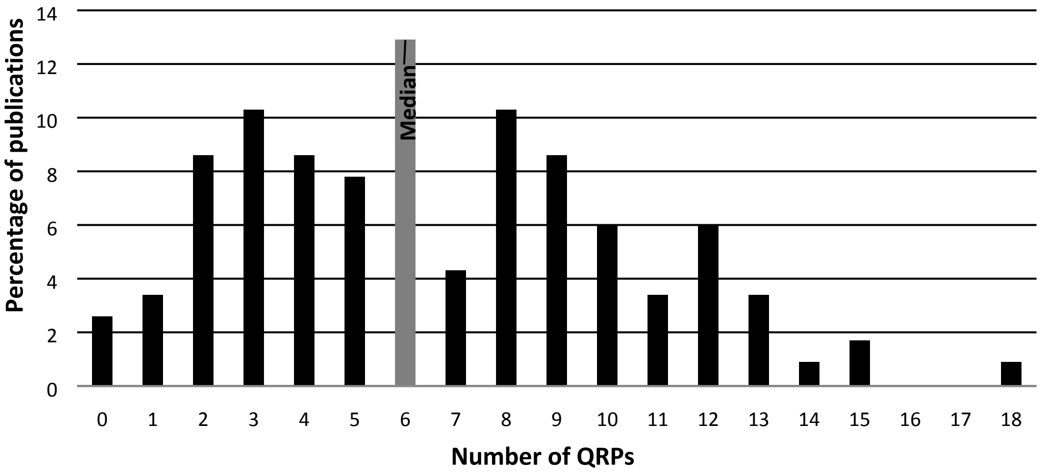

Figure 1: Percentage of HSR publications with number of observed QRPs in the reporting of messages and conclusions

Frequency of QRPs per type
For each of the QRPs, we counted how often they were identified in the included publications. Appendix 1 presents the percentage of occurrence per QRP type.

QRPs that occurred most frequently were:

- Implications for policy and practice do not adequately reflect the results in the context of the referenced literature (69·0%)*;
    - *In 50·0% of publications, no implications for policy and practice were mentioned, and in 19·0% of publications, implications were mentioned without adequate justification.
- Recommendations for policy and practice do not adequately reflect the results in the context of the referenced literature (65·5%)**;
    - **In 34·5% of publications, no recommendations for policy and practice were reported, and in 31·0% of publications, recommendations were mentioned without adequate justification.
- Contradicting evidence is poorly documented (63·8%);
- Conclusions do not adequately reflect the findings as presented in the results section (46·6%);
- Possible impact of the limitations on the results is not or poorly discussed (44·0%);
- Conclusions are not supported by the results as presented in the context of the referenced literature (43·1%).

QRPs that occurred least frequently were:

- The main source of evidence for supporting the results is based on the same underlying data (2·6%);
- Generalising findings to populations not included in the original sample is not justified (2·6%);
- Causative wording is used in the hypothesis/research question, although there is no theory to support causation (2·4%);
- Possible clinical relevance of statistically non-significant results is not addressed (2·4%);
- Generalising findings to time periods not included in the original study is not justified (0·0%).

Distribution of QRPs
Figure 2 shows the distribution of QRPs across publications. The horizontal axis shows the publications (n=116) ordered from the publication with the lowest (0) to the highest number (18) of observed QRPs in the reporting of messages and conclusions. The vertical axis shows the QRPs ordered from least (Generalisation to different time period) to most (Implications for practice are lacking) frequently observed. On the right vertical axis, the occurrence of QRPs is presented in number of QRPs counted. Each dot represents a QRP.

For peer review only - http://bmjopen.bmj.com/site/about/guidelines.xhtml

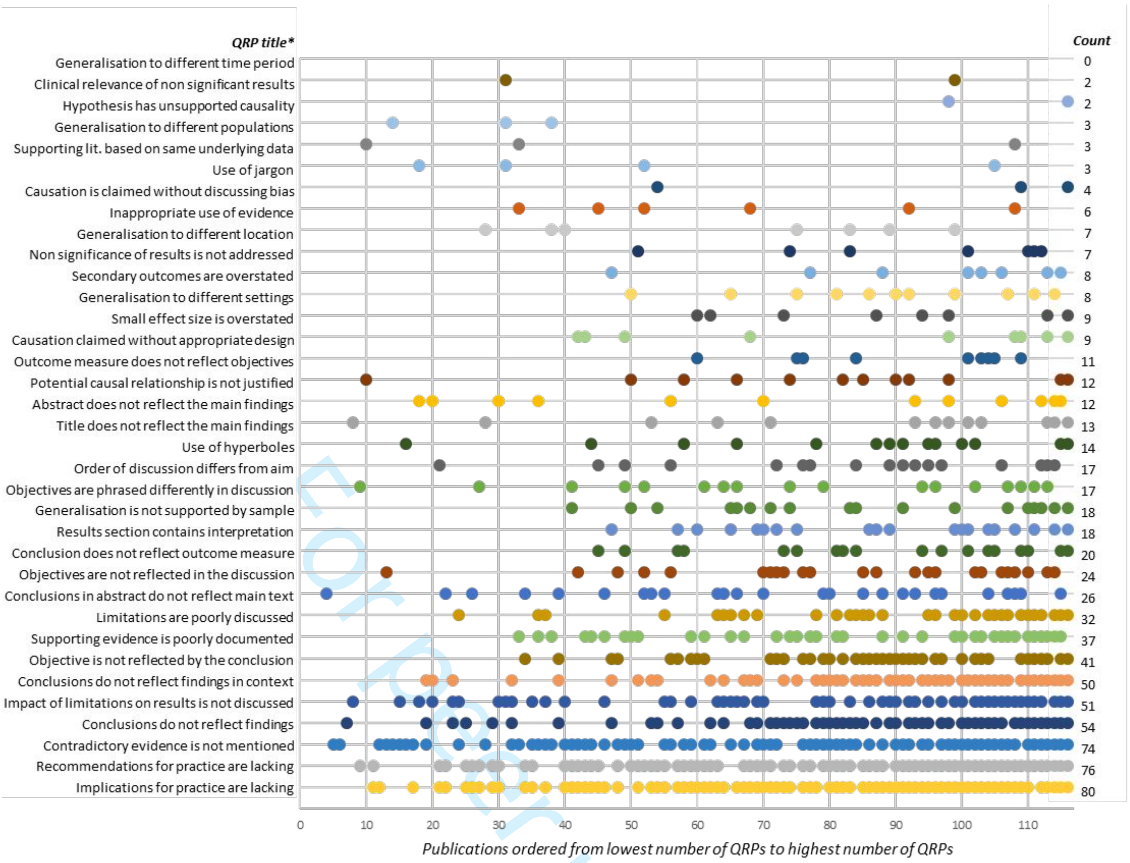

*The full QRP name is provided in appendix 1, table 3 ordered from least frequently found (Generalisation to different time period) to most frequently found (Implications for practice are lacking) QRP.'

Figure 2. Distribution of QRPs in the reporting of messages and conclusions across HSR publications, ordered from lowest to highest number of observed QRPs.

The difference in the number of QRPs by publication characteristics

Table 2 shows the associations between total number of QRPs (applicable to all study designs) and methodological approach (quantitative, qualitative, and mixed), type of research (descriptive, exploratory, hypothesis testing, and measurement instruments), and study design (observational, (quasi) experimental, systematic review, economic evaluation, case study, and meta-analyses).

No statistically significant differences in number of QRPs was found by type of research, methodological approach, or study design.

Table 2. Association between total number of QRPs and type of research, methodological approach, and study design

	Median	95% CI	p-value
Methodological approach			0·339
Quantitative	5	4·88 – 6·43	
Qualitative	6	4·98– 7·62	
Mixed methods	7	5·34 – 8·46	
Type of research			0·295
Descriptive	6	4·77 – 6·78	
Exploratory	7	5·76 – 7·60	
Hypothesis testing	4	3·40 – 6·81	
Measurement instruments	5	2·14 – 6·66	
Other	5	-33·12 – 43·12	
Study design			0·159
Observational	6	5·56 – 7·21	
(Quasi) experimental	3	2·07 – 5·71	
Systematic review	6	4·61 – 8·33	
Economic evaluation	4	1·61 – 7·59	
Case studies	6	4·71 – 8·01	
Meta-analyses	5	0·50 – 10·84	

For peer review only - http://bmjopen.bmj.com/site/about/guidelines.xhtml

For peer review only

Discussion

We explored the occurrence and nature of QRPs in the reporting of messages and conclusions in international scientific HSR publications authored by researchers from HSR institutions in the Netherlands, and examined the relationship between study type, methodology, and design and the occurrence of QRPs. Our results indicate that HSR publications have a median of six QRPs per publication. We identified most QRPs in the reporting of implications for policy and practice, recommendations for policy and practice, contradictory evidence, study limitations, and conclusions as based on the results and in the context of the literature. No significant associations between number of QRPs and type of study, study design, or methodological approach were identified.

Limitations and Strengths

We applied a broad and sensitive definition of 'questionable', for instance by considering the absence of contradictory evidence or the absence of implications and recommendations for policy and practice as a QRP. The choice to not present contradictory evidence does not defy current publication checklists, yet this practice may hinder interpretation of findings in the full context of evidence. If authors searched for contradictory evidence, but did not mention its absence, readers of the publication would not have any clues on its existence. With our broad definition encompassing 35 possible QRPs we bring to light the areas that offer possibilities for further enhancing publication practices in HSR. Consequently, this definition allows for a discussion in the field of HSR on the extent to which the identified QRPs are acceptable. This is an important strength of our applied approach.

Although we endeavoured to develop a reliable measurement instrument that would guide the review process, the instrument allowed latitude for the reviewer's interpretation. Consequently, a different group of reviewers might arrive at somewhat different scoring frequencies for observed QRPs. However, because we defined each QRP in detail, it is unlikely that there would be substantial differences in the overall distribution of different types of QRPs across publications. Our consensus method contains a degree of subjectivity, and there is the risk that one reviewer's opinion will dominate. To counteract this, NK and DK performed random checks on 10% of all assessments. By recording the motivation for every identified QRP, we supported the consistency of our measurement and justified our results. Because publications were selected based on the title, selection bias might have occurred. Considering we found no relationship between study characteristics and number of QRPs, it is unlikely that a different sample would have led to different results. Inevitably, reviewers sometimes assessed publications written by authors they knew professionally or personally, and as such, a positive view of a colleague's work might have led to underestimating the QRPs in these publications.

Our study results may be representative for HSR research publications internationally. Given the fact that publication in international journals is highly standardised in terms of language (English) and format, our findings can most likely be transferred to HSR communities in other countries.

Interpretation

In HSR publications, recommendations for policy and practice warrant most attention. A study can be conducted properly, using a sound design and appropriate methodology. However, making recommendations without adequate justification could lead to incorrect inferences in policy and the management of healthcare, and undermine society's confidence in science. [10,21-24]

Measures for safeguarding scientific soundness like those often used in biomedical research (eg, trial registration, open data policies, and an improved reporting and archiving infrastructure [25]) do not address reporting conclusions not supported by study results, and are not tailored to the observational and explorative designs most prevalent in HSR. Moreover, existing publication checklists address a report's completeness, but do not question the justification of the conclusions.[4] If we intend to improve the reporting of HSR conclusions and recommendations, we will need to better understand the factors that influence authors when reporting the discussion and conclusions section of a HSR publication eg, media pressure and relationships with funders.[5,6,8,26] Consequently, subsequent research can focus on what influences researchers when writing their scientific publications, and what factors play a role in the process from research design to the acceptance of a manuscript by a peer-reviewed journal.

A third of the HSR publications studied gave no recommendations for policy or practice, while another third did not provide an adequate justification for the recommendations. One could argue that HSR is an applied field of research, and that its ultimate goal should be to contribute to better health services and systems; researchers should therefore take responsibility for providing guidance to those who can act on the research findings instead

For peer review only

of leaving them empty-handed. On the other hand, health services researchers may feel more comfortable committing to a more traditional interpretation of the role of academics, refraining from normative judgement. If the latter is the dominant viewpoint, the HSR community needs to consider the role of scientific evidence in helping decision makers address the challenges they face, and informing policies and practices. Internationally, the HSR community has been promoting further strengthening of the link between HSR and practice.[27]

In biomedical research, research being "new" might contribute to a confused assessment of implications.[28] This problem is amplified in HSR, where there is a limited accumulation of evidence. HSR considers a larger range of contextual factors and stakeholders in politics or management. Moreover, HSR recommendations are often based on observational or exploratory research, which is considered to be weak evidence in biomedical circles (eg, the GRADE checklist).[29] Perhaps the norms determined by the biomedical research field make health services researchers hesitant to provide any implications or recommendations at all.

Implications and recommendations for policy and practice

The HSR field currently seems to adhere to the norms and expectations set by the biomedical field, even though HSR is multidisciplinary, and differences in approach and type of methodology pose serious challenges to observing these norms. Therefore, the HSR community needs to further define specific scientific norms appropriate to the field.

Scientific norms are developed through the forum of a scientific community.[30] This forum function is particularly strong in the Netherlands, where a community of HSR institutions work together closely. Our study was able to bring together the main Dutch academic and non-academic HSR institutions. Consequently, the results of our study help to facilitate critical reflection on the current state of research and encourage debate on how to systematically advance the reporting of messages and conclusions in HSR. Such a debate in the Dutch context is needed, given the attempts over the past decade by the Netherlands Organisation for Health Research and Development (ZonMw) to strengthen the link between research and practice. It would also be very timely, considering the ongoing, overarching Dutch research programme on responsible research practices funded by ZonMw, of which this study is a part. We recommend the HSR community to reflect on the questions our results bring forward: how do we include implications and recommendations for policy and practice in scientific publications?; how should we describe conclusions in context of literature with limited accumulation of evidence?; and what is the severity of the identified QRPs? Through this publication, we would like to urge journal editors and those working in the international field of HSR to join in this debate.

Conclusions

QRPs in the reporting of messages and conclusions occur frequently in peer-reviewed international scientific HSR publications from Dutch HSR institutions. These QRPs differ in severity and cannot always be qualified as wrongful, but they do 'raise questions'. To ensure the applicability of HSR research in policy and practice, the HSR field should reflect on scientific norms for the reporting of conclusions and the inclusion of recommendations for policy and practice. Our study can serve as an empirical basis for continuous critical reflection on the current state of research, and encourage debate on how to systematically advance the reporting of messages and conclusions in HSR.

For peer review only - http://bmjopen.bmj.com/site/about/guidelines.xhtml

Acknowledgements

We thank the Dutch HSR institutions that participated in this study: Erasmus MC, department of Public Health; Erasmus University, Erasmus School of Health Policy and Management; Leiden University Medical Centre, department of Medical Decision making and the department of Public Health and Primary Care; University Maastricht, Health Services Research group; the Netherlands Institute for Health Services Research (NIVEL); Radboud UMC, IQ healthcare; the National Institute for Public Health and the Environment (RIVM); University of Groningen, faculty of Economics and Business; Tilburg University, Social and Behavioural Sciences, Tranzo; Trimbos institute; University Medical Centre Utrecht, Julius Centre for Health Sciences and Primary Care; Amsterdam UMC – Vrije Universiteit Amsterdam, and the Amsterdam UMC - University of Amsterdam. Furthermore, we also would like to thank project advisers Anton Kunst, Thomas Plochg, and Patrick Romano. We thank Ellen Nolte, Enrique Bernal-Delgado, Michel Wensing, Oliver Groene, and Wilm Quentin for their valuable feedback on the definition of QRPs and the measurement instrument.

Role of funding source

This study was funded by ZonMw grant number 445001003. The funder had no role in the study design, in the collection, analysis, and interpretation of data, in the writing of the manuscript, and in the decision to submit the paper for publication. All authors had full access to the data during the conduct of this study and take responsibility for the integrity of the data and the data analysis.

Competing interests

All authors have completed the ICMJE uniform disclosure form at www.icmje.org/ coi_disclosure.pdf and declare: RG, NS, and DK received funding from ZonMw for the submitted work; TJ, JM and MB received no funding for this work; no financial relationships with any organisations that might have an interest in the submitted work in the previous three years; no other relationships or activities that could appear to have influenced the submitted work."

Contribution of authors

DK, NS, MB, RG and TJ designed the study. RG, TJ, and JM collected the data, and RG and JM analysed the data and RG drafted the manuscript. TJ, JM, MB, NS and DK were all involved in the interpretation of the results and were major contributors to the writing of the manuscript. All authors read and approved the final manuscript and are accountable for all aspects of the work.

Transparency statement

The lead author (DK) affirms that the manuscript is an honest, accurate, and transparent account of the study being reported; that no important aspects of the study have been omitted; and that any discrepancies from the study as originally planned have been explained.

Licence

The Corresponding Author has the right to grant on behalf of all authors and does grant on behalf of all authors, an exclusive licence (or non-exclusive licence for UK Crown and US Federal Government employees) on a worldwide basis to the BMJ Publishing Group Ltd, and its Licensees to permit this article (if accepted) to be published in the BMJ and any other BMJPGL products and to exploit all subsidiary rights, as set out in our licence.

For peer review only - http://bmjopen.bmj.com/site/about/guidelines.xhtml

References

1. Chalmers I, Glasziou P. Avoidable waste in the production and reporting of research evidence. *The Lancet* 2009; 374(9683): 86-9.
2. Moher D, Glasziou P, Chalmers I, et al. Increasing value and reducing waste in biomedical research: who's listening? *The Lancet* 2016; 387(10027): 1573-86.
3. Chiu K, Grundy Q, Bero L. 'Spin' in published biomedical literature: A methodological systematic review. *PLoS Biol* 2017; 15(9): e2002173.
4. Boutron I, Ravaud P. Misrepresentation and distortion of research in biomedical literature. *Proc Natl Acad Sci USA* 2018; 115(11): 2613-9.
5. Ioannidis JPA, Greenland S, Hlatky MA, et al. Increasing value and reducing waste in research design, conduct, and analysis. *The Lancet* 2014; 383(9912): 166-75.
6. Weingart P. Science and the media. *Res Policy* 1998; 27(8): 869-79.
7. Tijdink JK, Schipper K, Bouter LM, Maclaine Pont P, de Jonge J, Smulders YM. How do scientists perceive the current publication culture? A qualitative focus group interview study among Dutch biomedical researchers. *BMJ Open* 2016; 6(2): e008681.
8. Bouter LM. Commentary: Perverse Incentives or Rotten Apples? *Accountability in Research* 2015; 22(3): 148-61.
9. Porter ME. What Is Value in Health Care? *New England Journal of Medicine* 2010; 363(26): 2477-81.
10. Groen TP. Startdocument 'Systeemfalen' The Hague, 2013. Dutch
11. Horton R. The rhetoric of research. *BMJ* 1995; 310(6985): 985.
12. Boutron I, Dutton S, Ravaud P, Altman DG. Reporting and interpretation of randomized controlled trials with statistically nonsignificant results for primary outcomes. *JAMA* 2010; 303(20): 2058-64.
13. Ochodo EA, de Haan MC, Reitsma JB, Hooft L, Bossuyt PM, Leeflang MM. Overinterpretation and misreporting of diagnostic accuracy studies: evidence of "spin". *Radiology* 2013; 267(2): 581-8.
14. Altman DG, Simera I, Hoey J, Moher D, Schulz K. EQUATOR: reporting guidelines for health research. *Lancet* 2008; 371.
15. Stevens GA, Alkema L, Black RE, et al. Guidelines for Accurate and Transparent Health Estimates Reporting: the GATHER statement. *PLoS Med* 2016; 13(6): e1002056.
16. Tong A, Sainsbury P, Craig J. Consolidated criteria for reporting qualitative research (COREQ): a 32-item checklist for interviews and focus groups. *Int J Qual Health Care* 2007; 19(6): 349-57.
17. Juttmann RE. Het domein van gezondheidszorgonderzoek. In: Plochg T, Juttmann RE, Klazinga NS, Mackenbach JP, eds. Handboek gezondheidszorgonderzoek. Houten: Bohn Stafleu van Loghum; 2007: 22-23. Dutch.
18. Lohr KN, Steinwachs DM. Health services research: an evolving definition of the field. *Health Serv Res* 2002; 37(1): 7-9.
19. von Elm E, Altman DG, Egger M, et al. [The Strengthening the Reporting of Observational Studies in Epidemiology (STROBE) statement: guidelines for reporting observational studies]. *Rev Esp Salud Publica* 2008; 82(3): 251-9.
20. IBM Corp. Released 2016. IBM SPSS Statistics for Windows, Version 24.0. Armonk, NY: IBM Corp.
21. Lavis JN, Ross SE, Hurley JE, et al. Examining the role of health services research in public policymaking. *Milbank Q* 2002; 80(1): 125-54.
22. Hanney SR, Gonzalez-Block MA, Buxton MJ, Kogan M. The utilization of health research in policy-making: concepts, examples and methods of assessment. *Health Res Policy Syst* 2003; 1.
23. Kuruvilla S, Mays N, Walt G. Describing the impact of health services and policy research. *J Health Serv Res Policy* 2007; 12 Suppl 1: S1-23-31.
24. Burstein P. The impact of public opinion on public policy: A review and an agenda. *Polit Res Quart* 2003; 56(1): 29-40.
25. Glasziou P, Altman DG, Bossuyt P, et al. Reducing waste from incomplete or unusable reports of biomedical research. *The Lancet* 2014; 383(9913): 267-76.
26. Pittman P. Health Services Research in 2020: Data and Methods Needs for the Future. *Health Services Research* 2010; 45(5 Pt 2): 1431-41.
27. HSR-Europe. Health services research: helping tackle Europe's health care challenges. Policy Brief. Utrecht: NIVEL, Technische Universität Berlin, National Board of Health - Danish Centre for Health Technology Assessment, Academic Medical Centre - University of Amsterdam, the London School of Hygiene and Tropical Medicine, 2011.
28. Horton R. The hidden research paper. *JAMA* 2002; 287(21): 2775-8.
29. Atkins D, Best D, Briss PA, et al. Grading quality of evidence and strength of recommendations. *BMJ* 2004; 328(7454): 1490.
30. de Groot AD. Methodologie. Grondslagen van onderzoek en denken in de gedragswetenschappen. Assen: Van Gorcum; 1994. Dutch.

For peer review only - http://bmjopen.bmj.com/site/about/guidelines.xhtml

Appendix 1

Table 3. Occurrence of QRPs in the reporting of messages and conclusions in HSR publications (n=116), ordered from most frequently to least frequently occurring (%).

Questionable research practices (QRPs) in reporting messages and conclusions	% publications with QRP	% publications without QRP	% publications for which QRP not assessable
Implications for policy and practice do not adequately reflect the results in the context of the referenced literature.	**69·0	31·1	0·0
Recommendations do not adequately reflect the results in the context of the referenced literature.	***65·5	34·5	0·0
Contradicting evidence is poorly documented.	63·8	36·2	0·0
Conclusions do not adequately reflect the findings as presented in the results section.	46·6	51·7	1·7
Possible impact of the limitations on the results is not or poorly discussed.	44·0	56·0	0·0
Conclusions are not supported by the results as presented in the context of the referenced literature.	43·1	54·3	2·6
The conclusions do not adequately reflect the objectives of the study.	35·3	61·2	3·4
Supporting evidence is poorly documented.	31·9	68·1	0·0
Sources. direction and magnitude of bias are not or poorly discussed. or just listed without further discussion.	27·6	72·4	0·0
The conclusions in the abstract do not adequately reflect the conclusions in the main text.	22·4	75·0	2·6
The main results discussed in the discussion paragraph do not adequately address the original objectives/research questions as posed in the introduction.	20·7	75·9	3·4
The outcome measure used does not allow the conclusions that are stated. *	18·1	81·9	0·0
Lack of distinction between results and discussion. The results section contains elements of discussion and interpretation beyond the scope of explaining the results.	17·2	82·8	0·0
The sampling methodology does not allow the type of generalization provided.	15·5	84·5	0·0
The objectives/research questions of the study are differently phrased in the introduction and the discussion.	14·7	36·2	49·1
The order of presenting the results in de discussion is inconsistent with the ordering of the objectives/research questions as posed in the introduction.	14·7	75·0	10·3
Hyperboles and exaggerating adjectives are unjustifiably used	12·1	87·9	0·0
The title does not adequately reflect the main findings.	11·2	88·8	0·0
The abstract does not adequately reflect the main findings.	10·3	89·7	0·0
A potential causal relationship claimed in the discussion paragraph is not justified.	10·3	89·7	0·0
The outcome measure does not adequately reflect the objectives/research questions of the study. *	9·6	90·4	0·0
A causal relationship is claimed. although the research design is not appropriate to determine causation.	9·6	90·4	0·0
The relevance of statistically significant results with small effect size is overstated. *	9·6	90·4	0·0
Generalising findings to settings/institutions not included in the original study is not justified.	9·5	89·7	1·0

For peer review only - http://bmjopen.bmj.com/site/about/guidelines.xhtml

For peer review only

The conclusion/discussion distracts from main outcomes by overstating the relevance of secondary outcomes. *	8·4	91·6	0·0
Non-significant results are discussed without addressing significance.	8·4	91·6	0·0
Generalising findings to geographical locations not included in the original study is not justified.	6·0	94·0	0·0
Evidence is used inappropriately to support the findings.	5·2	94·9	0·0
A causal relationship is claimed although potential sources of bias and their potential impact on the findings were not discussed. *	3·6	96·4	0·0
Jargon. technical and complex language. that does not fit the journal audience. are used without properly explaining the meaning.	3·4	96·6	0·0
The main source of evidence for supporting the results is based on the same underlying data.	2·6	96·6	0·9
Generalising findings to populations not included in the original sample is not justified.	2·6	97·4	0·0
Causative wording is used in the hypothesis/research question, although there is no theory to support causation. *	2·4	97·6	0·0
Possible clinical relevance of statistically non-significant results is not addressed. *	2·4	97·6	0·0
Generalising findings to time periods not included in the original study is not justified.	0·0	100·0	0·0

** QRPs only applicable to quantitative research-based publications (n=83)*
*** 50 ·0% of publications did not mention implications for policy or practice.*
**** 34·5% of publications did not mention recommendations for policy or practice.*

For peer review only

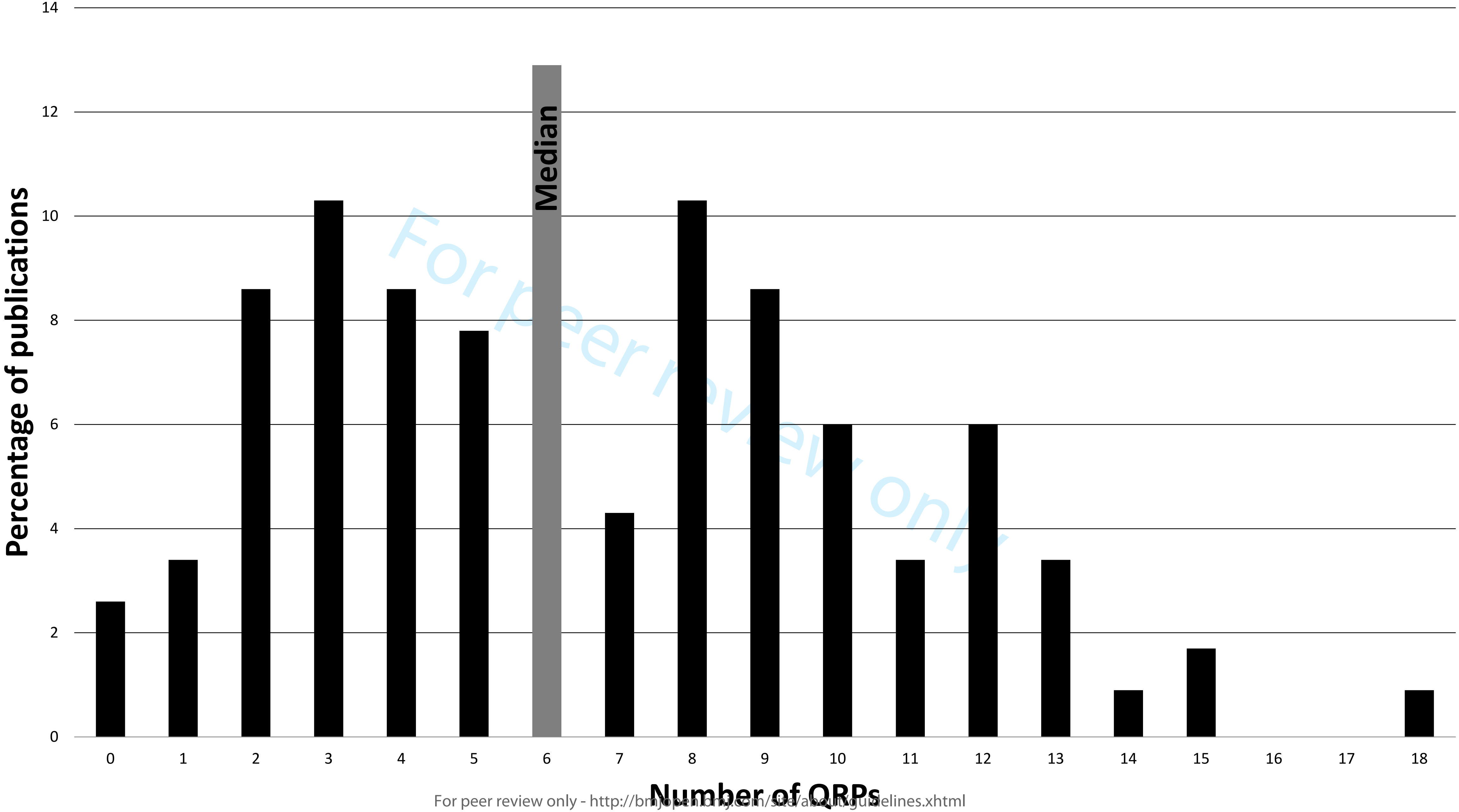

Percentage of publications
Number of QRPs
Median
0
2
4
6
8
10
12
14
0
1
2
3
4
5
6
7
8
9
10
11
12
13
14
15
16
17
18

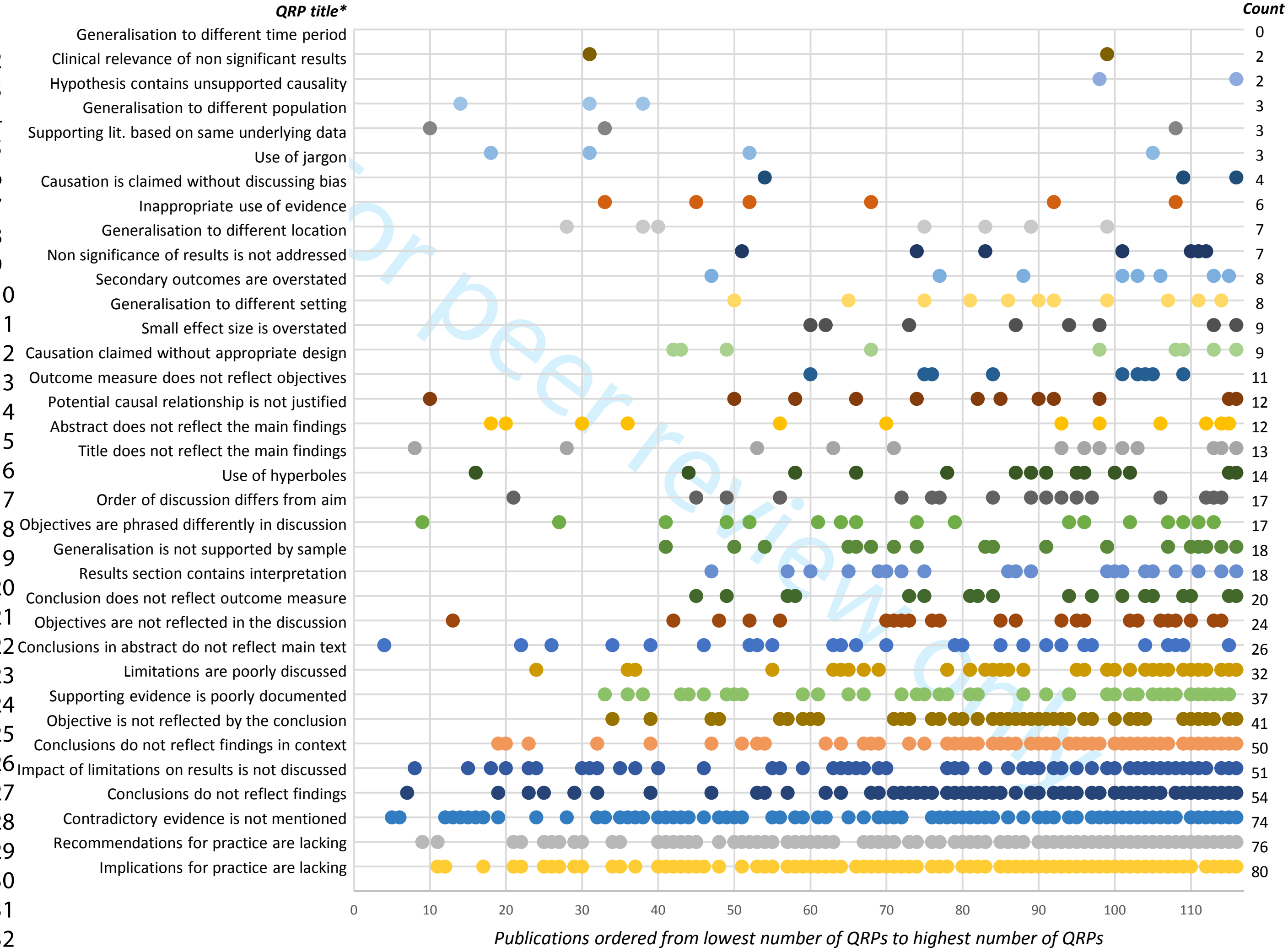

**The full QRP name is provided in appendix 1, table 3 ordered from least frequently found (Generalisation to different time period) to most frequently found (Implications for practice are lacking) QRP.'*

For peer review only - http://bmjopen.bmj.com/site/about/guidelines.xhtml

8 November 2018, DEF in Word format

Manuscript assessment & data extraction form (DEF)

Item

**1**	**Assessor**	
1.1	Name	
1.2	Assessor role	
1.3	Assessor code	

**2**	**General information**	
2.1	Title of the study	
2.2	Journal	
2.3	Number of authors	
2.4	HSR (main) domain	
2.5	Involved institutions	
2.6	Funder(s) of the study	
2.7	Role of funder in the study	
2.8	Contribution of authors is stated	
2.9	Competing interests	
2.10	EQUATOR checklist available in additional materials	
2.11	Trial registration/protocol published	

**3**	**Introduction**		**Specify**	**Evaluation/comments**
3.1	The objective(s) of the study are reported in the introduction			
3.2	The research question(s) are reported in the introduction			
3.3	The context of the study is explained			

**4**	**Methods**		**Specify**	**Evaluation/comments**
4.1	Methodological approach			
4.2	Type of research			
4.3	Research design			
4.4	Data source is reported			
4.5	Selection of participants/sample is reported			
4.6	Non-response is reported			
4.7	Size of the study is reported			
4.8	Main outcome measure(s) are reported			
4.9	Secondary outcome measure(s) are reported			
4.10	Independent variable(s) are reported			

For peer review only

8 November 2018, DEF in Word format

4.11	Description of quantitative and/or qualitative methods of analyses is reported			
4.12	Handling of missing data is reported			
4.13	Comparator is explained			

**5**	**Results**		**Specify**	**Evaluation/comments**
5.1	Tables properly represent results			
5.2	Graphs properly represent results			
5.3	(Statistical) uncertainty is reported			

**6**	**Questionable messages and conclusions**	**QRP observed** (0=no; 1= yes; -8 not applicable; -9=not assessable)	**Evaluation/ comments** (rationale for assessment of QRP)	**Consulted project member** (X= consulted for advice concerning methods, specifics about study, etc.)
6.1	*Conclusions and key messages do not adequately reflect the objectives, design and actual findings*			
6.1.1	The title does not adequately reflect the main findings.			
6.1.2	The abstract does not adequately reflect the main findings.			
6.1.3	The conclusions in the abstract do not adequately reflect the conclusions in the main text.			
6.1.4	The objectives/research questions of the study are differently phrased in the introduction and the discussion.			
6.1.5	The outcome measure does not adequately reflect the objectives/research questions of the study.			
6.1.6	The main results discussed in the discussion paragraph do not adequately address the original objectives/research questions as posed in the introduction.			
6.1.7	The order of presenting the results in de discussion is inconsistent with the ordering of the objectives/research questions as posed in the introduction.			
6.1.8	The conclusions do not adequately reflect the objectives of the study.			

For peer review

8 November 2018, DEF in Word format

6.1.9	The conclusions do not adequately reflect the findings as presented in the results paragraph.			
6.1.10	The outcome measure used does not allow the conclusions that are stated.			
6.1.11	The conclusion/discussion distracts from main outcomes by overstating the relevance of secondary outcomes.			
6.1.12	The conclusions are not supported by the results as presented in context of the referenced literature.			
6.1.13	Recommendations do not adequately reflect the results in context of the referenced literature.			
6.1.14	Implications for policy and practice do not adequately reflect the results in the context of the referenced literature.			
6.1.15	Lack of distinction between results and discussion. The results section contains elements of discussion and interpretation beyond the scope of explaining the results.			

6.2	*Main results are not or inadequately interpreted into the context of evidence*			
6.2.1	Supporting evidence is poorly documented.			
6.2.2	Contradicting evidence is poorly documented.			
6.2.3	Evidence is used inappropriately to support the findings (i.e. the argument is not supported by the actual message of the cited evidence). Will be measured as: Evidence seems to be used selectively to support the findings, given the title of the referenced evidence.			
6.2.4	The main source of evidence to support the results is based on the same underlying data.			

6.3	*Limitations are not adequately mentioned*			
6.3.1	Sources, direction and magnitude of bias are not or poorly discussed, or just listed without further discussion.			
6.3.2	The possible impact of the limitations on the results (i.e., magnitude and direction of any potential sources of bias) is not or poorly discussed.			

For peer review only - http://bmjopen.bmj.com/site/about/guidelines.xhtml

8 November 2018, DEF in Word format

6.4	*Unjustified generalisations*			
6.4.1	The sampling methodology does not allow the type of generalization provided.			
6.4.2	Generalization of findings to populations not included in the original sample is not justified.			
6.4.3	Generalization of findings to time periods not included in the original study is not justified.			
6.4.4	Generalization of findings to geographical locations not included in the original study is not justified.			
6.4.5	Generalization of findings to settings/institutions not included in the original study is not justified.			

6.5	*Unjustified causation*			
6.5.1	Causative wording is used in the hypothesis/research question, although there is no theory supporting causation.			
6.5.2	A causal relationship is claimed, although the research design is not appropriate to determine causation (methods lack control of potential confounding or systematic bias).			
6.5.3	A causal relationship is claimed although potential sources of bias and their potential impact on the findings were not discussed.			
6.5.4	A potential causal relationship claimed in the discussion paragraph is not justified.			

6.6	*Effect size*			
6.6.1	The relevance of statistically significant results with small effect size is overstated.			
6.6.2	The possible clinical relevance of statistically nonsignificant results is not addressed.			
6.6.3	Non-significant results are discussed without addressing significance			

6.7	*Inappropriate use of language*			
6.7.1	Hyperboles and exaggerating adjectives are unjustifiably used (such as: key, groundbreaking, ideal, excellent, great, brilliant,			

For peer review only

8 November 2018, DEF in Word format

	extraordinary, impressive, completely, absolutely, entirely, everywhere, everything, nothing, beyond any doubt, definitely).			
6.7.2	Jargon, technical and complex language, that does not fit the journal audience, are used without properly explaining the meaning.			

7 Miscellaneous

7.1	Overall qualitative evaluation of the study (e.g. quality, reporting style).		
7.2	Other comments.		

8 Advice needed from second assessor

8.1	About the contents of the article		
8.2	Second assessment recommended		

For peer review only

8 November 2018, DEF in Word format

Instructions per item

1 Assessor

1.1	Name	
1.2	Assessor role	
1.3	Assessor code	

2 General information

		**Instructions**
2.1	Title of the study	
2.2	Journal	
2.3	Number of authors	
2.4	HSR (main) domain	*Choose main discipline from list, add other disciplines in entry field*
2.5	Involved institutions	*List all-in*
2.6	Funder(s) of the study	
2.7	Role of funder in the study	*Copy funder declaration*
2.8	Contribution of authors is stated	
2.9	Competing interests	*Copy competing interest declaration*
2.10	EQUATOR checklist available in additional materials	
2.11	Trial registration/protocol published	*As mentioned in the article*

3 Introduction

3.1	The objective(s) of the study are reported in the introduction	
3.2	The research question(s) are reported in the introduction	
3.3	The context of the study is explained	

4 Methods

4.1	Methodological approach	
4.2	Type of research	
4.3	Research design	
4.4	Data source is reported	*e.g. registration, scientific or grey literature, survey data, interview data*
4.5	Selection of participants/sample is reported	*Selection of study enrolees also included case studies*
4.6	Non-response is reported	
4.7	Size of the study is reported	
4.8	Main outcome measure(s) are reported	

For peer review only - http://bmjopen.bmj.com/site/about/guidelines.xhtml

8 November 2018, DEF in Word format

4.9	Secondary outcome measure(s) are reported	
4.10	Independent variable(s) are reported	
4.11	Description of quantitative and/or qualitative methods of analyses is reported	
4.12	Handling of missing data is reported	
4.13	Comparator is explained	

5	**Results**	
5.1	Tables properly represent results	*Tables give a reflection of actual results instead of cherry picking*
5.2	Graphs properly represent results	*Scaling is appropriate*
5.3	(Statistical) uncertainty is reported	*Confidence intervals are provided for the main results*

6	**Questionable messages and conclusions**	**Instructions**
6.1	*Conclusions and key messages do not adequately reflect the objectives, design and actual findings*	
6.1.1	The title does not adequately reflect the main findings.	*Title includes a quote or statement that does not accurately reflect/refers to the main findings, or deviates from the findings.*
6.1.2	The abstract does not adequately reflect the main findings.	*The abstracts contents deviate from / contradict with the main findings in the article text. Messy writing is not considered a QRP. Specifically for the conclusion in the abstract, causative wording misses: the conclusion in the abstract suggests causation, although the conclusions as discussed in the discussion paragraph report correlation. For instance, it is an unbalanced representation of the main results by focussing on secondary findings, while reducing the importance of the main findings, or reflects cherry-picking from the most conspicuous results. Or the stated results in the abstract in qualitative studies do not appear in the main text.*
6.1.3	The conclusions in the abstract do not adequately reflect the conclusions in the main text.	*The conclusions in the abstract are short-sighted compared to the actual conclusions in the main text. Conclusions can be stated in the discussion paragraph and/or the conclusion paragraph.*

For peer review

6.1.4	The objectives/research questions of the study are differently phrased in the introduction and the discussion.	*When reporting objectives/research questions in the discussion. Different wording: does not need to include the exact wording, however the meaning/connotation should be similar. Different ordering of objectives/research questions.*
6.1.5	The outcome measure does not adequately reflect the objectives/research questions of the study.	*The objectives /research questions cannot be answered with the outcome measure that is studied*
6.1.6	The main results discussed in the discussion paragraph do not adequately address the original objectives/research questions as posed in the introduction.	*The research questions and/or objectives that were stated in the introduction section are not or only partly answered by the main results*
6.1.7	The order of presenting the results in de discussion is inconsistent with the ordering of the objectives/research questions as posed in the introduction.	*Not an actual QRP, but it does conflict with transparency in presenting the study's findings. If there's just one objective/research question, this item is not applicable (no structuring possible) and should be scored -8.*
6.1.8	The conclusions do not adequately reflect the objectives of the study.	*The objectives of the study are not met by the conclusions the study arrives at. Conclusions can be stated in the discussion paragraph and/or the conclusion paragraph. Either the study along the way shifted perspective, however no justification is provided. Or the write-up of the conclusions is flawed. Framing conclusion as extension to the discussion is not a QRP (undesirable, however beyond the scope if this indicator).*
6.1.9	The conclusions do not adequately reflect the findings as presented in the results paragraph.	*The conclusions deviate from the the main findings. Conclusions can be stated in the discussion paragraph and/or the conclusion paragraph. The conclusion section does often not contain actual conclusions. The actual conclusion is often presented in the discussion section. Hence, conclusions in the discussion section are considered conclusions as well. Concluding statements will be marked, those statements that are only used to frame results (emphasizing importance of the study) are not considered conclusions. Key messages (in a box as seperate section in some journals) are also considered conclusions.*<br>*For instance, it is an unbalanced representation of the main results by focussing on secondary findings, while reducing the importance of the main findings, or reflects cherry-picking from the most conspicuous*

For peer review only

8 November 2018, DEF in Word format

		results. If new results are presented in the discussion section, then this is a QRP. (Assessors should not recalculate results)
6.1.10	The outcome measure used does not allow the conclusions that are stated.	*For instance: the conclusions are about the quality of the health care system, whereas the outcome measure was 'satisfaction with home-care for elderly'*
6.1.11	The conclusion/discussion distracts from main outcomes by overstating the relevance of secondary outcomes.	*The main outcomes are ignored or their importance reduced, while favouring secondary outcomes. Most space is taken by discussing these secondary outcomes.*
6.1.12	The conclusions are not supported by the results as presented in context of the referenced literature.	*If the conclusion is not based on the results, but only on referenced literature, then this is noted as QRP (as aligns with 6.1.9). The extent of the conclusions is broader/more far fetching than the findings of the study, backed-up by discussed literature, justify. Conclusions can be stated in the discussion paragraph and/or the conclusion paragraph. For instance, a relationship between IV and DV is exaggerated. Conclusions cannot be stated based on referenced literature alone, main results are the fundament for the conclusions, that may be extended based on referenced literature.*
6.1.13	Recommendations do not adequately reflect the results in context of the referenced literature.	*Recommendations: what can/should be done with the studies findings? Recommendations are based on the results from the study, not only on the referenced literature. The extent of the recommendations is broader/more far fetching than the findings of the study, backed-up by discussed literature, justify. For instance, a relationship between IV and DV is exaggerated. QRP if no justification for the suggested recommendation is provided. QRP if no recommendation is provided.*
6.1.14	Implications for policy and practice do not adequately reflect the results in the context of the referenced literature.	*Implications: what are the consequences for policy and practice if the recommendations are followed-up? What would happen if the recommendations are carried out. (e.g. recommendations = implement the intervention in this setting, implication = the outcomes may improve by this much.) QRP if no justification for suggested implication is provided, QRP if no implication is provided. Originally: implications for*

For peer review

8 November 2018, DEF in Word format

		policy and practice are poorly mentioned. Instruction: implications for practise and policy are well-balanced and give actual meaning to the findings of the study in context of practice and/or policy.
6.1.15	Lack of distinction between results and discussion. The results section contains elements of discussion and interpretation beyond the scope of explaining the results.	*Applicable to all designs. Pilot included qualitative study, but also applies to quantitative studies. Results are placed in the context of literature beyond the theoretical model of the study.*

6.2	*Main results are not or inadequately interpreted into the context of evidence*	
6.2.1	Supporting evidence is poorly documented.	*Only limited evidence to support the main results is provided and only superficially discussed. No thorough reflection of the findings in perspective of supporting evidence.*
6.2.2	Contradicting evidence is poorly documented.	*Only limited evidence to oppose against the main results is provided and only superficially discussed. No thorough reflection of the findings in perspective of contradicting evidence.*
6.2.3	Evidence is used inappropriately to support the findings (i.e. the argument is not supported by the actual message of the cited evidence). Will be measured as: Evidence seems to be used selectively to support the findings, given the title of the referenced evidence.	*State inappropriately cited references, and explain why inappropriate: the evidence ascribed to the reference deviates from what could be assumed based on the title of the reference. Includes supporting results through self-citation (without further explanation of self-citation). Self-citation is not a QRP if clearly stated "in an earlier study we found..." If no references are used to support the results (QRP 6.2.1/2), then this is no QRP (QRP is avoided by not using literature), thus assessment is not possible ans should be scored -9.*
6.2.4	The main source of evidence to support the results is based on the same underlying data.	*Most supporting evidence is grounded in the same data source as was used for the reviewed study (not necessarely self-citing), inducing circularity in argumentation.*

6.3	*Limitations are not adequately mentioned*	
6.3.1	Sources, direction and magnitude of bias are not or poorly discussed, or just listed without further discussion.	*Are the (relevant) limitations mentioned? The implications of the study design, methodology, sampling, context, etc. for risk of biasing study findings are not thoroughly discussed.*

8 November 2018, DEF in Word format

6.3.2	The possible impact of the limitations on the results (i.e., magnitude and direction of any potential sources of bias) is not or poorly discussed.	*Is the impact of limitations discussed (if no limitations are mentioned then this is considered a QRP). The extent to which potential risks of bias affect the interpretation of the findings is not thoroughly discussed.*

6.4	*Unjustified generalisations*	
6.4.1	The sampling methodology does not allow the type of generalization provided.	*The sample is too specific, small, or flawed (for instance by attrition, selection bias) for the generalization that is made.*
6.4.2	Generalization of findings to populations not included in the original sample is not justified.	*The included sample is too specific, small or flawed (for instance by attrition, selection bias) and no or inadequate evidence is provided to support the generalization that is made. Population does not include geographical location (this is a separate QRP). Population includes population characteristics such as gender, ethnicity, age, etc.*
6.4.3	Generalization of findings to time periods not included in the original study is not justified.	*The characteristics of the included time period are too specific (for instance in election period, affecting the policy that was studied) and no or inadequate evidence is provided to support the generalization that is made*
6.4.4	Generalization of findings to geographical locations not included in the original study is not justified.	*The characteristics of the included igeographical location(s) are too specific to generalise to other geographical locations (for instance very urbanised area to rural setting) and no or inadequate evidence is provided to support the generalization that is made*
6.4.5	Generalization of findings to settings/institutions not included in the original study is not justified.	*The characteristics of the included institutions are too specific to generalise to other institutions (for instance hospital regulations to nursing homes) and no or inadequate evidence is provided to support the generalization that is made*

6.5	*Unjustified causation*	
6.5.1	Causative wording is used in the hypothesis/research question, although there is no theory supporting causation.	*Quantitative: hypothesis is not justified/allowed since there's no theory to support a causal relationship*
6.5.2	A causal relationship is claimed, although the research design is not appropriate to determine causation	*No causation based on the results of the present study may be assumed if no RCT is conducted… (or longitudinal cohort?)*

For peer review only - http://bmjopen.bmj.com/site/about/guidelines.xhtml

8 November 2018, DEF in Word format

	(methods lack control of potential confounding or systematic bias).	
6.5.3	A causal relationship is claimed although potential sources of bias and their potential impact on the findings were not discussed.	*No or inadequate discussion is included concerning the impact of potential sources of bias on the possible causation that was found in the results*
6.5.4	A potential causal relationship claimed in the discussion paragraph is not justified.	*When a causal relation may not be assumed solely based on the study's findings, no or inadequate supporting and contradicting evidence is used to discuss the possible causation that was found in the results.*

6.6	*Effect size*	
6.6.1	The relevance of statistically significant results with small effect size is overstated.	*Importance of findings is exaggerated. Although (some) results are statistically significant, the clinical/practical relevance is minor due to small effect size/causation is unlikely.*
6.6.2	The possible clinical relevance of statistically nonsignificant results is not addressed.	*Importance of findings is dismissed, since no statistical significance was reached. Although the findings reflect likely causation and non-significance was likely due to lack of power.*
6.6.3	Non-significant results are discussed without addressing significance	*Results are discussed as if they were significant, without addressing they are not, or what the uncertainty is.*

6.7	*Inappropriate use of language*	
6.7.1	Hyperboles and exaggerating adjectives are unjustifiably used (such as: key, groundbreaking, ideal, excellent, great, brilliant, extraordinary, impressive, completely, absolutely, entirely, everywhere, everything, nothing, beyond any doubt, definitely).	*The use of adjectives that exaggerate the relevance of the findings, conclusions and messages. Not actually counting adjectives, if one hyperbole is used and attracted the attention. Hyperbolic adjective use per se is no QRP, only in relation to results/conclusions, to exaggerate the study's findings.*
6.7.2	Jargon, technical and complex language, that does not fit the journal audience, are used without properly explaining the meaning.	*The journal audience is not properly addressed by the language used. Language use seems to be overly complex to impress or distract the reader.*

**7**	**Miscellaneous**	
7.1	Overall qualitative evaluation of the study (e.g. quality, reporting style).	*If a certain aspect impacts the answer to multiple questions, specify in "other comments". E.g. if the discussion section does not contain main results, then this item cannot be assessed.*

For peer review only

8 November 2018, DEF in Word format

7.2	Other comments.	

8 Advice needed from second assessor

8.1	About the contents of the article	*What advice is needed, state question.*
8.2	Second assessment recommended	*First assessor doubts about assessment and requests second opinion.*

For peer review only

STROBE Statement—checklist of items that should be included in reports of observational studies

	Item No	Recommendation	
**Title and abstract**	1	(*a*) Indicate the study's design with a commonly used term in the title or the abstract	No
		(*b*) Provide in the abstract an informative and balanced summary of what was done and what was found	Page 2
**Introduction**			
Background/rationale	2	Explain the scientific background and rationale for the investigation being reported	Page 3
Objectives	3	State specific objectives, including any prespecified hypotheses	Page 3
**Methods**			
Study design	4	Present key elements of study design early in the paper	Page 4
Setting	5	Describe the setting, locations, and relevant dates, including periods of recruitment, exposure, follow-up, and data collection	Page 4
Participants	6	(*a*) *Cohort study*—Give the eligibility criteria, and the sources and methods of selection of participants. Describe methods of follow-up<br>*Case-control study*—Give the eligibility criteria, and the sources and methods of case ascertainment and control selection. Give the rationale for the choice of cases and controls<br>*Cross-sectional study*—Give the eligibility criteria, and the sources and methods of selection of participants	Page 4
		(*b*) *Cohort study*—For matched studies, give matching criteria and number of exposed and unexposed<br>*Case-control study*—For matched studies, give matching criteria and the number of controls per case	n.a.
Variables	7	Clearly define all outcomes, exposures, predictors, potential confounders, and effect modifiers. Give diagnostic criteria, if applicable	Page 5
Data sources/ measurement	8*	For each variable of interest, give sources of data and details of methods of assessment (measurement). Describe comparability of assessment methods if there is more than one group	*Page 4-5*
Bias	9	Describe any efforts to address potential sources of bias	Page 4<br>Page 5
Study size	10	Explain how the study size was arrived at	Page 4
Quantitative variables	11	Explain how quantitative variables were handled in the analyses. If applicable, describe which groupings were chosen and why	Page 5
Statistical methods	12	(*a*) Describe all statistical methods, including those used to control for confounding	Page 5
		(*b*) Describe any methods used to examine subgroups	n.a.

For peer review only - http://bmjopen.bmj.com/site/about/guidelines.xhtml

and interactions	
(*c*) Explain how missing data were addressed	No missing data.
(*d*) *Cohort study*—If applicable, explain how loss to follow-up was addressed<br>*Case-control study*—If applicable, explain how matching of cases and controls was addressed<br>*Cross-sectional study*—If applicable, describe analytical methods taking account of sampling strategy	n.a.
(*e*) Describe any sensitivity analyses	n.a.

Continued on next page

For peer review only

**Results**			
Participants	13*	(a) Report numbers of individuals at each stage of study—eg numbers potentially eligible, examined for eligibility, confirmed eligible, included in the study, completing follow-up, and analysed	n.a.
		(b) Give reasons for non-participation at each stage	n.a.
		(c) Consider use of a flow diagram	n.a.
Descriptive data	14*	(a) Give characteristics of study participants (eg demographic, clinical, social) and information on exposures and potential confounders	Page 6
		(b) Indicate number of participants with missing data for each variable of interest	n.a.
		(c) *Cohort study*—Summarise follow-up time (eg, average and total amount)	n.a.
Outcome data	15*	*Cohort study*—Report numbers of outcome events or summary measures over time	n.a.
		Case-control study—Report numbers in each exposure category, or summary measures of exposure	
		Cross-sectional study—Report numbers of outcome events or summary measures	
Main results	16	(*a*) Give unadjusted estimates and, if applicable, confounder-adjusted estimates and their precision (eg, 95% confidence interval). Make clear which confounders were adjusted for and why they were included	Page 8
		(*b*) Report category boundaries when continuous variables were categorized	n.a.
		(*c*) If relevant, consider translating estimates of relative risk into absolute risk for a meaningful time period	n.a.
Other analyses	17	Report other analyses done—eg analyses of subgroups and interactions, and sensitivity analyses	n.a.
**Discussion**			
Key results	18	Summarise key results with reference to study objectives	Page 9
Limitations	19	Discuss limitations of the study, taking into account sources of potential bias or imprecision. Discuss both direction and magnitude of any potential bias	Page 9
Interpretation	20	Give a cautious overall interpretation of results considering objectives, limitations, multiplicity of analyses, results from similar studies, and other relevant evidence	Page 10-11
Generalisability	21	Discuss the generalisability (external validity) of the study results	Page 9
**Other information**			
Funding	22	Give the source of funding and the role of the funders for the present study and, if applicable, for the original study on which the present article is based	Page 11

*Give information separately for cases and controls in case-control studies and, if applicable, for exposed and unexposed groups in cohort and cross-sectional studies.

For peer review only

Note: An Explanation and Elaboration article discusses each checklist item and gives methodological background and published examples of transparent reporting. The STROBE checklist is best used in conjunction with this article (freely available on the Web sites of PLoS Medicine at http://www.plosmedicine.org/, Annals of Internal Medicine at http://www.annals.org/, and Epidemiology at http://www.epidem.com/). Information on the STROBE Initiative is available at www.strobe-statement.org.

For peer review only

For peer review only - http://bmjopen.bmj.com/site/about/guidelines.xhtml

BMJ Open

The occurrence and nature of questionable research practices in the reporting of messages and conclusions in international scientific Health Services Research publications: A structured assessment of publications authored by researchers in the Netherlands

Journal:	*BMJ Open*
Manuscript ID	bmjopen-2018-027903.R1
Article Type:	Research
Date Submitted by the Author:	07-Feb-2019
Complete List of Authors:	Gerrits, Reinie; Amsterdam UMC, University of Amsterdam, Amsterdam Public Health research institute, Public Health<br>Jansen, Tessa; Amsterdam UMC, University of Amsterdam, Amsterdam Public Health research institute, Public Health<br>Mulyanto, Joko; Amsterdam UMC, University of Amsterdam, Amsterdam Public Health research institute, Public Health<br>van den Berg, Michael; Amsterdam UMC, University of Amsterdam, Amsterdam Public Health research institute, Public Health<br>Klazinga, Niek; Amsterdam UMC, University of Amsterdam, Amsterdam Public Health research institute, Public Health<br>Kringos, Dionne; Amsterdam UMC, University of Amsterdam, Amsterdam Public Health research institute, Public Health
**Primary Subject Heading**:	Health services research
Secondary Subject Heading:	Evidence based practice, Research methods, Health policy
Keywords:	Health Services Research, Responsible Research Practices, Questionable Research Practices, Scientific Reporting

SCHOLARONE™
Manuscripts

The occurrence and nature of questionable research practices in the reporting of messages and conclusions in international scientific Health Services Research publications: A structured assessment of publications authored by researchers in the Netherlands

Reinie G. Gerrits1, MSc, Tessa Jansen1 MSc, Joko Mulyanto1 MD, Michael J. van den Berg1 PhD, Niek S. Klazinga1 PhD, Dionne S. Kringos1 PhD.

1 Amsterdam UMC, University of Amsterdam, Department of Public Health, Amsterdam Public Health research institute, P.O. Box 22660, 1100 DD Amsterdam, The Netherlands

Corresponding author
Reinie G. Gerrits
Address: P.O. Box 22660, 1100 DD Amsterdam, the Netherlands
Telephone: +31683555977
E-mail: r.g.gerrits@amc.uva.nl

For peer review only - http://bmjopen.bmj.com/site/about/guidelines.xhtml

Abstract

Objectives: Explore the occurrence and nature of questionable research practices (QRPs) in the reporting of messages and conclusions in international scientific HSR publications authored by researchers from health services research (HSR) institutions in the Netherlands.
Design: In a joint effort to assure the overall quality of HSR publications in the Netherlands, thirteen HSR institutions in the Netherlands participated in this study. Together with these institutions, we constructed and validated an assessment instrument covering 35 possible QRPs in the reporting of messages and conclusions. A QRP in the reporting of messages and conclusions in HSR is defined as *"to report, either intentionally or unintentionally, conclusions or messages that may lead to incorrect inferences and do not accurately reflect the objectives, the methodology or the results of the study."* Two reviewers independently assessed a random sample of 116 HSR articles authored by researchers from these institutions published in international peer-reviewed scientific journals in 2016.
Setting: Netherlands, 2016.
Sample: 116 international peer-reviewed HSR publications.
Main outcome measures: Median number of QRPs per publication, the percentage of publications with observed QRP frequencies, occurrence of specific QRPs, and difference in total number of QRPs by methodological approach, type of research, and study design.
Results: We identified a median of six QRPs per publication, out of 35 possible QRPs. QRPs occurred most frequently in the reporting of implications for practice, recommendations for practice, contradictory evidence, study limitations, and conclusions based on the results and in the context of the literature. We identified no differences in total number of QRPs in papers based on different methodological approach, type of research or study design.
Conclusions Given the applied nature of HSR, both the severity of the identified QRPs, and the recommendations for policy and practice in HSR publications warrant discussion. We recommend that the HSR field further define and establish its own scientific norms in publication practices to improve scientific reporting and strengthen the impact of HSR. The results of our study can serve as an empirical basis for continuous critical reflection on the reporting of messages and conclusions.

Funding: ZonMw grant number 445001003.

Strengths and limitations of the study

- Given the explorative nature of this study we applied a broad and sensitive definition of 'questionable research practices' (QRPs), that allows for the identification of QRPs previously overlooked in related assessments.
- This study describes an assessment of publications and is therefore able to detect QRPs that go unnoticed in survey studies that rely on self-report.
- Although we aimed to develop a reliable measurement instrument that would guide the review process, the instrument allowed latitude for the reviewer's interpretation.
- In our assessment method, we relied on consensus among assessors, which inevitably introduces some subjectivity. Independent assessments showed a consensus rate of >80% between assessors.
- Because publications were selected based on the title, selection bias might have occurred.

For peer review only - http://bmjopen.bmj.com/site/about/guidelines.xhtml

Introduction

In 2009, Chalmers and Glasziou estimated that 85% of research funding in biomedical sciences was wasted avoidably,[1] resulting in *The Lancet*'s series "Increasing value: reducing waste". This series has stirred the international scientific community, prompting funders, regulators, academic institutions, and scientific publishers to act. Funders of biomedical research have responded by organising conferences on research waste, and journal editors have initiated discussions on data sharing and open access.[2] While evidence for questionable research practices (QRPs) in biomedical sciences is mounting,[1] little is known about the occurrence and nature of QRPs in the policy- and management-oriented field of health services research (HSR). In particular, QRPs in the reporting of messages and conclusions have flown under the radar. The term 'questionable research practices' is commonly used to describe practices such as selective publication of results, concealing of conflicts of interests, and describing a hypothesis after finding significant results.[3] A questionable practice is not necessarily wrongful, but does 'raise questions'. In this study we further define the meaning of questionable research practices in the reporting of messages and conclusions in the field of HSR specifically.

The HSR field is an applied field of research, and produces evidence on topics such as co-payments, evaluation of quality improvement efforts, cost-effectiveness of medications, patient empowerment, therapy compliance, and effects of policies. Given the growing evidence for the prevalence of QRPs in the reporting of messages and conclusions in the biomedical field,[4,5] QRPs may also occur in the HSR field. In the biomedical field, a systematic review by Chiu et al. (2017) shows that estimates for the occurrence of questionable research practices in the interpretation of results in scientific publications vary from 10% of publications deriving discordant conclusions from study results to 100% of publications containing rhetorical practices resulting in spin, such as failure to compare risk to benefits in randomized controlled trials.[4]

Just like biomedical researchers, health services researchers are under pressure to publish in high-impact journals to increase their citation scores and attract media attention to augment their prestige and chances for future research funding and job security.[6-9] Unlike biomedical research, HSR findings are not easily generalised from one local or national health services setting to another, and messages and conclusions tend to be limited to a specific national context.[10] A broad spectrum of quantitative and qualitative methods is used in HSR, including designs that are less subject to strict codes of execution than randomized controlled trials, such as observational and case study designs. Furthermore, HSR has difficulty creating alignment between the construction of scientific knowledge and the implementation of that knowledge in policy and practice.[11] This combination of HSR specific characterics may result in a different set of QRPs in the reporting of a scientific study. The variation of designs other than RCTs, as is more common in the biomedical field, might invite unjustified claims of causality. Moreover, the context specific research may increase unjustified claims of generalisability, and the difficulty in translating knowledge to practice may result in unsupported recommendations or implications.

Although reporting in scientific publications is highly standardised, the discussion and conclusion sections offer researchers relative freedom when deriving messages and conclusions from study results.[5] We explored the occurrence and nature of QRPs in the reporting of messages and conclusions in international scientific HSR publications authored by researchers from HSR institutions in the Netherlands. We also examined the relationship between study type, methodology, and design and the occurrence of QRPs. With our study, we want to fuel the debate on fostering responsible messages and conclusions, and provide a basis for the discussion on QRPs in the international HSR field.

Methods

Setting

This study assessed scientific publications authored by researchers from 13 HSR groups, departments, or institutions (hereafter referred to as "HSR institutions") in the Netherlands, including both academic and non-academic institutions. These institutions all agreed to participate in an effort to assure the overall quality of HSR publications in the Netherlands.

Defining QRPs in the reporting of messages and conclusions in HSR

We conducted a literature review on QRPs in the reporting of messages and conclusions in biomedical research and HSR.[12-14] An initial definition of QRPs in the reporting of messages and conclusions in HSR was proposed and discussed at a consensus meeting with the directors/leaders of the 13 participating institutions. This was then validated through inputs from five leading international health services researchers (10 were invited; 50% non-response), and resulted in the following amended definition:

For peer review only - http://bmjopen.bmj.com/site/about/guidelines.xhtml

"To report, either intentionally or unintentionally, conclusions or messages that may lead to incorrect inferences and do not accurately reflect the objectives, the methodology or the results of the study."

Measurement instrument

We developed an extensive list of QRPs in the reporting of messages and conclusions. Items were based on the EQUATOR checklists[15] and earlier checklists for identifying "spin" (ie, "a way to distort science reporting without actually lying")[5] or other QRPs.[13,14,16,17] The proposed list of QRPs was reviewed, refined, and complemented using 14 semi-structured interviews with the directors/leaders and representatives (n=19) of the 13 participating HSR institutions. Next, the five participating international health services researchers provided email feedback on the list resulting from these interviews; the list was adapted accordingly, resulting in 35 possible QRPs in the reporting of messages and conclusions in HSR publications.

We developed a data extraction form in Excel that contained the list of QRPs and bibliometric information, and conducted a pilot to evaluate its feasibility and usability. In the pilot, two assessors (RG, TJ) independently assessed five international HSR publications to identify modifications needed to improve the form, and to align the interpretation of the items. The project group discussed the proposed modifications, resulting in the final version. The data extraction form, (supplementary material 1) and a methodology of the development of the data extraction form (supplementary material 2) is provided in the supplementary material.

Sample

We aimed to include ten HSR publications from each participating HSR institution. Inclusion criteria were: published in 2016 in an international peer-reviewed scientific journal, written in English, reporting HSR findings, and first- and/or last-authored by researchers affiliated with the respective HSR institution. As both the first author and the research institution are likely important factors influencing the occurrence of QRPs, only unique first authors were included in the publication. Moreover, not more than 10 publications per institution were included. This will ensure a maximum spread of authors and institutions across the sample.

Publication lists of the HSR institutions were retrieved either by searching publicly accessible online sources (eg, annual reports, open repositories or the research groups' website) or obtained from secretaries or librarians. All lists were verified by the respective HSR institutions. These lists included both HSR and non-HSR publications.

Two researchers (RG, TJ) selected all titles from the 13 publication lists that were likely to indicate empirical or systematic assessment studies in HSR. Publications were included if their title fitted the definitions of HSR by Juttmann (2007)[18] and Lohr & Steinwachs (2002).[19] These definitions are commonly used by HSR institutions (eg, in education) in the Netherlands. To select HSR studies, TJ and RG first individually selected titles from the publication lists. Next, RG and TJ compared their selections of titles and noted any differences. After completing the selection of the first HSR publications, selection was reviewed and approved by the research group (NK, DK, MB). TJ and RG then continued applying the selection method to the remaining publication lists. In a consensus meeting between TJ and RG, differences in selected titles were resolved by discussing its fit with the definition. Consensus was reached on all included publications.

The HSR publications (n=717) were assigned a random number. Per institution, the publications with unique first authors with the lowest assigned number were included in the sample. Three HSR institutions did not have enough publications with unique first authors, resulting in a selection of nine, eight, and two publications for these institutions. Furthermore, two publications were excluded during assessment because they concerned research protocols. These publications were replaced by another publication authored by the same institution. One publication was excluded because its methodology was considered incomprehensible by the reviewers. Ultimately, 116 HSR publications were included (16% of tot sample).

Assessment process

Two reviewers independently assessed all publications (RG and TJ or RG and JM). RG has primarily qualitative HSR experience and is trained in health economics. TJ and JM have primarily quantitative HSR experience and are trained in public health, management, economics, and law; and medicine, respectively.

The assessment started with a test phase. During this phase, agreements and disagreements in assessments of the first 30 publications were thoroughly discussed (by RG, TJ, NK, and DK) to increase the accuracy of the assessments; agreement between the two reviewers (TJ, RG) was 81% for the first 20 publications, which increased to 82% when assessing the next 10 publications. The notion emerged that it was necessary having two reviewers with complementary expertise assess each publication independently, followed by a consensus procedure and random check by the project leaders. RG trained the third reviewer (JM).

For peer review only - http://bmjopen.bmj.com/site/about/guidelines.xhtml

RG assessed all included publications, while TJ assessed the first 59 publications, and JM the remaining 57. All data were entered in the data extraction form. QRPs were coded as either 1, “present”; 0, “not present”; -8, “not applicable to this study” (primarily used for items not applicable for qualitative research); or -9, “not assessable”. To justify their assessments, the reviewers recorded their motivation for every identified QRP. At a later stage, QRPs in implications and recommendations for policy and practice were further refined into “not mentioned” if no implication or recommendation was included in the publications, and “not sufficiently justified”, if the authors did not provide any explanation for their implications or recommendations. The reviewers held regular consensus meetings (after review of 10 publications) to discuss and reach agreement on all identified QRPs.

During the consensus meetings, the reviewers compared their assessment of all items. Inconsistencies between the individually assessed QRPs were identified, discussed and adapted. Any remaining disagreements (n=2) were resolved by a senior researcher (DK). NK and DK each reassessed a random sample of six publications, so 10% of all included publications (n=12). As a result, two identified QRPs were retracted, and two QRPs were added to the reassessed publications.

Analysis

The characteristics of the included publications were described by calculating their occurrence with the percentage or mean number of publications.

We counted the total number of QRPs per publication, and the percentage of HSR publications with number of observed QRPs. The latter was visualised in a histogram. Occurrence of specific QRPs was calculated as a percentage of publications containing this particular QRP. The percentage of publications containing QRPs that were not applicable to qualitative research was calculated only for quantitative and mixed-methods-based publications (n = 83), (eg. the QRP: “The relevance of statistically significant results with small effect size is overstated” is only applicable to quantitative research).

We used a Kruskal-Wallis test to calculate the difference in total number of QRPs applicable to all research designs by methodological approach (quantitative, qualitative, and mixed), type of research (descriptive, exploratory, hypothesis testing, and measurement instruments), and study design (observational, (quasi) experimental, systematic review, economic evaluation, case study, and meta-analyses). We used the STROBE checklist for observational studies in the reporting of this research.[20] Analyses were conducted using SPSS version 24.[21]

Patient and Public Involvement

No patients were involved in this study. This study was designed with the input provided by the participating HSR institutions at a consensus meeting at the onset of the study, and individual interviews with the directors/leaders of the 13 participating institutions. During a progress meeting with the participating institutions, preliminary (aggregated level) results were discussed to validate and complement the interpretation of findings.

Ethics approval

A waiver for ethical approval was obtained for this study from the medical ethics review committee at Amsterdam UMC. To avoid negative consequences for the authors of the included publications, each publication was assigned a unique identification number. Extracted data were entered in SPSS using this number to separate author information from the study data.

For peer review only

For peer review only - http://bmjopen.bmj.com/site/about/guidelines.xhtml

Results

Characteristics of included publications

Table 1 presents the characteristics of the 116 included publications from the 13 participating HSR institutions. To summarise, 54.3% of the publications were quantitative, 28.4% were qualitative, and 17.2% applied a mixed-methods approach. Sixteen percent of the publications were based on a published study protocol. The mean impact factor of the journals was 2.81, and the average number of authors was six.

Table 1: Characteristics of included publications

**Total (N= 116)**		**n (%)**
HSR domain	Policy	19 (16·4)
	Social factors	11 (9·5)
	Financing Systems	10 (8·6)
	Organizational structures & processes	43 (37·1)
	Health technologies	11 (9·5)
	Personal Behaviours	22 (19·0)
Methodological approach	Quantitative	63 (54·3)
	Qualitative	33 (28·4)
	Mixed methods	20 (17·2)
Type of research	Descriptive	31 (26·7)
	Exploratory	59 (50·9)
	Hypothesis testing	19 (16·4)
	Measurement instruments	5 (4·3)
	Other	2 (1·7)
Design	Observational	59 (50·9)
	(Quasi) experimental	9 (7·8)
	Systematic review	17 (14·7)
	Economic evaluation	5 (4·3)
	Meta analyses	3 (2·6)
	Case study	22 (19·0)
	Other	1 (0·9)
Protocol published		19 (16·4)
Funder of study stated		98 (84·5)
Contributions stated		57 (49·1)
Number of included journals		80 (100·0)
		Mean
Impact factor journal (n=93 publications*)		2·81 (SD 1·45)
Number of authors (n=116)		6·12 (SD 5·53)

**Not all journals had an impact factor. Mean impact factor was calculated over 93 publications.*

Occurrence of QRPs per publication

Of the 116 HSR publications, the median number of QRPs per publication was six (interquartile range, 5·75), out of 35 possible QRPs. The distribution of the observed frequency of QRPs across publications is visualised in figure 1.

Figure 1: Percentage of HSR publications with number of observed QRPs in the reporting of messages and conclusions

Frequency of QRPs per type

For each of the QRPs, we counted how often they were identified in the included publications. Supplementary material 3, table 1 presents the percentage of occurrence per QRP type.

QRPs that occurred most frequently were:

- Implications for policy and practice do not adequately reflect the results in the context of the referenced literature (69·0%)*;

For peer review only - http://bmjopen.bmj.com/site/about/guidelines.xhtml

For peer review only

*In 50·0% of publications, no implications for policy and practice were mentioned, and in 19·0% of publications, implications were mentioned without adequate justification.

- Recommendations for policy and practice do not adequately reflect the results in the context of the referenced literature (65·5%)**;

**In 34·5% of publications, no recommendations for policy and practice were reported, and in 31·0% of publications, recommendations were mentioned without adequate justification.

- Contradicting evidence is poorly documented (63·8%);
- Conclusions do not adequately reflect the findings as presented in the results section (46·6%);
- Possible impact of the limitations on the results is not or poorly discussed (44·0%);
- Conclusions are not supported by the results as presented in the context of the referenced literature (43·1%).

QRPs that occurred least frequently were:

- The main source of evidence for supporting the results is based on the same underlying data (2·6%);
- Generalising findings to populations not included in the original sample is not justified (2·6%);
- Causative wording is used in the hypothesis/research question, although there is no theory to support causation (2·4%);
- Possible clinical relevance of statistically non-significant results is not addressed (2·4%);
- Generalising findings to time periods not included in the original study is not justified (0·0%).

Distribution of QRPs

Figure 2 shows the distribution of QRPs across publications. The horizontal axis shows the publications (n=116) ordered from the publication with the lowest (0) to the highest number (18) of observed QRPs in the reporting of messages and conclusions. The vertical axis shows the QRPs ordered from least (Generalisation to different time period) to most (Implications for practice are lacking) frequently observed. On the right vertical axis, the occurrence of QRPs is presented in number of QRPs counted. Each dot represents a QRP.

Figure 2. Distribution of QRPs in the reporting of messages and conclusions across HSR publications, ordered from lowest to highest number of observed QRPs.

The difference in the number of QRPs by publication characteristics

Table 2 shows the associations between total number of QRPs (applicable to all study designs) and methodological approach (quantitative, qualitative, and mixed), type of research (descriptive, exploratory, hypothesis testing, and measurement instruments), and study design (observational, (quasi) experimental, systematic review, economic evaluation, case study, and meta-analyses).

No statistically significant differences in number of QRPs was found by type of research, methodological approach, or study design.

Table 2. Association between total number of QRPs and type of research, methodological approach, and study design

	Median	95% CI	p-value
Methodological approach			0·339
Quantitative	5	4·88 – 6·43	
Qualitative	6	4·98– 7·62	
Mixed methods	7	5·34 – 8·46	
Type of research			0·295
Descriptive	6	4·77 – 6·78	
Exploratory	7	5·76 – 7·60	
Hypothesis testing	4	3·40 – 6·81	
Measurement instruments	5	2·14 – 6·66	
Other	5	-33·12 – 43·12	
Study design			0·159
Observational	6	5·56 – 7·21	
(Quasi) experimental	3	2·07 – 5·71	
Systematic review	6	4·61 – 8·33	
Economic evaluation	4	1·61 – 7·59	
Case studies	6	4·71 – 8·01	
Meta-analyses	5	0·50 – 10·84	

For peer review only - http://bmjopen.bmj.com/site/about/guidelines.xhtml

Discussion

We explored the occurrence and nature of QRPs in the reporting of messages and conclusions in international scientific HSR publications authored by researchers from HSR institutions in the Netherlands, and examined the relationship between study type, methodology, and design and the occurrence of QRPs. Our results indicate that HSR publications have a median of six QRPs per publication. We identified most QRPs in the reporting of implications for policy and practice, recommendations for policy and practice, contradictory evidence, study limitations, and conclusions as based on the results and in the context of the literature. No significant associations between number of QRPs and type of study, study design, or methodological approach were identified.

Limitations and Strengths

We applied a broad and sensitive definition of 'questionable', for instance by considering the absence of contradictory evidence or the absence of implications and recommendations for policy and practice as a QRP. The choice to not present contradictory evidence does not defy current publication checklists, yet this practice may hinder interpretation of findings in the full context of evidence. If authors searched for contradictory evidence, but did not mention its absence, readers of the publication would not have any clues on its existence. Knowledge on the occurrence of QRPs is often derived from survey studies, relying on self-report.3 These studies focus on the knowledge of consciously conducted, well-known QRPs. Our assessment approach allowed us to gain insight in less severe, more likely unconsciously occurring QRPs in the reporting of messages and conclusions specifically. The number of QRPs identified through assessment is generally higher than in studies relying on self-report.[3,4] With our broad definition encompassing 35 possible QRPs we bring to light the areas that offer possibilities for further enhancing publication practices in HSR. Consequently, this definition allows for a discussion in the field of HSR on the extent to which the identified QRPs are acceptable. This is an important strength of our applied approach.

Although we endeavoured to develop a reliable measurement instrument that would guide the review process, the instrument allowed latitude for the reviewer's interpretation. Consequently, a different group of reviewers might arrive at somewhat different scoring frequencies for observed QRPs. However, because we defined each QRP in detail, it is unlikely that there would be substantial differences in the overall distribution of different types of QRPs across publications. Our consensus method contains a degree of subjectivity, and there is the risk that one reviewer's opinion will dominate. To counteract this, NK and DK performed random checks on 10% of all assessments. By recording the motivation for every identified QRP, we supported the consistency of our measurement and justified our results. Because publications were selected based on the title, selection bias might have occurred. Considering we found no relationship between study characteristics and number of QRPs, it is unlikely that a different sample would have led to different results. Inevitably, reviewers sometimes assessed publications written by authors they knew professionally or personally, and as such, a positive view of a colleague's work might have led to underestimating the QRPs in these publications.

Our study results may be representative for HSR research publications internationally. Given the fact that publication in international journals is highly standardised in terms of language (English) and format, our findings can most likely be transferred to HSR communities in other countries.

Interpretation

In HSR publications, recommendations for policy and practice warrant most attention. A study can be conducted properly, using a sound design and appropriate methodology. However, making recommendations without adequate justification could lead to incorrect inferences in policy and the management of healthcare, and undermine society's confidence in science. [11,22-25]

Measures for safeguarding scientific soundness like those often used in biomedical research (eg, trial registration, open data policies, and an improved reporting and archiving infrastructure [26]) do not address reporting conclusions not supported by study results, and are not tailored to the observational and explorative designs most prevalent in HSR. Moreover, existing publication checklists address a report's completeness, but do not question the justification of the conclusions.[5] If we intend to improve the reporting of HSR conclusions and recommendations, we will need to better understand the factors that influence authors when reporting the discussion and conclusions section of a HSR publication eg, media pressure and relationships with funders.[6,7,9,27] Journals may have influence on the reporting of a study through control of the review process.[28] Moreover, research institutions may prevent the occurrence of QRPs by enhancing internal integrity, training in scientific writing and communication amongst researchers.[29] Consequently, subsequent research can focus on what

For peer review only - http://bmjopen.bmj.com/site/about/guidelines.xhtml

influences researchers when writing their scientific publications, and what factors play a role in the process from research design to the acceptance of a manuscript by a peer-reviewed journal.

A third of the HSR publications studied gave no recommendations for policy or practice, while another third did not provide an adequate justification for the recommendations. One could argue that HSR is an applied field of research, and that its ultimate goal should be to contribute to better health services and systems; researchers should therefore take responsibility for providing guidance to those who can act on the research findings instead of leaving them empty-handed. On the other hand, health services researchers may feel more comfortable committing to a more traditional interpretation of the role of academics, refraining from normative judgement. If the latter is the dominant viewpoint, the HSR community needs to consider the role of scientific evidence in helping decision makers address the challenges they face, and informing policies and practices. Internationally, the HSR community has been promoting further strengthening of the link between HSR and practice.[30]

In biomedical research, research being "new" might contribute to a confused assessment of implications.[31] This problem is amplified in HSR, where there is a limited accumulation of evidence. HSR considers a larger range of contextual factors and stakeholders in politics or management. Moreover, HSR recommendations are often based on observational or exploratory research, which is considered to be weak evidence in biomedical circles (eg, the GRADE checklist).[32] Perhaps the norms determined by the biomedical research field make health services researchers hesitant to provide any implications or recommendations at all.

Implications and recommendations for policy and practice

The HSR field currently seems to adhere to the norms and expectations set by the biomedical field, even though HSR is multidisciplinary, and differences in approach and type of methodology pose serious challenges to observing these norms. Therefore, the HSR community needs to further define specific scientific norms appropriate to the field.

Scientific norms are developed through the forum of a scientific community.[33] This forum function is particularly strong in the Netherlands, where a community of HSR institutions work together closely. Our study was able to bring together the main Dutch academic and non-academic HSR institutions. Consequently, the results of our study help to facilitate critical reflection on the current state of research and encourage debate on how to systematically advance the reporting of messages and conclusions in HSR. Such a debate in the Dutch context is needed, given the attempts over the past decade by the Netherlands Organisation for Health Research and Development (ZonMw) to strengthen the link between research and practice. It would also be very timely, considering the ongoing, overarching Dutch research programme on responsible research practices funded by ZonMw, of which this study is a part. We recommend the HSR community to reflect on the questions our results bring forward: how do we include implications and recommendations for policy and practice in scientific publications?; how should we describe conclusions in context of literature with limited accumulation of evidence?; and what is the severity of the identified QRPs? Through this publication, we would like to urge journal editors and those working in the international field of HSR to join in this debate. After establishing norms regarding these frequently occurring QRPs, journal editors and HSR institutions may contribute to the prevention of QRPs by implementing strategies tailored to HSR research specifically.

Conclusions

QRPs in the reporting of messages and conclusions occur frequently in peer-reviewed international scientific HSR publications from Dutch HSR institutions. These QRPs differ in severity and cannot always be qualified as wrongful, but they do 'raise questions'. To ensure the applicability of HSR research in policy and practice, the HSR field should reflect on scientific norms for the reporting of conclusions and the inclusion of recommendations for policy and practice. Our study can serve as an empirical basis for continuous critical reflection on the current state of research, and encourage debate on how to systematically advance the reporting of messages and conclusions in HSR.

For peer review only - http://bmjopen.bmj.com/site/about/guidelines.xhtml

Acknowledgements
We thank the Dutch HSR institutions that participated in this study: Erasmus MC, department of Public Health; Erasmus University, Erasmus School of Health Policy and Management; Leiden University Medical Centre, department of Medical Decision making and the department of Public Health and Primary Care; University Maastricht, Health Services Research group; the Netherlands Institute for Health Services Research (NIVEL); Radboud UMC, IQ healthcare; the National Institute for Public Health and the Environment (RIVM); University of Groningen, faculty of Economics and Business; Tilburg University, Social and Behavioural Sciences, Tranzo; Trimbos institute; University Medical Centre Utrecht, Julius Centre for Health Sciences and Primary Care; Amsterdam UMC – Vrije Universiteit Amsterdam, and the Amsterdam UMC - University of Amsterdam. Furthermore, we also would like to thank project advisers Anton Kunst, Thomas Plochg, and Patrick Romano. We thank Ellen Nolte, Enrique Bernal-Delgado, Michel Wensing, Oliver Groene, and Wilm Quentin for their valuable feedback on the definition of QRPs and the measurement instrument.

Role of funding source
This study was funded by ZonMw grant number 445001003. The funder had no role in the study design, in the collection, analysis, and interpretation of data, in the writing of the manuscript, and in the decision to submit the paper for publication. All authors had full access to the data during the conduct of this study and take responsibility for the integrity of the data and the data analysis.

Competing interests
None declared.

Contribution of authors
DK, NS, MB, RG and TJ designed the study. RG, TJ, and JM collected the data, and RG and JM analysed the data and RG drafted the manuscript. TJ, JM, MB, NS and DK were all involved in the interpretation of the results and were major contributors to the writing of the manuscript. All authors read and approved the final manuscript and are accountable for all aspects of the work.

Transparency statement
The lead author (DK) affirms that the manuscript is an honest, accurate, and transparent account of the study being reported; that no important aspects of the study have been omitted; and that any discrepancies from the study as originally planned have been explained.

Data sharing statement
The data concern the quantitative results from a review of scientific publications. Anonymized data is available till May 1st, 2024, by contacting d.s.kringos@amc.uva.nl.

Licence
The Corresponding Author has the right to grant on behalf of all authors and does grant on behalf of all authors, an exclusive licence (or non-exclusive licence for UK Crown and US Federal Government employees) on a worldwide basis to the BMJ Publishing Group Ltd, and its Licensees to permit this article (if accepted) to be published in the BMJ and any other BMJPGL products and to exploit all subsidiary rights, as set out in our licence.

For peer review only - http://bmjopen.bmj.com/site/about/guidelines.xhtml

References

1. Chalmers I, Glasziou P. Avoidable waste in the production and reporting of research evidence. *The Lancet* 2009; 374(9683): 86-9.
2. Moher D, Glasziou P, Chalmers I, et al. Increasing value and reducing waste in biomedical research: who's listening? *The Lancet* 2016; 387(10027): 1573-86.
3. Fanelli D. How Many Scientists Fabricate and Falsify Research? A Systematic Review and Meta-Analysis of Survey Data. *PLOS ONE 2009*; 4(5): e5738.
4. Chiu K, Grundy Q, Bero L. 'Spin' in published biomedical literature: A methodological systematic review. *PLoS Biol* 2017; 15(9): e2002173.
5. Boutron I, Ravaud P. Misrepresentation and distortion of research in biomedical literature. *Proc Natl Acad Sci USA* 2018; 115(11): 2613-9.
6. Ioannidis JPA, Greenland S, Hlatky MA, et al. Increasing value and reducing waste in research design, conduct, and analysis. *The Lancet* 2014; 383(9912): 166-75.
7. Weingart P. Science and the media. *Res Policy* 1998; 27(8): 869-79.
8. Tijdink JK, Schipper K, Bouter LM, Maclaine Pont P, de Jonge J, Smulders YM. How do scientists perceive the current publication culture? A qualitative focus group interview study among Dutch biomedical researchers. *BMJ Open* 2016; 6(2): e008681.
9. Bouter LM. Commentary: Perverse Incentives or Rotten Apples? *Accountability in Research* 2015; 22(3): 148-61.
10. Porter ME. What Is Value in Health Care? *New England Journal of Medicine* 2010; 363(26): 2477-81.
11. Groen TP. Startdocument 'Systeemfalen' The Hague, 2013. Dutch
12. Horton R. The rhetoric of research. *BMJ* 1995; 310(6985): 985.
13. Boutron I, Dutton S, Ravaud P, Altman DG. Reporting and interpretation of randomized controlled trials with statistically nonsignificant results for primary outcomes. *JAMA* 2010; 303(20): 2058-64.
14. Ochodo EA, de Haan MC, Reitsma JB, Hooft L, Bossuyt PM, Leeflang MM. Overinterpretation and misreporting of diagnostic accuracy studies: evidence of "spin". *Radiology* 2013; 267(2): 581-8.
15. Altman DG, Simera I, Hoey J, Moher D, Schulz K. EQUATOR: reporting guidelines for health research. *Lancet* 2008; 371.
16. Stevens GA, Alkema L, Black RE, et al. Guidelines for Accurate and Transparent Health Estimates Reporting: the GATHER statement. *PLoS Med* 2016; 13(6): e1002056.
17. Tong A, Sainsbury P, Craig J. Consolidated criteria for reporting qualitative research (COREQ): a 32-item checklist for interviews and focus groups. *Int J Qual Health Care* 2007; 19(6): 349-57.
18. Juttmann RE. Het domein van gezondheidszorgonderzoek. In: Plochg T, Juttmann RE, Klazinga NS, Mackenbach JP, eds. Handboek gezondheidszorgonderzoek. Houten: Bohn Stafleu van Loghum; 2007: 22-23. Dutch.
19. Lohr KN, Steinwachs DM. Health services research: an evolving definition of the field. *Health Serv Res* 2002; 37(1): 7-9.
20. von Elm E, Altman DG, Egger M, et al. [The Strengthening the Reporting of Observational Studies in Epidemiology (STROBE) statement: guidelines for reporting observational studies]. *Rev Esp Salud Publica* 2008; 82(3): 251-9.
21. IBM Corp. Released 2016. IBM SPSS Statistics for Windows, Version 24.0. Armonk, NY: IBM Corp.
22. Lavis JN, Ross SE, Hurley JE, et al. Examining the role of health services research in public policymaking. *Milbank Q* 2002; 80(1): 125-54.
23. Hanney SR, Gonzalez-Block MA, Buxton MJ, Kogan M. The utilization of health research in policy-making: concepts, examples and methods of assessment. *Health Res Policy Syst* 2003; 1.
24. Kuruvilla S, Mays N, Walt G. Describing the impact of health services and policy research. *J Health Serv Res Policy* 2007; 12 Suppl 1: S1-23-31.
25. Burstein P. The impact of public opinion on public policy: A review and an agenda. *Polit Res Quart* 2003; 56(1): 29-40.
26. Glasziou P, Altman DG, Bossuyt P, et al. Reducing waste from incomplete or unusable reports of biomedical research. *The Lancet* 2014; 383(9913): 267-76.
27. Pittman P. Health Services Research in 2020: Data and Methods Needs for the Future. *Health Services Research* 2010; 45(5 Pt 2): 1431-41.
28. Wager E, Middleton P. Technical editing of research reports in biomedical journals. Cochrane Database Syst Rev 2008; (4): MR000002.
29. Fanelli D, Costas R, Lariviere V. Misconduct Policies, Academic Culture and Career Stage, Not Gender or Pressures to Publish, Affect Scientific Integrity. PLoS One 2015; 10(6): e0127556.
30. HSR-Europe. Health services research: helping tackle Europe's health care challenges. Policy Brief. Utrecht: NIVEL, Technische Universität Berlin, National Board of Health - Danish Centre for Health

For peer review only

For peer review only - http://bmjopen.bmj.com/site/about/guidelines.xhtml

Technology Assessment, Academic Medical Centre - University of Amsterdam, the London School of Hygiene and Tropical Medicine, 2011.

31. Horton R. The hidden research paper. *JAMA* 2002; 287(21): 2775-8.

32. Atkins D, Best D, Briss PA, et al. Grading quality of evidence and strength of recommendations. *BMJ* 2004; 328(7454): 1490.

33. de Groot AD. Methodologie. Grondslagen van onderzoek en denken in de gedragswetenschappen. Assen: Van Gorcum; 1994. Dutch.

For peer review only

For peer review only - http://bmjopen.bmj.com/site/about/guidelines.xhtml

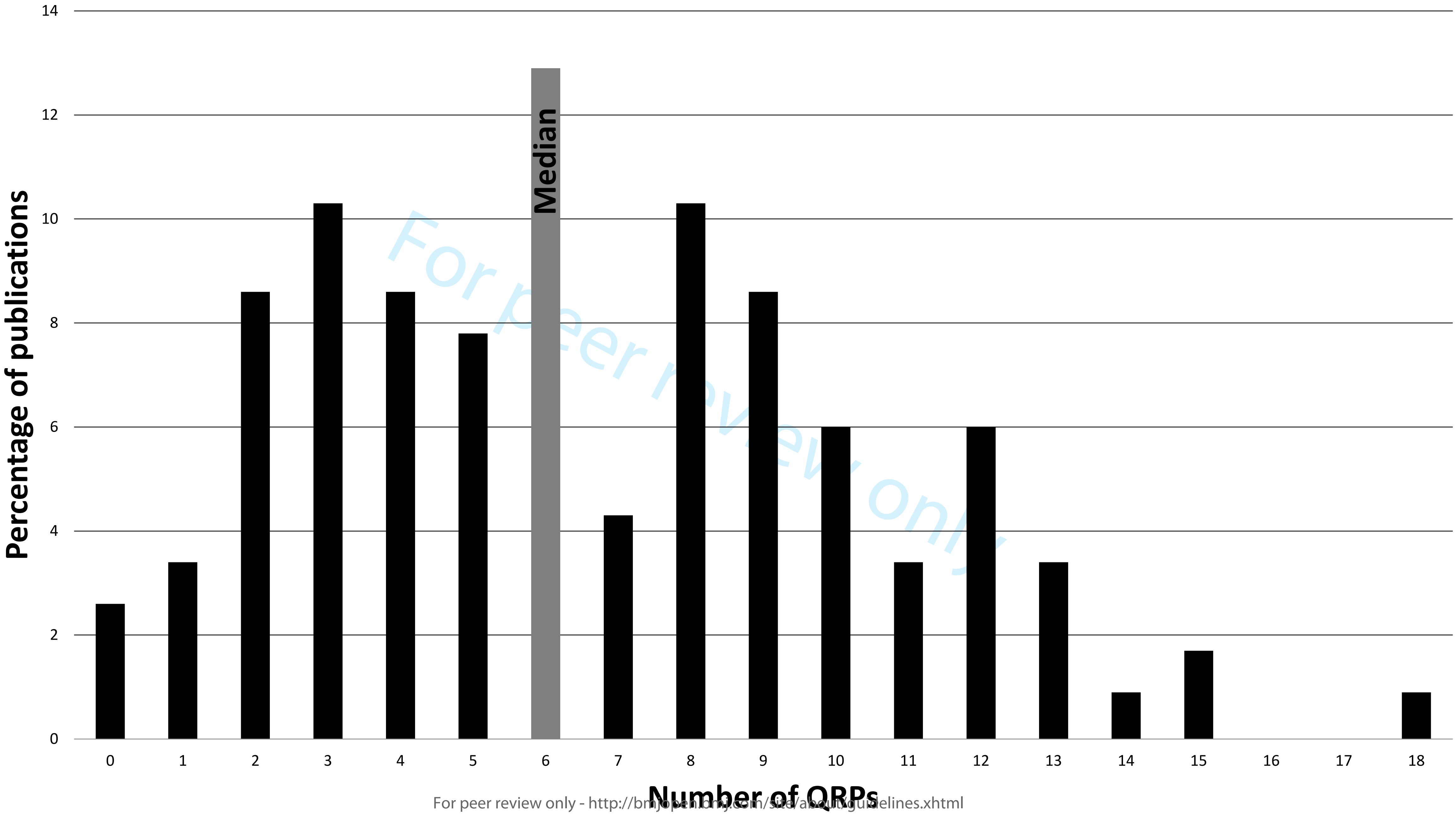

For peer review only

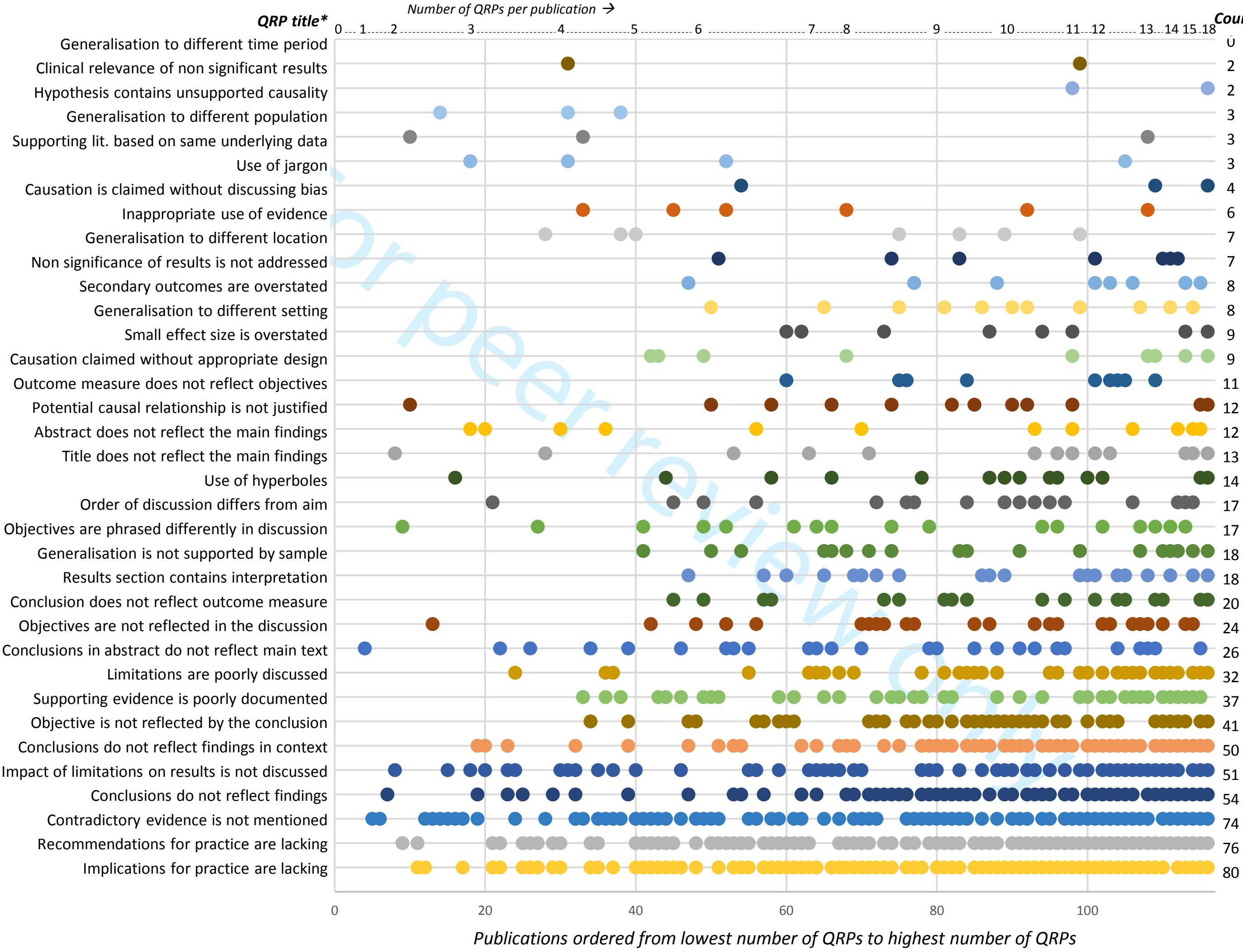

*The full QRP name is provided in supplementary material 3, table 1 ordered from least frequently found (Generalisation to different time period) to most frequently found (Implications for practice are lacking) QRP.'

For peer review only - http://bmjopen.bmj.com/site/about/guidelines.xhtml

8 November 2018, DEF in Word format

Manuscript assessment & data extraction form (DEF)

Item

1	**Assessor**	
1.1	Name	
1.2	Assessor role	
1.3	Assessor code	

2	**General information**	
2.1	Title of the study	
2.2	Journal	
2.3	Number of authors	
2.4	HSR (main) domain	
2.5	Involved institutions	
2.6	Funder(s) of the study	
2.7	Role of funder in the study	
2.8	Contribution of authors is stated	
2.9	Competing interests	
2.10	EQUATOR checklist available in additional materials	
2.11	Trial registration/protocol published	

3	**Introduction**		**Specify**	**Evaluation/comments**
3.1	The objective(s) of the study are reported in the introduction			
3.2	The research question(s) are reported in the introduction			
3.3	The context of the study is explained			

4	**Methods**		**Specify**	**Evaluation/comments**
4.1	Methodological approach			
4.2	Type of research			
4.3	Research design			
4.4	Data source is reported			
4.5	Selection of participants/sample is reported			
4.6	Non-response is reported			
4.7	Size of the study is reported			
4.8	Main outcome measure(s) are reported			
4.9	Secondary outcome measure(s) are reported			
4.10	Independent variable(s) are reported			

For peer review only

4.11	Description of quantitative and/or qualitative methods of analyses is reported			
4.12	Handling of missing data is reported			
4.13	Comparator is explained			

**5**	**Results**		**Specify**	**Evaluation/comments**
5.1	Tables properly represent results			
5.2	Graphs properly represent results			
5.3	(Statistical) uncertainty is reported			

**6**	**Questionable messages and conclusions**	**QRP observed** (0=no; 1= yes; -8 not applicable; -9=not assessable)	**Evaluation/ comments** (rationale for assessment of QRP)	**Consulted project member** (X= consulted for advice concerning methods, specifics about study, etc.)
6.1	*Conclusions and key messages do not adequately reflect the objectives, design and actual findings*			
6.1.1	The title does not adequately reflect the main findings.			
6.1.2	The abstract does not adequately reflect the main findings.			
6.1.3	The conclusions in the abstract do not adequately reflect the conclusions in the main text.			
6.1.4	The objectives/research questions of the study are differently phrased in the introduction and the discussion.			
6.1.5	The outcome measure does not adequately reflect the objectives/research questions of the study.			
6.1.6	The main results discussed in the discussion paragraph do not adequately address the original objectives/research questions as posed in the introduction.			
6.1.7	The order of presenting the results in de discussion is inconsistent with the ordering of the objectives/research questions as posed in the introduction.			
6.1.8	The conclusions do not adequately reflect the objectives of the study.			

For peer review only - http://bmjopen.bmj.com/site/about/guidelines.xhtml

6.1.9	The conclusions do not adequately reflect the findings as presented in the results paragraph.			
6.1.10	The outcome measure used does not allow the conclusions that are stated.			
6.1.11	The conclusion/discussion distracts from main outcomes by overstating the relevance of secondary outcomes.			
6.1.12	The conclusions are not supported by the results as presented in context of the referenced literature.			
6.1.13	Recommendations do not adequately reflect the results in context of the referenced literature.			
6.1.14	Implications for policy and practice do not adequately reflect the results in the context of the referenced literature.			
6.1.15	Lack of distinction between results and discussion. The results section contains elements of discussion and interpretation beyond the scope of explaining the results.			

6.2	*Main results are not or inadequately interpreted into the context of evidence*		

6.2.1	Supporting evidence is poorly documented.			
6.2.2	Contradicting evidence is poorly documented.			
6.2.3	Evidence is used inappropriately to support the findings (i.e. the argument is not supported by the actual message of the cited evidence). Will be measured as: Evidence seems to be used selectively to support the findings, given the title of the referenced evidence.			
6.2.4	The main source of evidence to support the results is based on the same underlying data.			

6.3	*Limitations are not adequately mentioned*		

6.3.1	Sources, direction and magnitude of bias are not or poorly discussed, or just listed without further discussion.			
6.3.2	The possible impact of the limitations on the results (i.e., magnitude and direction of any potential sources of bias) is not or poorly discussed.			

8 November 2018, DEF in Word format

6.4	*Unjustified generalisations*			
6.4.1	The sampling methodology does not allow the type of generalization provided.			
6.4.2	Generalization of findings to populations not included in the original sample is not justified.			
6.4.3	Generalization of findings to time periods not included in the original study is not justified.			
6.4.4	Generalization of findings to geographical locations not included in the original study is not justified.			
6.4.5	Generalization of findings to settings/institutions not included in the original study is not justified.			

6.5	*Unjustified causation*			
6.5.1	Causative wording is used in the hypothesis/research question, although there is no theory supporting causation.			
6.5.2	A causal relationship is claimed, although the research design is not appropriate to determine causation (methods lack control of potential confounding or systematic bias).			
6.5.3	A causal relationship is claimed although potential sources of bias and their potential impact on the findings were not discussed.			
6.5.4	A potential causal relationship claimed in the discussion paragraph is not justified.			

6.6	*Effect size*			
6.6.1	The relevance of statistically significant results with small effect size is overstated.			
6.6.2	The possible clinical relevance of statistically nonsignificant results is not addressed.			
6.6.3	Non-significant results are discussed without addressing significance			

6.7	*Inappropriate use of language*			
6.7.1	Hyperboles and exaggerating adjectives are unjustifiably used (such as: key, groundbreaking, ideal, excellent, great, brilliant,			

8 November 2018, DEF in Word format

	extraordinary, impressive, completely, absolutely, entirely, everywhere, everything, nothing, beyond any doubt, definitely).			
6.7.2	Jargon, technical and complex language, that does not fit the journal audience, are used without properly explaining the meaning.			

7 Miscellaneous

7.1	Overall qualitative evaluation of the study (e.g. quality, reporting style).		
7.2	Other comments.		

8 Advice needed from second assessor

8.1	About the contents of the article		
8.2	Second assessment recommended		

For peer review only

8 November 2018, DEF in Word format

Instructions per item

1 Assessor

1.1	Name	
1.2	Assessor role	
1.3	Assessor code	

2 General information

		**Instructions**
2.1	Title of the study	
2.2	Journal	
2.3	Number of authors	
2.4	HSR (main) domain	*Choose main discipline from list, add other disciplines in entry field*
2.5	Involved institutions	*List all-in*
2.6	Funder(s) of the study	
2.7	Role of funder in the study	*Copy funder declaration*
2.8	Contribution of authors is stated	
2.9	Competing interests	*Copy competing interest declaration*
2.10	EQUATOR checklist available in additional materials	
2.11	Trial registration/protocol published	*As mentioned in the article*

3 Introduction

3.1	The objective(s) of the study are reported in the introduction	
3.2	The research question(s) are reported in the introduction	
3.3	The context of the study is explained	

4 Methods

4.1	Methodological approach	
4.2	Type of research	
4.3	Research design	
4.4	Data source is reported	*e.g. registration, scientific or grey literature, survey data, interview data*
4.5	Selection of participants/sample is reported	*Selection of study enrolees also included case studies*
4.6	Non-response is reported	
4.7	Size of the study is reported	
4.8	Main outcome measure(s) are reported	

For peer review only

8 November 2018, DEF in Word format

4.9	Secondary outcome measure(s) are reported	
4.10	Independent variable(s) are reported	
4.11	Description of quantitative and/or qualitative methods of analyses is reported	
4.12	Handling of missing data is reported	
4.13	Comparator is explained	

5 Results

5.1	Tables properly represent results	*Tables give a reflection of actual results instead of cherry picking*
5.2	Graphs properly represent results	*Scaling is appropriate*
5.3	(Statistical) uncertainty is reported	*Confidence intervals are provided for the main results*

**6**	**Questionable messages and conclusions**	**Instructions**
6.1	*Conclusions and key messages do not adequately reflect the objectives, design and actual findings*	
6.1.1	The title does not adequately reflect the main findings.	*Title includes a quote or statement that does not accurately reflect/refers to the main findings, or deviates from the findings.*
6.1.2	The abstract does not adequately reflect the main findings.	*The abstracts contents deviate from / contradict with the main findings in the article text. Messy writing is not considered a QRP. Specifically for the conclusion in the abstract, causative wording misses: the conclusion in the abstract suggests causation, although the conclusions as discussed in the discussion paragraph report correlation. For instance, it is an unbalanced representation of the main results by focussing on secondary findings, while reducing the importance of the main findings, or reflects cherry-picking from the most conspicuous results. Or the stated results in the abstract in qualitative studies do not appear in the main text.*
6.1.3	The conclusions in the abstract do not adequately reflect the conclusions in the main text.	*The conclusions in the abstract are short-sighted compared to the actual conclusions in the main text. Conclusions can be stated in the discussion paragraph and/or the conclusion paragraph.*

For peer review only

8 November 2018, DEF in Word format

6.1.4	The objectives/research questions of the study are differently phrased in the introduction and the discussion.	*When reporting objectives/research questions in the discussion. Different wording: does not need to include the exact wording, however the meaning/connotation should be similar. Different ordering of objectives/research questions.*
6.1.5	The outcome measure does not adequately reflect the objectives/research questions of the study.	*The objectives /research questions cannot be answered with the outcome measure that is studied*
6.1.6	The main results discussed in the discussion paragraph do not adequately address the original objectives/research questions as posed in the introduction.	*The research questions and/or objectives that were stated in the introduction section are not or only partly answered by the main results*
6.1.7	The order of presenting the results in de discussion is inconsistent with the ordering of the objectives/research questions as posed in the introduction.	*Not an actual QRP, but it does conflict with transparency in presenting the study's findings. If there's just one objective/research question, this item is not applicable (no structuring possible) and should be scored -8.*
6.1.8	The conclusions do not adequately reflect the objectives of the study.	*The objectives of the study are not met by the conclusions the study arrives at. Conclusions can be stated in the discussion paragraph and/or the conclusion paragraph. Either the study along the way shifted perspective, however no justification is provided. Or the write-up of the conclusions is flawed. Framing conclusion as extension to the discussion is not a QRP (undesirable, however beyond the scope if this indicator).*
6.1.9	The conclusions do not adequately reflect the findings as presented in the results paragraph.	*The conclusions deviate from the the main findings. Conclusions can be stated in the discussion paragraph and/or the conclusion paragraph. The conclusion section does often not contain actual conclusions. The actual conclusion is often presented in the discussion section. Hence, conclusions in the discussion section are considered conclusions as well. Concluding statements will be marked, those statements that are only used to frame results (emphasizing importance of the study) are not considered conclusions. Key messages (in a box as seperate section in some journals) are also considered conclusions.*<br>*For instance, it is an unbalanced representation of the main results by focussing on secondary findings, while reducing the importance of the main findings, or reflects cherry-picking from the most conspicuous*

		results. *If new results are presented in the discussion section, then this is a QRP. (Assessors should not recalculate results)*
6.1.10	The outcome measure used does not allow the conclusions that are stated.	*For instance: the conclusions are about the quality of the health care system, whereas the outcome measure was 'satisfaction with home-care for elderly'*
6.1.11	The conclusion/discussion distracts from main outcomes by overstating the relevance of secondary outcomes.	*The main outcomes are ignored or their importance reduced, while favouring secondary outcomes. Most space is taken by discussing these secondary outcomes.*
6.1.12	The conclusions are not supported by the results as presented in context of the referenced literature.	*If the conclusion is not based on the results, but only on referenced literature, then this is noted as QRP (as aligns with 6.1.9). The extent of the conclusions is broader/more far fetching than the findings of the study, backed-up by discussed literature, justify. Conclusions can be stated in the discussion paragraph and/or the conclusion paragraph. For instance, a relationship between IV and DV is exaggerated. Conclusions cannot be stated based on referenced literature alone, main results are the fundament for the conclusions, that may be extended based on referenced literature.*
6.1.13	Recommendations do not adequately reflect the results in context of the referenced literature.	*Recommendations: what can/should be done with the studies findings? Recommendations are based on the results from the study, not only on the referenced literature. The extent of the recommendations is broader/more far fetching than the findings of the study, backed-up by discussed literature, justify. For instance, a relationship between IV and DV is exaggerated. QRP if no justification for the suggested recommendation is provided. QRP if no recommendation is provided.*
6.1.14	Implications for policy and practice do not adequately reflect the results in the context of the referenced literature.	*Implications: what are the consequences for policy and practice if the recommendations are followed-up? What would happen if the recommendations are carried out. (e.g. recommendations = implement the intervention in this setting, implication = the outcomes may improve by this much.) QRP if no justification for suggested implication is provided, QRP if no implication is provided. Originally: implications for*

For peer review only

		policy and practice are poorly mentioned. Instruction: implications for practise and policy are well-balanced and give actual meaning to the findings of the study in context of practice and/or policy.
6.1.15	Lack of distinction between results and discussion. The results section contains elements of discussion and interpretation beyond the scope of explaining the results.	*Applicable to all designs. Pilot included qualitative study, but also applies to quantitative studies. Results are placed in the context of literature beyond the theoretical model of the study.*

6.2	*Main results are not or inadequately interpreted into the context of evidence*	
6.2.1	Supporting evidence is poorly documented.	*Only limited evidence to support the main results is provided and only superficially discussed. No thorough reflection of the findings in perspective of supporting evidence.*
6.2.2	Contradicting evidence is poorly documented.	*Only limited evidence to oppose against the main results is provided and only superficially discussed. No thorough reflection of the findings in perspective of contradicting evidence.*
6.2.3	Evidence is used inappropriately to support the findings (i.e. the argument is not supported by the actual message of the cited evidence). Will be measured as: Evidence seems to be used selectively to support the findings, given the title of the referenced evidence.	*State inappropriately cited references, and explain why inappropriate: the evidence ascribed to the reference deviates from what could be assumed based on the title of the reference. Includes supporting results through self-citation (without further explanation of self-citation). Self-citation is not a QRP if clearly stated "in an earlier study we found..." If no references are used to support the results (QRP 6.2.1/2), then this is no QRP (QRP is avoided by not using literature), thus assessment is not possible ans should be scored -9.*
6.2.4	The main source of evidence to support the results is based on the same underlying data.	*Most supporting evidence is grounded in the same data source as was used for the reviewed study (not necessarely self-citing), inducing circularity in argumentation.*

6.3	*Limitations are not adequately mentioned*	
6.3.1	Sources, direction and magnitude of bias are not or poorly discussed, or just listed without further discussion.	*Are the (relevant) limitations mentioned? The implications of the study design, methodology, sampling, context, etc. for risk of biasing study findings are not thoroughly discussed.*

8 November 2018, DEF in Word format

6.3.2	The possible impact of the limitations on the results (i.e., magnitude and direction of any potential sources of bias) is not or poorly discussed.	*Is the impact of limitations discussed (if no limitations are mentioned then this is considered a QRP). The extent to which potential risks of bias affect the interpretation of the findings is not thoroughly discussed.*

6.4	*Unjustified generalisations*	
6.4.1	The sampling methodology does not allow the type of generalization provided.	*The sample is too specific, small, or flawed (for instance by attrition, selection bias) for the generalization that is made.*
6.4.2	Generalization of findings to populations not included in the original sample is not justified.	*The included sample is too specific, small or flawed (for instance by attrition, selection bias) and no or inadequate evidence is provided to support the generalization that is made. Population does not include geographical location (this is a separate QRP). Population includes population characteristics such as gender, ethnicity, age, etc.*
6.4.3	Generalization of findings to time periods not included in the original study is not justified.	*The characteristics of the included time period are too specific (for instance in election period, affecting the policy that was studied) and no or inadequate evidence is provided to support the generalization that is made*
6.4.4	Generalization of findings to geographical locations not included in the original study is not justified.	*The characteristics of the included igeographical location(s) are too specific to generalise to other geographical locations (for instance very urbanised area to rural setting) and no or inadequate evidence is provided to support the generalization that is made*
6.4.5	Generalization of findings to settings/institutions not included in the original study is not justified.	*The characteristics of the included institutions are too specific to generalise to other institutions (for instance hospital regulations to nursing homes) and no or inadequate evidence is provided to support the generalization that is made*

6.5	*Unjustified causation*	
6.5.1	Causative wording is used in the hypothesis/research question, although there is no theory supporting causation.	*Quantitative: hypothesis is not justified/allowed since there's no theory to support a causal relationship*
6.5.2	A causal relationship is claimed, although the research design is not appropriate to determine causation	*No causation based on the results of the present study may be assumed if no RCT is conducted… (or longitudinal cohort?)*

For peer review only

8 November 2018, DEF in Word format

	(methods lack control of potential confounding or systematic bias).	
6.5.3	A causal relationship is claimed although potential sources of bias and their potential impact on the findings were not discussed.	*No or inadequate discussion is included concerning the impact of potential sources of bias on the possible causation that was found in the results*
6.5.4	A potential causal relationship claimed in the discussion paragraph is not justified.	*When a causal relation may not be assumed solely based on the study's findings, no or inadequate supporting and contradicting evidence is used to discuss the possible causation that was found in the results.*

6.6	*Effect size*	
6.6.1	The relevance of statistically significant results with small effect size is overstated.	*Importance of findings is exaggerated. Although (some) results are statistically significant, the clinical/practical relevance is minor due to small effect size/causation is unlikely.*
6.6.2	The possible clinical relevance of statistically nonsignificant results is not addressed.	*Importance of findings is dismissed, since no statistical significance was reached. Although the findings reflect likely causation and non-significance was likely due to lack of power.*
6.6.3	Non-significant results are discussed without addressing significance	*Results are discussed as if they were significant, without addressing they are not, or what the uncertainty is.*

6.7	*Inappropriate use of language*	
6.7.1	Hyperboles and exaggerating adjectives are unjustifiably used (such as: key, groundbreaking, ideal, excellent, great, brilliant, extraordinary, impressive, completely, absolutely, entirely, everywhere, everything, nothing, beyond any doubt, definitely).	*The use of adjectives that exaggerate the relevance of the findings, conclusions and messages. Not actually counting adjectives, if one hyperbole is used and attracted the attention. Hyperbolic adjective use per se is no QRP, only in relation to results/conclusions, to exaggerate the study's findings.*
6.7.2	Jargon, technical and complex language, that does not fit the journal audience, are used without properly explaining the meaning.	*The journal audience is not properly addressed by the language used. Language use seems to be overly complex to impress or distract the reader.*

**7**	**Miscellaneous**	
7.1	Overall qualitative evaluation of the study (e.g. quality, reporting style).	*If a certain aspect impacts the answer to multiple questions, specify in "other comments". E.g. if the discussion section does not contain main results, then this item cannot be assessed.*

8 November 2018, DEF in Word format

7.2	Other comments.	

8 Advice needed from second assessor

8.1	About the contents of the article	*What advice is needed, state question.*
8.2	Second assessment recommended	*First assessor doubts about assessment and requests second opinion.*

For peer review only

February 5th, 2019

Supplement to the methods section of:

The occurrence and nature of questionable research practices in the reporting of messages and conclusions in international scientific Health Services Research publications: A structured assessment of publications authored by researchers in the Netherlands

Additional information to the methods of the development of the definition and measurement instrument for "questionable research practices in the reporting of messages and conclusions in scientific health services research publications"

This document describes the methods used to develop a definition of questionable research practices (QRPs) in the reporting of messages and conclusions, and to construct a measurement instrument that allows for the identification of questionable research practices in the reporting of messages and conclusions in Health Services Research (HSR).

Methodology
Methods included an explorative review of definitions in literature, a consultation meeting with the project group, institution/department leaders of Dutch HSR institutions and project advisors (n=13), semi-structured interviews with 13 HSR institutes (n=19) and an expert consultation (n=5).

Setting
13 HSR groups, departments, or institutions (hereafter referred to as "HSR institutions") in the Netherlands, including both academic and non-academic institutions participated in this study. These institutions all agreed to participate in an effort to assure the overall quality of HSR publications in the Netherlands.

Literature review
First, a literature review was conducted searching for existing definitions of questionable research practices in the reporting of conclusions and messages, and operationalisations of QRPs. Search terms included in different order and combination: 'questionable research practices', 'spin', 'over interpretation', 'discordant conclusions', 'QRPs', 'outcome reporting bias', 'questionable conclusions' and 'responsible conclusions'. Documents were included if they described methods to measure questionable research practices in scientific publication, or provided definitions of the above key terms. Referred documents that fit the criteria were also included in the review.

After identifying the main literature that suited our aim, we came to a preliminary definition of QRPs based on Boutron 2010, Ochobo 2013, and Horton 1995 [1-3].

An extensive list of possible types of QRPs in the reporting of messages and conclusions was developed, based on the EQUATOR checklists[4] and instruments from previous studies. For example, instruments for identifying 'spin', reporting of qualitative research and other QRPs such as [3,5-7]. Spin in this context refers to "a way to distort science reporting without actually lying")

Consultation meeting
Second, we presented the preliminary QRP definition and the first draft of items referring to QRPs (see page 3) during a consultation meeting of participating HSR institutions on 6 June 2017. The meeting lasted three hours, during which the research project and the preliminary definition and draft of QRP items was discussed. Representatives of the participating HSR institutions (n=7), project advisors (n=2) and project group members (n=4) attended the meeting. The attendees discussed their thoughts about the definition and its operationalisation. Detailed notes from this meeting were summarized and shared with the representatives of all participating institutes (including those who did not attend).

The central conclusion of the meeting was to focus on the 'measurability' of the QRPs. An important consideration in developing the instrument for the assessment of scientific publication is to focus on the possibility to measure the QRPs. Therefore, the focus should be on QRPs that can be quantified. These should be distinguished from QRPs that, although possibly important, are not quantifiable.

For peer review only - http://bmjopen.bmj.com/site/about/guidelines.xhtml

February 5th, 2019

Semi-structured interviews

Third, we conducted fourteen semi-structured interviews with nineteen leaders/representatives of the thirteen HSR institutions. These representatives had to have a clear overview of the process of reporting research in their institute. One of the institutions was represented by two separate departments, hence two representatives were separately interviewed. Three interviews were conducted with both the institute leader and a second representative. One of the interviews included three representatives of an institution. The aim of the interviews was to discuss our draft of QRP items and identify additional measurable QRPs in the reporting of messages and conclusions in HSR, explore potential causes of QRPs in messages and conclusions, and to discuss experiences of the institute leaders with these QRPs. A semi-structured interview guide was developed by the project team (see page 4-5). During the interview, we presented the interviewees with a draft of QRP items. The draft list was iteratively adjusted, i.e. after each interview we drafted a new version including the findings of the previous interviews.

Interviewees were approached through e-mail to schedule an appointment. Two researchers conducted the interviews of which thirteen took place at the participating institutions and one interview took place in a public space. During the first interview, both researchers were present to align the approach. The remainder of the interviews were equally divided between them. The interviews lasted one hour. In concordance with ethical guidelines, the goal of the interview was explained at the start of the interview and permission to audio-record the interview was obtained.

With the support of the recordings, a report was written and shared with the interviewees for validation. All interviewees confirmed the reports, after mostly minor edits to the report. From the interview reports, we drew up a new draft of the list of QRP items (see page 6-7). In the research group, we specifically paid attention to correct wording of the QRPs.

Expert consultation

Fourth, ten leading international health services researchers were asked to provide feedback on this list of QRP items. These HSR experts were invited through e-mail in which we explained the aim of the study, and included the definition of QRPs and the list of QRP items. Five experts provided their comments to the items. Five experts did not respond after a reminder, or indicated not having time to review the QRP items. Feedback was summarized, and comments were used to adapt the QRP definition and list of QRP items.

Measurement instrument

We developed the measurement instrument in Excel format by taking items from earlier developed checklists (EQUATOR and COREQ) and the list of QRPs. The measurement instrument was completed after a final consensus meeting of the research group. The measurement instrument exists of three sections: 1) bibliographic information of the publication (eg. funder, journal, number of authors), 2) basic methodological information (eg. included population, analyses method) and 3) possible QRPs in messages and conclusions. A pilot was conducted to assess the feasibility and usability of the instrument. In the pilot, two project members independently assessed five international HSR publications to identify modifications needed to improve the items in the instrument, and to align the interpretation of the items. The project group discussed the proposed modifications, resulting in the final version: the data extraction form (see supplementary material 1.)

For peer review only - http://bmjopen.bmj.com/site/about/guidelines.xhtml

February 5th, 2019

List of possible questionable research practices presented during the consultation meeting and the interviews

With each interview, new QRP's were added to the list which were then presented during the next interview.

Definition: Questionable reporting of messages and conclusions:

> *"The use of reporting, from whatever motive, consciously or unconsciously, to make conclusions or messages weaker or stronger than results justify."*

Potential

1. Poorly set results into context of totality of evidence
2. No mention of contradictory evidence
3. Discrepancy between the aim of the study and the conclusion
4. No reporting objectives, aim or research question
5. Concealing limitations
6. Lack of transparency of methods used
7. *Not reporting a hypothesis*
8. Selective reporting of results in conclusion
9. No mention of [statistical] uncertainty

Actual

1. Discrepancy between the title, abstract and the article
2. Describing unjustified causation
3. Inappropriate citing
4. Authorial rhetoric
5. Misleading graphs and tables
6. Unjustified generalisations (mismatch between study population, sex, geographical entities and time period)
7. *Stating the [intervention/measure] is beneficial despite statistically nonsignificant difference for the primary outcome*
8. *Distract the reader from statistically nonsignificant results*
9. Not explaining the comparator/context of the intervention

For peer review only

February 5th, 2019

Interview guide used during the semi-structured interviews (in Dutch)

Interviewprotocol eerste consultatieronde juni/juli 2017

Toelichting op het interview

Het doel van het ZonMw Project is om te komen tot aanbevelingen ter bevordering van verantwoord rapporteren over gezondheidszorgonderzoek (responsible conclusions and messages in health services research).

Hoewel de projectleiding primair bij het AMC ligt, is het binnen het project nadrukkelijk een gedeelde verantwoordelijkheid van alle dertien betrokken instituten om te komen tot voorstellen ter bevordering van verantwoord rapporteren van gezondheidszorgonderzoek. We houden in deze eerste fase interviews met de hoofden en vertegenwoordigers van de betrokken HSR instituten. Tijdens het interview worden de volgende onderwerpen besproken:

1) Potentiele oorzaken van QRPs in het vormen van conclusies en berichten
2) Het meten van QRP in conclusies en berichten in HSR
3) Uw ervaring met Responsible en Questionable Research Practices.

Het interview zal 1 uur in beslag nemen. Indien u daarvoor toestemming geeft, zal het gesprek worden opgenomen, en notities van het gesprek zullen worden uitgewerkt. Het gespreksverslag zal vervolgens ter verificatie aan u worden voorgelegd. Het gesprek wordt vertrouwelijk behandeld; alleen de onderzoekers op dit project zullen inzicht hebben in de inhoud van dit gesprek. De rapportage van de bevindingen zal op geaggregeerd niveau plaatsvinden. Uitspraken zullen daarbij niet-herleidbaar tot persoon en/of instituut worden gerapporteerd.

Wij zullen eerst onze bevindingen tot nu toe kort toelichten, en vervolgens verdergaan met het interview.

1) Bevindingen startbijenkomst
    a. Positieve start
    b. Nadruk op betrokkenheid alle instituten (veel feedbackloops)

For peer review only - http://bmjopen.bmj.com/site/about/guidelines.xhtml

February 5th, 2019

Vragen

Probleem onderkenning

1. Acht U het zinvol om naar ongeoorloofd rapporteren (QRP) bij gezondheidszorg onderzoek in NL te kijken?
    a. Indien ja, waarom denkt U dat het een reëel probleem is
    In de omvang, neemt het toe of af?

Beleid & structuur (wat doe u in uw rol als instituutshoofd om dit probleem aan te pakken)

2. Kunt u iets vertellen over de wijze waarop er binnen uw instituut wordt omgegaan met het stimuleren van verantwoord rapporteren van (HSR) onderzoek?
    a. Is er specifiek beleid op het verantwoord rapporteren van HSR?
    Indien ja, kunt u dat toelichten? (Open doorvragen, voorbeelden)

    b. Is er specifiek beleid, procedures, werkwijzen om QRPs in rapporteren van HSR te voorkomen?
    Indien ja, kunt u dat toelichten? (Open doorvragen, voorbeelden)

Definitie QRPs

Tot nu toe hebben wij de volgende QRPs geïdentificeerd in het rapporteren van onderzoek in wetenschappelijke publicaties [lijst QRPs].

3. Bent u het eens met (de formulering van) deze QRPs en heeft u opmerkingen en aanvullingen op deze lijst?
    a. Wat wilt u veranderen en of toevoegen?
4. Wij willen een keuze maken uit specifieke, goed te meten QRPs in het rapporteren van conclusies en berichten in HSR. Welke QRPs voldoen naar uw mening aan deze criteria?

Ervaringen

5. Wat zijn uw ervaringen met het rapporteren van resultaten van gezondheidszorgonderzoek?
    a. Wat gaat naar uw ervaring goed?
    b. Wat zijn in uw ervaringen knelpunten? Kunt u voorbeelden noemen waarin deze knelpunten naar voren kwamen?

Toelichting framework

Op dit moment hebben wij de factoren van invloed QRP als volgt weergeven [framework].

6. Zijn dit volgens u juiste factoren?
7. Wat zijn naar uw mening (nog meer) belangrijke factoren van invloed op RRPs en QRPs?
8. Welke factoren zou u als eerste aanpakken? Waarom deze factoren? Wat verwacht u daarvan?

Afsluiting

9. Heeft U nog aanvullende suggesties hoe in samenwerking met de andere gezondheidszorgonderzoeksinstituten in NL kan worden bijgedragen aan verantwoord rapporteren?

For peer review only - http://bmjopen.bmj.com/site/about/guidelines.xhtml

February 5th, 2019

QRP list and comment form used for expert consultation

Experts provided comments in the comment boxes

Questionable reporting of conclusions and messages in Health Services Research

Expert consultation

Definition:

"To frame, from whatever motive, consciously or unconsciously, conclusions or messages as an answer to the research question that are not justified by the results"

[Comments concerning definition]

Measuring questionable reporting of conclusions & messages

Title, abstract, main text, and conclusions do not align

1.1. The title does not align with the main text.
1.2. The abstract does not align with the main text.
1.3. The conclusions in the abstract do not align with the conclusions in the main text.
1.4. The objectives/research questions of the study are differently phrased in the introduction. and the discussion.

[Comments concerning category 1]

Conclusions do not reflect the objectives and results properly

2.1. The main results in the discussion do not follow from the research questions.
2.2. The conclusions do not align with the results in the main text.
2.3. The order of presenting the results is inconsistent with the research questions.
2.4. The conclusion/discussion distracts from main outcomes by overstating the relevance of secondary outcomes.
2.5. The relevance of statistically significant results with small effect sizes is overstated.
2.6. Possible clinical relevance of statistically insignificant results is not addressed.
2.7. The conclusions do not reflect the objectives of the study.
2.8. The conclusions are not supported by the results in context of the discussed literature.
2.9. Recommendations do not follow from the results and discussed literature.
2.10. Implications for policy and practice are poorly mentioned.

[Comments concerning category 2]

Main results are poorly put into the context of evidence

3.1. Supporting evidence is poorly mentioned.
3.2. Contradicting evidence is poorly mentioned.
3.3. Citations are used inappropriately to support the conclusions (i.e. the actual message of the cited source does not align with the conclusion it should support).
3.4. Self-citations or studies based on the same data are the main source of supporting evidence.

[Comments concerning category 3]

For peer review only - http://bmjopen.bmj.com/site/about/guidelines.xhtml

February 5th, 2019

Limitations are not properly mentioned

4.1. Limitations are discussed only superficially (for instance only on one level, e.g. the measurement level, design, sample).
4.2. No sources of bias are mentioned.
4.3. The possible effect of the limitations on the results is not discussed.

[Comments concerning category 4]

Unjustified generalizations

5.1. The time of data collection does not align with the time for which the conclusions are presented.
5.2. The study sample does not align with the population the conclusions are generalized to.
5.3. No justifications are offered for generalizations:
  5.3.1. In time.
  5.3.2. In geographical location.
  5.3.3. To setting/institution.

[Comments concerning category 5]

Unjustified causation

6.1. Causative wording that is used that is not allowed by the study design.
6.2. A causal relationship is claimed without mentioning any theoretical explanation of the relation.

[Comments concerning category 6]

Inappropriate language use

7.1. Hyperboles and exaggerating adjectives are used without justification (such as: ideal, excellent, great, brilliant, extraordinary, impressive, completely, absolutely, entirely, everywhere, everything, nothing, beyond any doubt, definitely).
7.2. Jargon, technical and complex language are used without properly explaining the meaning.

[Comments concerning category 7]

For peer review only

February 5th, 2019

References

1. Boutron I, Dutton S, Ravaud P, Altman DG: 'Spin' in reports of randomized controlled trials with nonstatistically significant primary outcomes. 6th International Congress on Peer Review and Biomedical Publication, Vancouver. (Accessed 30 March 2010), [http://www.ama-assn.org/public/peer/abstracts_2009.html#30].
2. Horton R. The rhetoric of research. *BMJ* 1995; **310**(6985): 985.
3. Ochodo EA, de Haan MC, Reitsma JB, Hooft L, Bossuyt PM, Leeflang MM. Overinterpretation and misreporting of diagnostic accuracy studies: evidence of "spin". *Radiology* 2013; **267**(2): 581-8.
4. Altman DG, Simera I, Hoey J, Moher D, Schulz K. EQUATOR: reporting guidelines for health research. *Lancet* 2008; **371**.
5. Stevens GA, Alkema L, Black RE, et al. Guidelines for Accurate and Transparent Health Estimates Reporting: the GATHER statement. *PLoS Med* 2016; **13**(6): e1002056.
6. Tong A, Sainsbury P, Craig J. Consolidated criteria for reporting qualitative research (COREQ): a 32-item checklist for interviews and focus groups. *Int J Qual Health Care* 2007; **19**(6): 349-57.
7. Boutron I, Dutton S, Ravaud P, Altman DG. REporting and interpretation of randomized controlled trials with statistically nonsignificant results for primary outcomes. *JAMA* 2010; **303**(20): 2058-64.

For peer review only

For peer review only - http://bmjopen.bmj.com/site/about/guidelines.xhtml

Supplementary material 3

Table 1. Occurrence of QRPs in the reporting of messages and conclusions in HSR publications (n=116), ordered from most frequently to least frequently occurring (%).

**Questionable research practices (QRPs) in reporting messages and conclusions**	**% publications with QRP**	**% publications without QRP**	**% publications for which QRP not assessable**
Implications for policy and practice do not adequately reflect the results in the context of the referenced literature.	**69·0	31·1	0·0
Recommendations do not adequately reflect the results in the context of the referenced literature.	***65·5	34·5	0·0
Contradicting evidence is poorly documented.	63·8	36·2	0·0
Conclusions do not adequately reflect the findings as presented in the results section.	46·6	51·7	1·7
Possible impact of the limitations on the results is not or poorly discussed.	44·0	56·0	0·0
Conclusions are not supported by the results as presented in the context of the referenced literature.	43·1	54·3	2·6
The conclusions do not adequately reflect the objectives of the study.	35·3	61·2	3·4
Supporting evidence is poorly documented.	31·9	68·1	0·0
Sources. direction and magnitude of bias are not or poorly discussed. or just listed without further discussion.	27·6	72·4	0·0
The conclusions in the abstract do not adequately reflect the conclusions in the main text.	22·4	75·0	2·6
The main results discussed in the discussion paragraph do not adequately address the original objectives/research questions as posed in the introduction.	20·7	75·9	3·4
The outcome measure used does not allow the conclusions that are stated. *	18·1	81·9	0·0
Lack of distinction between results and discussion. The results section contains elements of discussion and interpretation beyond the scope of explaining the results.	17·2	82·8	0·0
The sampling methodology does not allow the type of generalization provided.	15·5	84·5	0·0
The objectives/research questions of the study are differently phrased in the introduction and the discussion.	14·7	36·2	49·1
The order of presenting the results in de discussion is inconsistent with the ordering of the objectives/research questions as posed in the introduction.	14·7	75·0	10·3
Hyperboles and exaggerating adjectives are unjustifiably used	12·1	87·9	0·0
The title does not adequately reflect the main findings.	11·2	88·8	0·0
The abstract does not adequately reflect the main findings.	10·3	89·7	0·0
A potential causal relationship claimed in the discussion paragraph is not justified.	10·3	89·7	0·0
The outcome measure does not adequately reflect the objectives/research questions of the study. *	9·6	90·4	0·0
A causal relationship is claimed. although the research design is not appropriate to determine causation.	9·6	90·4	0·0
The relevance of statistically significant results with small effect size is overstated. *	9·6	90·4	0·0
Generalising findings to settings/institutions not included in the original study is not justified.	9·5	89·7	1·0
The conclusion/discussion distracts from main outcomes by overstating the relevance of secondary outcomes. *	8·4	91·6	0·0

For peer review only

Non-significant results are discussed without addressing significance.	8·4	91·6	0·0
Generalising findings to geographical locations not included in the original study is not justified.	6·0	94·0	0·0
Evidence is used inappropriately to support the findings.	5·2	94·9	0·0
A causal relationship is claimed although potential sources of bias and their potential impact on the findings were not discussed. *	3·6	96·4	0·0
Jargon. technical and complex language. that does not fit the journal audience. are used without properly explaining the meaning.	3·4	96·6	0·0
The main source of evidence for supporting the results is based on the same underlying data.	2·6	96·6	0·9
Generalising findings to populations not included in the original sample is not justified.	2·6	97·4	0·0
Causative wording is used in the hypothesis/research question, although there is no theory to support causation. *	2·4	97·6	0·0
Possible clinical relevance of statistically non-significant results is not addressed. *	2·4	97·6	0·0
Generalising findings to time periods not included in the original study is not justified.	0·0	100·0	0·0

** QRPs only applicable to quantitative research-based publications (n=83)*
*** 50 ·0% of publications did not mention implications for policy or practice.*
**** 34·5% of publications did not mention recommendations for policy or practice.*

For peer review only

STROBE Statement—checklist of items that should be included in reports of observational studies

	Item No	Recommendation	
**Title and abstract**	1	(*a*) Indicate the study's design with a commonly used term in the title or the abstract	No
		(*b*) Provide in the abstract an informative and balanced summary of what was done and what was found	Page 2
**Introduction**			
Background/rationale	2	Explain the scientific background and rationale for the investigation being reported	Page 3
Objectives	3	State specific objectives, including any prespecified hypotheses	Page 3
**Methods**			
Study design	4	Present key elements of study design early in the paper	Page 4
Setting	5	Describe the setting, locations, and relevant dates, including periods of recruitment, exposure, follow-up, and data collection	Page 4
Participants	6	(*a*) *Cohort study*—Give the eligibility criteria, and the sources and methods of selection of participants. Describe methods of follow-up<br>*Case-control study*—Give the eligibility criteria, and the sources and methods of case ascertainment and control selection. Give the rationale for the choice of cases and controls<br>*Cross-sectional study*—Give the eligibility criteria, and the sources and methods of selection of participants	Page 4
		(*b*) *Cohort study*—For matched studies, give matching criteria and number of exposed and unexposed<br>*Case-control study*—For matched studies, give matching criteria and the number of controls per case	n.a.
Variables	7	Clearly define all outcomes, exposures, predictors, potential confounders, and effect modifiers. Give diagnostic criteria, if applicable	Page 5
Data sources/ measurement	8*	For each variable of interest, give sources of data and details of methods of assessment (measurement). Describe comparability of assessment methods if there is more than one group	*Page 4-5*
Bias	9	Describe any efforts to address potential sources of bias	Page 4<br>Page 5
Study size	10	Explain how the study size was arrived at	Page 4
Quantitative variables	11	Explain how quantitative variables were handled in the analyses. If applicable, describe which groupings were chosen and why	Page 5
Statistical methods	12	(*a*) Describe all statistical methods, including those used to control for confounding	Page 5
		(*b*) Describe any methods used to examine subgroups	n.a.

For peer review only - http://bmjopen.bmj.com/site/about/guidelines.xhtml

and interactions	
(*c*) Explain how missing data were addressed	No missing data.
(*d*) *Cohort study*—If applicable, explain how loss to follow-up was addressed<br>*Case-control study*—If applicable, explain how matching of cases and controls was addressed<br>*Cross-sectional study*—If applicable, describe analytical methods taking account of sampling strategy	n.a.
(*e*) Describe any sensitivity analyses	n.a.

Continued on next page

For peer review only

**Results**			
Participants	13*	(a) Report numbers of individuals at each stage of study—eg numbers potentially eligible, examined for eligibility, confirmed eligible, included in the study, completing follow-up, and analysed	n.a.
		(b) Give reasons for non-participation at each stage	n.a.
		(c) Consider use of a flow diagram	n.a.
Descriptive data	14*	(a) Give characteristics of study participants (eg demographic, clinical, social) and information on exposures and potential confounders	Page 6
		(b) Indicate number of participants with missing data for each variable of interest	n.a.
		(c) *Cohort study*—Summarise follow-up time (eg, average and total amount)	n.a.
Outcome data	15*	*Cohort study*—Report numbers of outcome events or summary measures over time	n.a.
		Case-control study—Report numbers in each exposure category, or summary measures of exposure	
		Cross-sectional study—Report numbers of outcome events or summary measures	
Main results	16	(*a*) Give unadjusted estimates and, if applicable, confounder-adjusted estimates and their precision (eg, 95% confidence interval). Make clear which confounders were adjusted for and why they were included	Page 8
		(*b*) Report category boundaries when continuous variables were categorized	n.a.
		(*c*) If relevant, consider translating estimates of relative risk into absolute risk for a meaningful time period	n.a.
Other analyses	17	Report other analyses done—eg analyses of subgroups and interactions, and sensitivity analyses	n.a.
**Discussion**			
Key results	18	Summarise key results with reference to study objectives	Page 9
Limitations	19	Discuss limitations of the study, taking into account sources of potential bias or imprecision. Discuss both direction and magnitude of any potential bias	Page 9
Interpretation	20	Give a cautious overall interpretation of results considering objectives, limitations, multiplicity of analyses, results from similar studies, and other relevant evidence	Page 10-11
Generalisability	21	Discuss the generalisability (external validity) of the study results	Page 9
**Other information**			
Funding	22	Give the source of funding and the role of the funders for the present study and, if applicable, for the original study on which the present article is based	Page 11

*Give information separately for cases and controls in case-control studies and, if applicable, for exposed and unexposed groups in cohort and cross-sectional studies.

For peer review only

Note: An Explanation and Elaboration article discusses each checklist item and gives methodological background and published examples of transparent reporting. The STROBE checklist is best used in conjunction with this article (freely available on the Web sites of PLoS Medicine at http://www.plosmedicine.org/, Annals of Internal Medicine at http://www.annals.org/, and Epidemiology at http://www.epidem.com/). Information on the STROBE Initiative is available at www.strobe-statement.org.

For peer review only

For peer review only - http://bmjopen.bmj.com/site/about/guidelines.xhtml

BMJ Open

The occurrence and nature of questionable research practices in the reporting of messages and conclusions in international scientific Health Services Research publications: A structured assessment of publications authored by researchers in the Netherlands

Journal:	*BMJ Open*
Manuscript ID	bmjopen-2018-027903.R2
Article Type:	Research
Date Submitted by the Author:	28-Feb-2019
Complete List of Authors:	Gerrits, Reinie; Amsterdam UMC, University of Amsterdam, Amsterdam Public Health research institute, Public Health<br>Jansen, Tessa; Amsterdam UMC, University of Amsterdam, Amsterdam Public Health research institute, Public Health<br>Mulyanto, Joko; Amsterdam UMC, University of Amsterdam, Amsterdam Public Health research institute, Public Health<br>van den Berg, Michael; Amsterdam UMC, University of Amsterdam, Amsterdam Public Health research institute, Public Health<br>Klazinga, Niek; Amsterdam UMC, University of Amsterdam, Amsterdam Public Health research institute, Public Health<br>Kringos, Dionne; Amsterdam UMC, University of Amsterdam, Amsterdam Public Health research institute, Public Health
**Primary Subject Heading**:	Health services research
Secondary Subject Heading:	Evidence based practice, Research methods, Health policy
Keywords:	Health Services Research, Responsible Research Practices, Questionable Research Practices, Scientific Reporting

SCHOLARONE™
Manuscripts

The occurrence and nature of questionable research practices in the reporting of messages and conclusions in international scientific Health Services Research publications: A structured assessment of publications authored by researchers in the Netherlands

Reinie G. Gerrits1, MSc, Tessa Jansen1 MSc, Joko Mulyanto1 MD, Michael J. van den Berg1 PhD, Niek S. Klazinga1 PhD, Dionne S. Kringos1 PhD.

1 Amsterdam UMC, University of Amsterdam, Department of Public Health, Amsterdam Public Health research institute, P.O. Box 22660, 1100 DD Amsterdam, The Netherlands

Corresponding author
Reinie G. Gerrits
Address: P.O. Box 22660, 1100 DD Amsterdam, the Netherlands
Telephone: +31683555977
E-mail: r.g.gerrits@amc.uva.nl

For peer review only - http://bmjopen.bmj.com/site/about/guidelines.xhtml

Abstract

Objectives: Explore the occurrence and nature of questionable research practices (QRPs) in the reporting of messages and conclusions in international scientific HSR publications authored by researchers from health services research (HSR) institutions in the Netherlands.
Design: In a joint effort to assure the overall quality of HSR publications in the Netherlands, thirteen HSR institutions in the Netherlands participated in this study. Together with these institutions, we constructed and validated an assessment instrument covering 35 possible QRPs in the reporting of messages and conclusions. A QRP in the reporting of messages and conclusions in HSR is defined as *"to report, either intentionally or unintentionally, conclusions or messages that may lead to incorrect inferences and do not accurately reflect the objectives, the methodology or the results of the study."* Two reviewers independently assessed a random sample of 116 HSR articles authored by researchers from these institutions published in international peer-reviewed scientific journals in 2016.
Setting: Netherlands, 2016.
Sample: 116 international peer-reviewed HSR publications.
Main outcome measures: Median number of QRPs per publication, the percentage of publications with observed QRP frequencies, occurrence of specific QRPs, and difference in total number of QRPs by methodological approach, type of research, and study design.
Results: We identified a median of six QRPs per publication, out of 35 possible QRPs. QRPs occurred most frequently in the reporting of implications for practice, recommendations for practice, contradictory evidence, study limitations, and conclusions based on the results and in the context of the literature. We identified no differences in total number of QRPs in papers based on different methodological approach, type of research or study design.
Conclusions Given the applied nature of HSR, both the severity of the identified QRPs, and the recommendations for policy and practice in HSR publications warrant discussion. We recommend that the HSR field further define and establish its own scientific norms in publication practices to improve scientific reporting and strengthen the impact of HSR. The results of our study can serve as an empirical basis for continuous critical reflection on the reporting of messages and conclusions.

Funding: ZonMw grant number 445001003.

Strengths and limitations of the study

- Given the explorative nature of this study we applied a broad and sensitive definition of 'questionable research practices' (QRPs), that allows for the identification of QRPs previously overlooked in related assessments.
- This study describes an assessment of publications and is therefore able to detect QRPs that go unnoticed in survey studies that rely on self-report.
- Although we aimed to develop a reliable measurement instrument that would guide the review process, the instrument allowed latitude for the reviewer's interpretation.
- In our assessment method, we relied on consensus among assessors, which inevitably introduces some subjectivity.
- Because publications were selected based on the title, selection bias might have occurred.

For peer review only - http://bmjopen.bmj.com/site/about/guidelines.xhtml

Introduction

In 2009, Chalmers and Glasziou estimated that 85% of research funding in biomedical sciences was wasted avoidably,[1] resulting in *The Lancet*'s series "Increasing value: reducing waste". This series has stirred the international scientific community, prompting funders, regulators, academic institutions, and scientific publishers to act. Funders of biomedical research have responded by organising conferences on research waste, and journal editors have initiated discussions on data sharing and open access.[2] While evidence for questionable research practices (QRPs) in biomedical sciences is mounting,[1] little is known about the occurrence and nature of QRPs in the policy- and management-oriented field of health services research (HSR). In particular, QRPs in the reporting of messages and conclusions have flown under the radar. The term 'questionable research practices' is commonly used to describe practices such as selective publication of results, concealing of conflicts of interests, and describing a hypothesis after finding significant results.[3] A questionable practice is not necessarily wrongful, but does 'raise questions'. In this study we further define the meaning of questionable research practices in the reporting of messages and conclusions in the field of HSR specifically.

The HSR field is an applied field of research, and produces evidence on topics such as co-payments, evaluation of quality improvement efforts, cost-effectiveness of medications, patient empowerment, therapy compliance, and effects of policies. Given the growing evidence for the prevalence of QRPs in the reporting of messages and conclusions in the biomedical field,[4,5] QRPs may also occur in the HSR field. In the biomedical field, a systematic review by Chiu et al. (2017) shows that estimates for the occurrence of questionable research practices in the interpretation of results in scientific publications vary from 10% of publications deriving discordant conclusions from study results to 100% of publications containing rhetorical practices resulting in spin, such as failure to compare risk to benefits in randomized controlled trials.[4]

Just like biomedical researchers, health services researchers are under pressure to publish in high-impact journals to increase their citation scores and attract media attention to augment their prestige and chances for future research funding and job security.[6-9] Unlike biomedical research, HSR findings are not easily generalised from one local or national health services setting to another, and messages and conclusions tend to be limited to a specific national context.[10] A broad spectrum of quantitative and qualitative methods is used in HSR, including designs that are less subject to strict codes of execution than randomized controlled trials, such as observational and case study designs. Furthermore, HSR has difficulty creating alignment between the construction of scientific knowledge and the implementation of that knowledge in policy and practice.[11] This combination of HSR specific characteristics may result in a different set of QRPs in the reporting of a scientific study. The variation of designs other than RCTs, as is more common in the biomedical field, might invite unjustified claims of causality. Moreover, the context specific research may increase unjustified claims of generalisability, and the difficulty in translating knowledge to practice may result in unsupported recommendations or implications.

Although reporting in scientific publications is highly standardised, the discussion and conclusion sections offer researchers relative freedom when deriving messages and conclusions from study results.[5] We explored the occurrence and nature of QRPs in the reporting of messages and conclusions in international scientific HSR publications authored by researchers from HSR institutions in the Netherlands. We also examined the relationship between study type, methodology, and design and the occurrence of QRPs. With our study, we want to fuel the debate on fostering responsible messages and conclusions, and provide a basis for the discussion on QRPs in the international HSR field.

Methods

Setting

This study assessed scientific publications authored by researchers from 13 HSR groups, departments, or institutions (hereafter referred to as "HSR institutions") in the Netherlands, including both academic and non-academic institutions. These institutions all agreed to participate in an effort to assure the overall quality of HSR publications in the Netherlands.

Defining QRPs in the reporting of messages and conclusions in HSR

We conducted a literature review on QRPs in the reporting of messages and conclusions in biomedical research and HSR.[12-14] An initial definition of QRPs in the reporting of messages and conclusions in HSR was proposed and discussed at a consensus meeting with the directors/leaders of the 13 participating institutions. This was then validated through inputs from five leading international health services researchers (10 were invited; 50% non-response), and resulted in the following amended definition:

For peer review only - http://bmjopen.bmj.com/site/about/guidelines.xhtml

"To report, either intentionally or unintentionally, conclusions or messages that may lead to incorrect inferences and do not accurately reflect the objectives, the methodology or the results of the study."

Measurement instrument

We developed an extensive list of QRPs in the reporting of messages and conclusions. Items were based on the EQUATOR checklists[15] and earlier checklists for identifying "spin" (ie, "a way to distort science reporting without actually lying")[5] or other QRPs.[13,14,16,17] The proposed list of QRPs was reviewed, refined, and complemented using 14 semi-structured interviews with the directors/leaders and representatives (n=19) of the 13 participating HSR institutions. Next, the five participating international health services researchers provided email feedback on the list resulting from these interviews; the list was adapted accordingly, resulting in 35 possible QRPs in the reporting of messages and conclusions in HSR publications.

We developed a data extraction form in Excel that contained the list of QRPs and bibliometric information, and conducted a pilot to evaluate its feasibility and usability. In the pilot, two assessors (RG, TJ) independently assessed five international HSR publications to identify modifications needed to improve the form, and to align the interpretation of the items. The project group discussed the proposed modifications, resulting in the final version. The data extraction form, (supplementary material 1) and a methodology of the development of the data extraction form (supplementary material 2) is provided in the supplementary material.

Sample

We aimed to include ten HSR publications from each participating HSR institution. Inclusion criteria were: published in 2016 in an international peer-reviewed scientific journal, written in English, reporting HSR findings, and first- and/or last-authored by researchers affiliated with the respective HSR institution. As both the first author and the research institution are likely important factors influencing the occurrence of QRPs, only unique first authors were included in the publication. Moreover, not more than 10 publications per institution were included. This will ensure a maximum spread of authors and institutions across the sample.

Publication lists of the HSR institutions were retrieved either by searching publicly accessible online sources (eg, annual reports, open repositories or the research groups' website) or obtained from secretaries or librarians. All lists were verified by the respective HSR institutions. These lists included both HSR and non-HSR publications.

Two researchers (RG, TJ) selected all titles from the 13 publication lists that were likely to indicate empirical or systematic assessment studies in HSR. Publications were included if their title fitted the definitions of HSR by Juttmann (2007)[18] and Lohr & Steinwachs (2002).[19] These definitions are commonly used by HSR institutions (eg, in education) in the Netherlands. To select HSR studies, TJ and RG first individually selected titles from the publication lists. Next, RG and TJ compared their selections of titles and noted any differences. After completing the selection of the first HSR publications, selection was reviewed and approved by the research group (NK, DK, MB). TJ and RG then continued applying the selection method to the remaining publication lists. In a consensus meeting between TJ and RG, differences in selected titles were resolved by discussing its fit with the definition. Consensus was reached on all included publications.

The HSR publications (n=717) were assigned a random number. Per institution, the publications with unique first authors with the lowest assigned number were included in the sample. Three HSR institutions did not have enough publications with unique first authors, resulting in a selection of nine, eight, and two publications for these institutions. Furthermore, two publications were excluded during assessment because they concerned research protocols. These publications were replaced by another publication authored by the same institution. One publication was excluded because its methodology was considered incomprehensible by the reviewers. Ultimately, 116 HSR publications were included (16% of tot sample).

Assessment process

Two reviewers independently assessed all publications (RG and TJ or RG and JM). RG has primarily qualitative HSR experience and is trained in health economics. TJ and JM have primarily quantitative HSR experience and are trained in public health, management, economics, and law; and medicine, respectively.

The assessment started with a test phase. During this phase, agreements and disagreements in assessments of the first 30 publications were thoroughly discussed (by RG, TJ, NK, and DK) to increase the accuracy of the assessments; agreement between the two reviewers (TJ, RG) was 81% for the first 20 publications, which increased to 82% when assessing the next 10 publications. The notion emerged that it was necessary having two reviewers with complementary expertise assess each publication independently, followed by a consensus procedure and random check by the project leaders. RG trained the third reviewer (JM).

For peer review only - http://bmjopen.bmj.com/site/about/guidelines.xhtml

RG assessed all included publications, while TJ assessed the first 59 publications, and JM the remaining 57. All data were entered in the data extraction form. QRPs were coded as either 1, "present"; 0, "not present"; -8, "not applicable to this study" (primarily used for items not applicable for qualitative research); or -9, "not assessable". To justify their assessments, the reviewers recorded their motivation for every identified QRP. At a later stage, QRPs in implications and recommendations for policy and practice were further refined into "not mentioned" if no implication or recommendation was included in the publications, and "not sufficiently justified", if the authors did not provide any explanation for their implications or recommendations. The reviewers held regular consensus meetings (after review of 10 publications) to discuss and reach agreement on all identified QRPs.

During the consensus meetings, the reviewers compared their assessment of all items. Inconsistencies between the individually assessed QRPs were identified, discussed and adapted. Any remaining disagreements (n=2) were resolved by a senior researcher (DK). NK and DK each reassessed a random sample of six publications, so 10% of all included publications (n=12). As a result, two identified QRPs were retracted, and two QRPs were added to the reassessed publications.

Analysis

The characteristics of the included publications were described by calculating their occurrence with the percentage or mean number of publications.

We counted the total number of QRPs per publication, and the percentage of HSR publications with number of observed QRPs. The latter was visualised in a histogram. Occurrence of specific QRPs was calculated as a percentage of publications containing this particular QRP. The percentage of publications containing QRPs that were not applicable to qualitative research was calculated only for quantitative and mixed-methods-based publications (n = 83), (eg. the QRP: "The relevance of statistically significant results with small effect size is overstated" is only applicable to quantitative research).

We used a Kruskal-Wallis test to calculate the difference in total number of QRPs applicable to all research designs by methodological approach (quantitative, qualitative, and mixed), type of research (descriptive, exploratory, hypothesis testing, and measurement instruments), and study design (observational, (quasi) experimental, systematic review, economic evaluation, case study, and meta-analyses). We used the STROBE checklist for observational studies in the reporting of this research.[20] Analyses were conducted using SPSS version 24.[21]

Patient and Public Involvement

No patients were involved in this study. This study was designed with the input provided by the participating HSR institutions at a consensus meeting at the onset of the study, and individual interviews with the directors/leaders of the 13 participating institutions. During a progress meeting with the participating institutions, preliminary (aggregated level) results were discussed to validate and complement the interpretation of findings.

Ethics approval

A waiver for ethical approval was obtained for this study from the medical ethics review committee at Amsterdam UMC. To avoid negative consequences for the authors of the included publications, each publication was assigned a unique identification number. Extracted data were entered in SPSS using this number to separate author information from the study data.

For peer review only

For peer review only - http://bmjopen.bmj.com/site/about/guidelines.xhtml

Results

Characteristics of included publications

Table 1 presents the characteristics of the 116 included publications from the 13 participating HSR institutions. To summarise, 54.3% of the publications were quantitative, 28.4% were qualitative, and 17.2% applied a mixed-methods approach. Sixteen percent of the publications were based on a published study protocol. The mean impact factor of the journals was 2.81, and the average number of authors was six.

Table 1: Characteristics of included publications

**Total (N= 116)**		**n (%)**
HSR domain	Policy	19 (16·4)
	Social factors	11 (9·5)
	Financing Systems	10 (8·6)
	Organizational structures & processes	43 (37·1)
	Health technologies	11 (9·5)
	Personal Behaviours	22 (19·0)
Methodological approach	Quantitative	63 (54·3)
	Qualitative	33 (28·4)
	Mixed methods	20 (17·2)
Type of research	Descriptive	31 (26·7)
	Exploratory	59 (50·9)
	Hypothesis testing	19 (16·4)
	Measurement instruments	5 (4·3)
	Other	2 (1·7)
Design	Observational	59 (50·9)
	(Quasi) experimental	9 (7·8)
	Systematic review	17 (14·7)
	Economic evaluation	5 (4·3)
	Meta analyses	3 (2·6)
	Case study	22 (19·0)
	Other	1 (0·9)
Protocol published		19 (16·4)
Funder of study stated		98 (84·5)
Contributions stated		57 (49·1)
Number of included journals		80 (100·0)
		Mean
Impact factor journal (n=93 publications*)		2·81 (SD 1·45)
Number of authors (n=116)		6·12 (SD 5·53)

**Not all journals had an impact factor. Mean impact factor was calculated over 93 publications.*

For peer review only

Occurrence of QRPs per publication

Of the 116 HSR publications, the median number of QRPs per publication was six (interquartile range, 5·75), out of 35 possible QRPs. The distribution of the observed frequency of QRPs across publications is visualised in figure 1.

Figure 1: Percentage of HSR publications with number of observed QRPs in the reporting of messages and conclusions

Frequency of QRPs per type

For each of the QRPs, we counted how often they were identified in the included publications. Supplementary material 3, table 1 presents the percentage of occurrence per QRP type.

QRPs that occurred most frequently were:

- Implications for policy and practice do not adequately reflect the results in the context of the referenced literature (69·0%)*;

For peer review only - http://bmjopen.bmj.com/site/about/guidelines.xhtml

*In 50·0% of publications, no implications for policy and practice were mentioned, and in 19·0% of publications, implications were mentioned without adequate justification.

- Recommendations for policy and practice do not adequately reflect the results in the context of the referenced literature (65·5%)**;

  **In 34·5% of publications, no recommendations for policy and practice were reported, and in 31·0% of publications, recommendations were mentioned without adequate justification.

- Contradicting evidence is poorly documented (63·8%);
- Conclusions do not adequately reflect the findings as presented in the results section (46·6%);
- Possible impact of the limitations on the results is not or poorly discussed (44·0%);
- Conclusions are not supported by the results as presented in the context of the referenced literature (43·1%).

QRPs that occurred least frequently were:

- The main source of evidence for supporting the results is based on the same underlying data (2·6%);
- Generalising findings to populations not included in the original sample is not justified (2·6%);
- Causative wording is used in the hypothesis/research question, although there is no theory to support causation (2·4%);
- Possible clinical relevance of statistically non-significant results is not addressed (2·4%);
- Generalising findings to time periods not included in the original study is not justified (0·0%).

Distribution of QRPs

Figure 2 shows the distribution of QRPs across publications. The horizontal axis shows the publications (n=116) ordered from the publication with the lowest (0) to the highest number (18) of observed QRPs in the reporting of messages and conclusions. The vertical axis shows the QRPs ordered from least (Generalisation to different time period) to most (Implications for practice are lacking) frequently observed. On the right vertical axis, the occurrence of QRPs is presented in number of QRPs counted. Each dot represents a QRP.

Figure 2. Distribution of QRPs in the reporting of messages and conclusions across HSR publications, ordered from lowest to highest number of observed QRPs.

The difference in the number of QRPs by publication characteristics

Table 2 shows the associations between total number of QRPs (applicable to all study designs) and methodological approach (quantitative, qualitative, and mixed), type of research (descriptive, exploratory, hypothesis testing, and measurement instruments), and study design (observational, (quasi) experimental, systematic review, economic evaluation, case study, and meta-analyses).
No statistically significant differences in number of QRPs was found by type of research, methodological approach, or study design.

Table 2. Association between total number of QRPs and type of research, methodological approach, and study design

	Median	95% CI	p-value
Methodological approach			0·339
Quantitative	5	4·88 – 6·43	
Qualitative	6	4·98– 7·62	
Mixed methods	7	5·34 – 8·46	
Type of research			0·295
Descriptive	6	4·77 – 6·78	
Exploratory	7	5·76 – 7·60	
Hypothesis testing	4	3·40 – 6·81	
Measurement instruments	5	2·14 – 6·66	
Other	5	-33·12 – 43·12	
Study design			0·159
Observational	6	5·56 – 7·21	
(Quasi) experimental	3	2·07 – 5·71	
Systematic review	6	4·61 – 8·33	
Economic evaluation	4	1·61 – 7·59	
Case studies	6	4·71 – 8·01	
Meta-analyses	5	0·50 – 10·84	

For peer review only - http://bmjopen.bmj.com/site/about/guidelines.xhtml

Discussion

We explored the occurrence and nature of QRPs in the reporting of messages and conclusions in international scientific HSR publications authored by researchers from HSR institutions in the Netherlands, and examined the relationship between study type, methodology, and design and the occurrence of QRPs. Our results indicate that HSR publications have a median of six QRPs per publication. We identified most QRPs in the reporting of implications for policy and practice, recommendations for policy and practice, contradictory evidence, study limitations, and conclusions as based on the results and in the context of the literature. No significant associations between number of QRPs and type of study, study design, or methodological approach were identified.

Limitations and Strengths

We applied a broad and sensitive definition of 'questionable', for instance by considering the absence of contradictory evidence or the absence of implications and recommendations for policy and practice as a QRP. The choice to not present contradictory evidence does not defy current publication checklists, yet this practice may hinder interpretation of findings in the full context of evidence. If authors searched for contradictory evidence, but did not mention its absence, readers of the publication would not have any clues on its existence. Knowledge on the occurrence of QRPs is often derived from survey studies, relying on self-report.3 These studies focus on the knowledge of consciously conducted, well-known QRPs. Our assessment approach allowed us to gain insight in less severe, more likely unconsciously occurring QRPs in the reporting of messages and conclusions specifically. The number of QRPs identified through assessment is generally higher than in studies relying on self-report.[3,4] With our broad definition encompassing 35 possible QRPs we bring to light the areas that offer possibilities for further enhancing publication practices in HSR. Consequently, this definition allows for a discussion in the field of HSR on the extent to which the identified QRPs are acceptable. This is an important strength of our applied approach.

Although we endeavoured to develop a reliable measurement instrument that would guide the review process, the instrument allowed latitude for the reviewer's interpretation. Consequently, a different group of reviewers might arrive at somewhat different scoring frequencies for observed QRPs. However, because we defined each QRP in detail, it is unlikely that there would be substantial differences in the overall distribution of different types of QRPs across publications. Our consensus method contains a degree of subjectivity, and there is the risk that one reviewer's opinion will dominate. To counteract this, NK and DK performed random checks on 10% of all assessments. By recording the motivation for every identified QRP, we supported the consistency of our measurement and justified our results. Because publications were selected based on the title, selection bias might have occurred. Considering we found no relationship between study characteristics and number of QRPs, it is unlikely that a different sample would have led to different results. Inevitably, reviewers sometimes assessed publications written by authors they knew professionally or personally, and as such, a positive view of a colleague's work might have led to underestimating the QRPs in these publications.

Our study results may be representative for HSR research publications internationally. Given the fact that publication in international journals is highly standardised in terms of language (English) and format, our findings can most likely be transferred to HSR communities in other countries.

Interpretation

In HSR publications, recommendations for policy and practice warrant most attention. A study can be conducted properly, using a sound design and appropriate methodology. However, making recommendations without adequate justification could lead to incorrect inferences in policy and the management of healthcare, and undermine society's confidence in science. [11,22-25]

Measures for safeguarding scientific soundness like those often used in biomedical research (eg, trial registration, open data policies, and an improved reporting and archiving infrastructure [26]) do not address reporting conclusions not supported by study results, and are not tailored to the observational and explorative designs most prevalent in HSR. Moreover, existing publication checklists address a report's completeness, but do not question the justification of the conclusions.[5] If we intend to improve the reporting of HSR conclusions and recommendations, we will need to better understand the factors that influence authors when reporting the discussion and conclusions section of a HSR publication eg, media pressure and relationships with funders.[6,7,9,27] Journals may have influence on the reporting of a study through control of the review process.[28] Moreover, research institutions may prevent the occurrence of QRPs by enhancing internal integrity, training in scientific writing and communication amongst researchers.[29] Consequently, subsequent research can focus on what

For peer review only - http://bmjopen.bmj.com/site/about/guidelines.xhtml

influences researchers when writing their scientific publications, and what factors play a role in the process from research design to the acceptance of a manuscript by a peer-reviewed journal.

A third of the HSR publications studied gave no recommendations for policy or practice, while another third did not provide an adequate justification for the recommendations. One could argue that HSR is an applied field of research, and that its ultimate goal should be to contribute to better health services and systems; researchers should therefore take responsibility for providing guidance to those who can act on the research findings instead of leaving them empty-handed. On the other hand, health services researchers may feel more comfortable committing to a more traditional interpretation of the role of academics, refraining from normative judgement. If the latter is the dominant viewpoint, the HSR community needs to consider the role of scientific evidence in helping decision makers address the challenges they face, and informing policies and practices. Internationally, the HSR community has been promoting further strengthening of the link between HSR and practice.[30]

In biomedical research, research being "new" might contribute to a confused assessment of implications.[31] This problem is amplified in HSR, where there is a limited accumulation of evidence. HSR considers a larger range of contextual factors and stakeholders in politics or management. Moreover, HSR recommendations are often based on observational or exploratory research, which is considered to be weak evidence in biomedical circles (eg, the GRADE checklist).[32] Perhaps the norms determined by the biomedical research field make health services researchers hesitant to provide any implications or recommendations at all.

Implications and recommendations for policy and practice

The HSR field currently seems to adhere to the norms and expectations set by the biomedical field, even though HSR is multidisciplinary, and differences in approach and type of methodology pose serious challenges to observing these norms. Therefore, the HSR community needs to further define specific scientific norms appropriate to the field.

Scientific norms are developed through the forum of a scientific community.[33] This forum function is particularly strong in the Netherlands, where a community of HSR institutions work together closely. Our study was able to bring together the main Dutch academic and non-academic HSR institutions. Consequently, the results of our study help to facilitate critical reflection on the current state of research and encourage debate on how to systematically advance the reporting of messages and conclusions in HSR. Such a debate in the Dutch context is needed, given the attempts over the past decade by the Netherlands Organisation for Health Research and Development (ZonMw) to strengthen the link between research and practice. It would also be very timely, considering the ongoing, overarching Dutch research programme on responsible research practices funded by ZonMw, of which this study is a part. We recommend the HSR community to reflect on the questions our results bring forward: how do we include implications and recommendations for policy and practice in scientific publications?; how should we describe conclusions in context of literature with limited accumulation of evidence?; and what is the severity of the identified QRPs? Through this publication, we would like to urge journal editors and those working in the international field of HSR to join in this debate. After establishing norms regarding these frequently occurring QRPs, journal editors and HSR institutions may contribute to the prevention of QRPs by implementing strategies tailored to HSR research specifically.

Conclusions

QRPs in the reporting of messages and conclusions occur frequently in peer-reviewed international scientific HSR publications from Dutch HSR institutions. These QRPs differ in severity and cannot always be qualified as wrongful, but they do 'raise questions'. To ensure the applicability of HSR research in policy and practice, the HSR field should reflect on scientific norms for the reporting of conclusions and the inclusion of recommendations for policy and practice. Our study can serve as an empirical basis for continuous critical reflection on the current state of research, and encourage debate on how to systematically advance the reporting of messages and conclusions in HSR.

For peer review only - http://bmjopen.bmj.com/site/about/guidelines.xhtml

Acknowledgements

We thank the Dutch HSR institutions that participated in this study: Erasmus MC, department of Public Health; Erasmus University, Erasmus School of Health Policy and Management; Leiden University Medical Centre, department of Medical Decision making and the department of Public Health and Primary Care; University Maastricht, Health Services Research group; the Netherlands Institute for Health Services Research (NIVEL); Radboud UMC, IQ healthcare; the National Institute for Public Health and the Environment (RIVM); University of Groningen, faculty of Economics and Business; Tilburg University, Social and Behavioural Sciences, Tranzo; Trimbos institute; University Medical Centre Utrecht, Julius Centre for Health Sciences and Primary Care; Amsterdam UMC – Vrije Universiteit Amsterdam, and the Amsterdam UMC - University of Amsterdam. Furthermore, we also would like to thank project advisers Anton Kunst, Thomas Plochg, and Patrick Romano. We thank Ellen Nolte, Enrique Bernal-Delgado, Michel Wensing, Oliver Groene, and Wilm Quentin for their valuable feedback on the definition of QRPs and the measurement instrument.

Role of funding source

This study was funded by ZonMw grant number 445001003. The funder had no role in the study design, in the collection, analysis, and interpretation of data, in the writing of the manuscript, and in the decision to submit the paper for publication. All authors had full access to the data during the conduct of this study and take responsibility for the integrity of the data and the data analysis.

Competing interests

None declared.

Contribution of authors

DK, NS, MB, RG and TJ designed the study. RG, TJ, and JM collected the data, and RG and JM analysed the data and RG drafted the manuscript. TJ, JM, MB, NS and DK were all involved in the interpretation of the results and were major contributors to the writing of the manuscript. All authors read and approved the final manuscript and are accountable for all aspects of the work.

Transparency statement

The lead author (DK) affirms that the manuscript is an honest, accurate, and transparent account of the study being reported; that no important aspects of the study have been omitted; and that any discrepancies from the study as originally planned have been explained.

Data sharing statement

The data concern the quantitative results from a review of scientific publications. Anonymized data is available till May 1st, 2024, by contacting d.s.kringos@amc.uva.nl.

Licence

The Corresponding Author has the right to grant on behalf of all authors and does grant on behalf of all authors, an exclusive licence (or non-exclusive licence for UK Crown and US Federal Government employees) on a worldwide basis to the BMJ Publishing Group Ltd, and its Licensees to permit this article (if accepted) to be published in the BMJ and any other BMJPGL products and to exploit all subsidiary rights, as set out in our licence.

For peer review only

References

1. Chalmers I, Glasziou P. Avoidable waste in the production and reporting of research evidence. *The Lancet* 2009; 374(9683): 86-9.

2. Moher D, Glasziou P, Chalmers I, et al. Increasing value and reducing waste in biomedical research: who's listening? *The Lancet* 2016; 387(10027): 1573-86.

3. Fanelli D. How Many Scientists Fabricate and Falsify Research? A Systematic Review and Meta-Analysis of Survey Data. *PLOS ONE 2009*; 4(5): e5738.

4. Chiu K, Grundy Q, Bero L. 'Spin' in published biomedical literature: A methodological systematic review. *PLoS Biol* 2017; 15(9): e2002173.

5. Boutron I, Ravaud P. Misrepresentation and distortion of research in biomedical literature. *Proc Natl Acad Sci USA* 2018; 115(11): 2613-9.

6. Ioannidis JPA, Greenland S, Hlatky MA, et al. Increasing value and reducing waste in research design, conduct, and analysis. *The Lancet* 2014; 383(9912): 166-75.

7. Weingart P. Science and the media. *Res Policy* 1998; 27(8): 869-79.

8. Tijdink JK, Schipper K, Bouter LM, Maclaine Pont P, de Jonge J, Smulders YM. How do scientists perceive the current publication culture? A qualitative focus group interview study among Dutch biomedical researchers. *BMJ Open* 2016; 6(2): e008681.

9. Bouter LM. Commentary: Perverse Incentives or Rotten Apples? *Accountability in Research* 2015; 22(3): 148-61.

10. Porter ME. What Is Value in Health Care? *New England Journal of Medicine* 2010; 363(26): 2477-81.

11. Groen TP. Startdocument 'Systeemfalen' The Hague, 2013. Dutch

12. Horton R. The rhetoric of research. *BMJ* 1995; 310(6985): 985.

13. Boutron I, Dutton S, Ravaud P, Altman DG. Reporting and interpretation of randomized controlled trials with statistically nonsignificant results for primary outcomes. *JAMA* 2010; 303(20): 2058-64.

14. Ochodo EA, de Haan MC, Reitsma JB, Hooft L, Bossuyt PM, Leeflang MM. Overinterpretation and misreporting of diagnostic accuracy studies: evidence of "spin". *Radiology* 2013; 267(2): 581-8.

15. Altman DG, Simera I, Hoey J, Moher D, Schulz K. EQUATOR: reporting guidelines for health research. *Lancet* 2008; 371.

16. Stevens GA, Alkema L, Black RE, et al. Guidelines for Accurate and Transparent Health Estimates Reporting: the GATHER statement. *PLoS Med* 2016; 13(6): e1002056.

17. Tong A, Sainsbury P, Craig J. Consolidated criteria for reporting qualitative research (COREQ): a 32-item checklist for interviews and focus groups. *Int J Qual Health Care* 2007; 19(6): 349-57.

18. Juttmann RE. Het domein van gezondheidszorgonderzoek. In: Plochg T, Juttmann RE, Klazinga NS, Mackenbach JP, eds. Handboek gezondheidszorgonderzoek. Houten: Bohn Stafleu van Loghum; 2007: 22-23. Dutch.

19. Lohr KN, Steinwachs DM. Health services research: an evolving definition of the field. *Health Serv Res* 2002; 37(1): 7-9.

20. von Elm E, Altman DG, Egger M, et al. [The Strengthening the Reporting of Observational Studies in Epidemiology (STROBE) statement: guidelines for reporting observational studies]. *Rev Esp Salud Publica* 2008; 82(3): 251-9.

21. IBM Corp. Released 2016. IBM SPSS Statistics for Windows, Version 24.0. Armonk, NY: IBM Corp.

22. Lavis JN, Ross SE, Hurley JE, et al. Examining the role of health services research in public policymaking. *Milbank Q* 2002; 80(1): 125-54.

23. Hanney SR, Gonzalez-Block MA, Buxton MJ, Kogan M. The utilization of health research in policy-making: concepts, examples and methods of assessment. *Health Res Policy Syst* 2003; 1.

24. Kuruvilla S, Mays N, Walt G. Describing the impact of health services and policy research. *J Health Serv Res Policy* 2007; 12 Suppl 1: S1-23-31.

25. Burstein P. The impact of public opinion on public policy: A review and an agenda. *Polit Res Quart* 2003; 56(1): 29-40.

26. Glasziou P, Altman DG, Bossuyt P, et al. Reducing waste from incomplete or unusable reports of biomedical research. *The Lancet* 2014; 383(9913): 267-76.

27. Pittman P. Health Services Research in 2020: Data and Methods Needs for the Future. *Health Services Research* 2010; 45(5 Pt 2): 1431-41.

28. Wager E, Middleton P. Technical editing of research reports in biomedical journals. Cochrane Database Syst Rev 2008; (4): MR000002.

29. Fanelli D, Costas R, Lariviere V. Misconduct Policies, Academic Culture and Career Stage, Not Gender or Pressures to Publish, Affect Scientific Integrity. PLoS One 2015; 10(6): e0127556.

30. HSR-Europe. Health services research: helping tackle Europe's health care challenges. Policy Brief. Utrecht: NIVEL, Technische Universität Berlin, National Board of Health - Danish Centre for Health

For peer review only - http://bmjopen.bmj.com/site/about/guidelines.xhtml

Technology Assessment, Academic Medical Centre - University of Amsterdam, the London School of Hygiene and Tropical Medicine, 2011.

31. Horton R. The hidden research paper. *JAMA* 2002; 287(21): 2775-8.

32. Atkins D, Best D, Briss PA, et al. Grading quality of evidence and strength of recommendations. *BMJ* 2004; 328(7454): 1490.

33. de Groot AD. Methodologie. Grondslagen van onderzoek en denken in de gedragswetenschappen. Assen: Van Gorcum; 1994. Dutch.

For peer review only

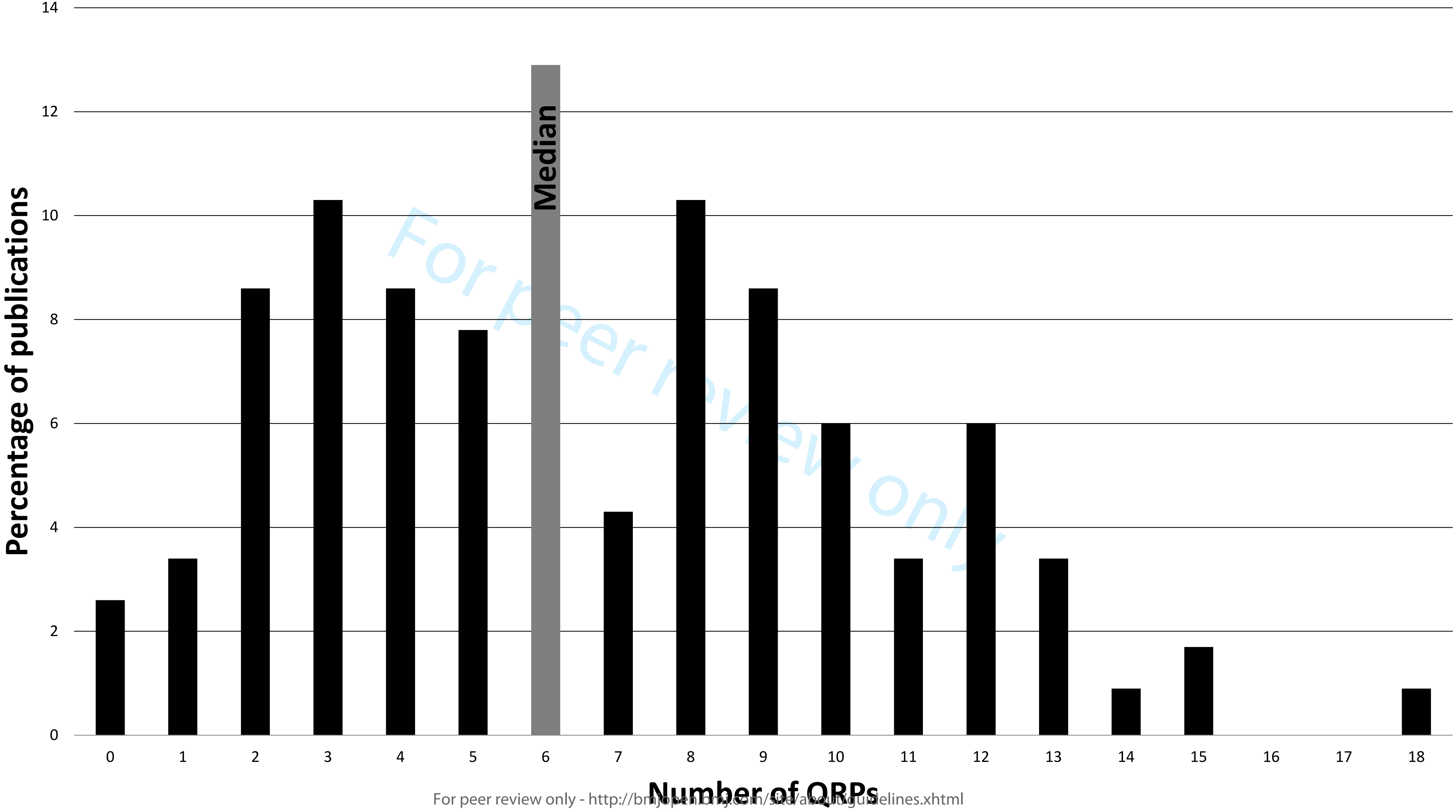
Percentage of publications
Median
0
2
4
6
8
10
12
14
0
1
2
3
4
5
6
7
8
9
10
11
12
13
14
15
16
17
18
Number of QRPs

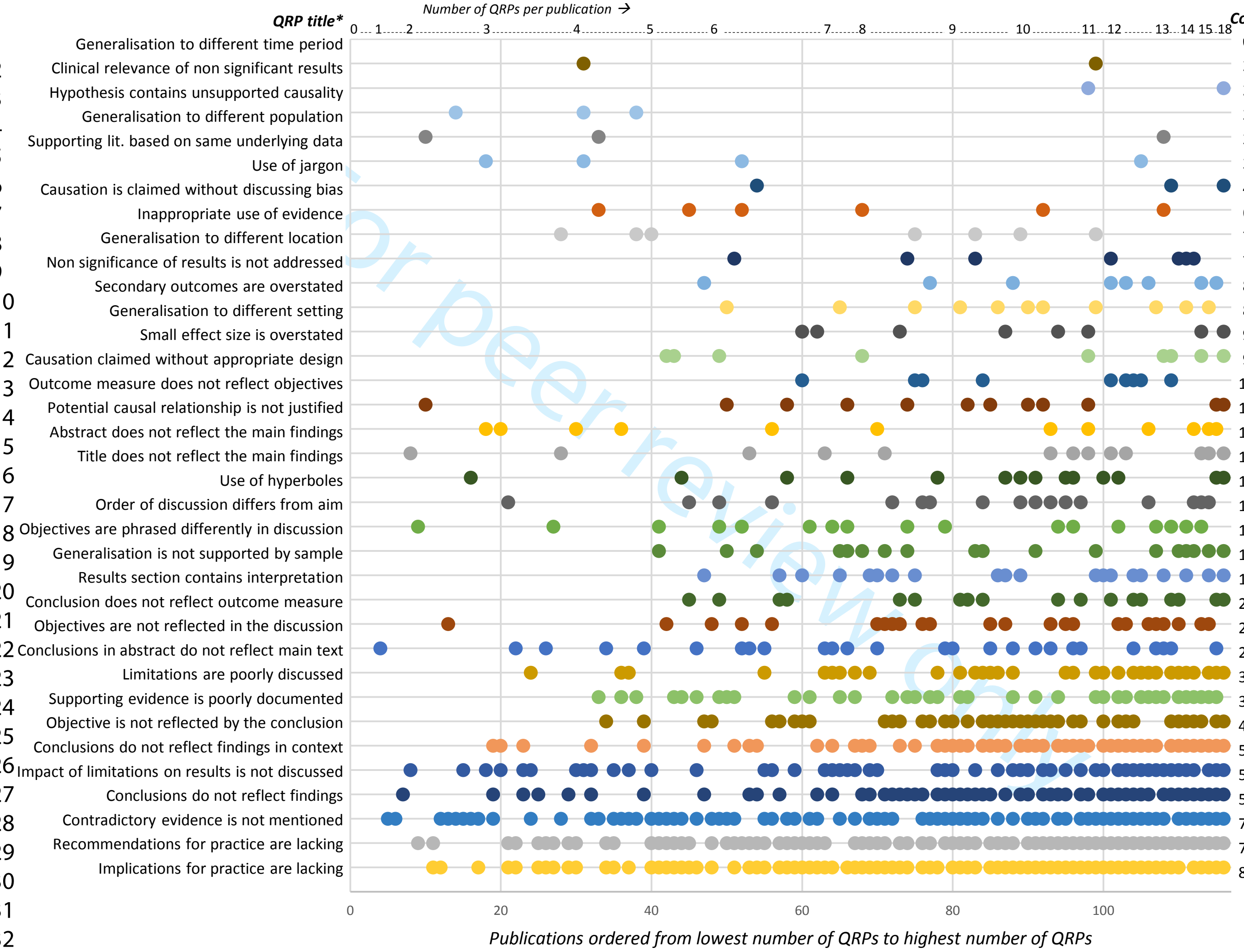

*The full QRP name is provided in supplementary material 3, table 1 ordered from least frequently found (Generalisation to different time period) to most frequently found (Implications for practice are lacking) QRP.'

For peer review only - http://bmjopen.bmj.com/site/about/guidelines.xhtml

8 November 2018, DEF in Word format

Manuscript assessment & data extraction form (DEF)

Item

1	**Assessor**	
1.1	Name	
1.2	Assessor role	
1.3	Assessor code	

2	**General information**	
2.1	Title of the study	
2.2	Journal	
2.3	Number of authors	
2.4	HSR (main) domain	
2.5	Involved institutions	
2.6	Funder(s) of the study	
2.7	Role of funder in the study	
2.8	Contribution of authors is stated	
2.9	Competing interests	
2.10	EQUATOR checklist available in additional materials	
2.11	Trial registration/protocol published	

3	**Introduction**		**Specify**	**Evaluation/comments**
3.1	The objective(s) of the study are reported in the introduction			
3.2	The research question(s) are reported in the introduction			
3.3	The context of the study is explained			

4	**Methods**		**Specify**	**Evaluation/comments**
4.1	Methodological approach			
4.2	Type of research			
4.3	Research design			
4.4	Data source is reported			
4.5	Selection of participants/sample is reported			
4.6	Non-response is reported			
4.7	Size of the study is reported			
4.8	Main outcome measure(s) are reported			
4.9	Secondary outcome measure(s) are reported			
4.10	Independent variable(s) are reported			

8 November 2018, DEF in Word format

4.11	Description of quantitative and/or qualitative methods of analyses is reported			
4.12	Handling of missing data is reported			
4.13	Comparator is explained			

**5**	**Results**		**Specify**	**Evaluation/comments**
5.1	Tables properly represent results			
5.2	Graphs properly represent results			
5.3	(Statistical) uncertainty is reported			

**6**	**Questionable messages and conclusions**	**QRP observed** (0=no; 1= yes; -8 not applicable; -9=not assessable)	**Evaluation/ comments** (rationale for assessment of QRP)	**Consulted project member** (X= consulted for advice concerning methods, specifics about study, etc.)
6.1	*Conclusions and key messages do not adequately reflect the objectives, design and actual findings*			
6.1.1	The title does not adequately reflect the main findings.			
6.1.2	The abstract does not adequately reflect the main findings.			
6.1.3	The conclusions in the abstract do not adequately reflect the conclusions in the main text.			
6.1.4	The objectives/research questions of the study are differently phrased in the introduction and the discussion.			
6.1.5	The outcome measure does not adequately reflect the objectives/research questions of the study.			
6.1.6	The main results discussed in the discussion paragraph do not adequately address the original objectives/research questions as posed in the introduction.			
6.1.7	The order of presenting the results in de discussion is inconsistent with the ordering of the objectives/research questions as posed in the introduction.			
6.1.8	The conclusions do not adequately reflect the objectives of the study.			

For peer review

6.1.9	The conclusions do not adequately reflect the findings as presented in the results paragraph.			
6.1.10	The outcome measure used does not allow the conclusions that are stated.			
6.1.11	The conclusion/discussion distracts from main outcomes by overstating the relevance of secondary outcomes.			
6.1.12	The conclusions are not supported by the results as presented in context of the referenced literature.			
6.1.13	Recommendations do not adequately reflect the results in context of the referenced literature.			
6.1.14	Implications for policy and practice do not adequately reflect the results in the context of the referenced literature.			
6.1.15	Lack of distinction between results and discussion. The results section contains elements of discussion and interpretation beyond the scope of explaining the results.			

6.2	*Main results are not or inadequately interpreted into the context of evidence*			
6.2.1	Supporting evidence is poorly documented.			
6.2.2	Contradicting evidence is poorly documented.			
6.2.3	Evidence is used inappropriately to support the findings (i.e. the argument is not supported by the actual message of the cited evidence). Will be measured as: Evidence seems to be used selectively to support the findings, given the title of the referenced evidence.			
6.2.4	The main source of evidence to support the results is based on the same underlying data.			

6.3	*Limitations are not adequately mentioned*			
6.3.1	Sources, direction and magnitude of bias are not or poorly discussed, or just listed without further discussion.			
6.3.2	The possible impact of the limitations on the results (i.e., magnitude and direction of any potential sources of bias) is not or poorly discussed.			

8 November 2018, DEF in Word format

6.4	*Unjustified generalisations*			
6.4.1	The sampling methodology does not allow the type of generalization provided.			
6.4.2	Generalization of findings to populations not included in the original sample is not justified.			
6.4.3	Generalization of findings to time periods not included in the original study is not justified.			
6.4.4	Generalization of findings to geographical locations not included in the original study is not justified.			
6.4.5	Generalization of findings to settings/institutions not included in the original study is not justified.			

6.5	*Unjustified causation*			
6.5.1	Causative wording is used in the hypothesis/research question, although there is no theory supporting causation.			
6.5.2	A causal relationship is claimed, although the research design is not appropriate to determine causation (methods lack control of potential confounding or systematic bias).			
6.5.3	A causal relationship is claimed although potential sources of bias and their potential impact on the findings were not discussed.			
6.5.4	A potential causal relationship claimed in the discussion paragraph is not justified.			

6.6	*Effect size*			
6.6.1	The relevance of statistically significant results with small effect size is overstated.			
6.6.2	The possible clinical relevance of statistically nonsignificant results is not addressed.			
6.6.3	Non-significant results are discussed without addressing significance			

6.7	*Inappropriate use of language*			
6.7.1	Hyperboles and exaggerating adjectives are unjustifiably used (such as: key, groundbreaking, ideal, excellent, great, brilliant,			

8 November 2018, DEF in Word format

	extraordinary, impressive, completely, absolutely, entirely, everywhere, everything, nothing, beyond any doubt, definitely).			
6.7.2	Jargon, technical and complex language, that does not fit the journal audience, are used without properly explaining the meaning.			

7 Miscellaneous

7.1	Overall qualitative evaluation of the study (e.g. quality, reporting style).		
7.2	Other comments.		

8 Advice needed from second assessor

8.1	About the contents of the article		
8.2	Second assessment recommended		

For peer review only

8 November 2018, DEF in Word format

Instructions per item

1 Assessor

1.1	Name	
1.2	Assessor role	
1.3	Assessor code	

2 General information

		Instructions
2.1	Title of the study	
2.2	Journal	
2.3	Number of authors	
2.4	HSR (main) domain	*Choose main discipline from list, add other disciplines in entry field*
2.5	Involved institutions	*List all-in*
2.6	Funder(s) of the study	
2.7	Role of funder in the study	*Copy funder declaration*
2.8	Contribution of authors is stated	
2.9	Competing interests	*Copy competing interest declaration*
2.10	EQUATOR checklist available in additional materials	
2.11	Trial registration/protocol published	*As mentioned in the article*

3 Introduction

3.1	The objective(s) of the study are reported in the introduction	
3.2	The research question(s) are reported in the introduction	
3.3	The context of the study is explained	

4 Methods

4.1	Methodological approach	
4.2	Type of research	
4.3	Research design	
4.4	Data source is reported	*e.g. registration, scientific or grey literature, survey data, interview data*
4.5	Selection of participants/sample is reported	*Selection of study enrolees also included case studies*
4.6	Non-response is reported	
4.7	Size of the study is reported	
4.8	Main outcome measure(s) are reported	

For peer review only

8 November 2018, DEF in Word format

4.9	Secondary outcome measure(s) are reported	
4.10	Independent variable(s) are reported	
4.11	Description of quantitative and/or qualitative methods of analyses is reported	
4.12	Handling of missing data is reported	
4.13	Comparator is explained	

**5**	**Results**	
5.1	Tables properly represent results	*Tables give a reflection of actual results instead of cherry picking*
5.2	Graphs properly represent results	*Scaling is appropriate*
5.3	(Statistical) uncertainty is reported	*Confidence intervals are provided for the main results*

**6**	**Questionable messages and conclusions**	**Instructions**
6.1	*Conclusions and key messages do not adequately reflect the objectives, design and actual findings*	
6.1.1	The title does not adequately reflect the main findings.	*Title includes a quote or statement that does not accurately reflect/refers to the main findings, or deviates from the findings.*
6.1.2	The abstract does not adequately reflect the main findings.	*The abstracts contents deviate from / contradict with the main findings in the article text. Messy writing is not considered a QRP. Specifically for the conclusion in the abstract, causative wording misses: the conclusion in the abstract suggests causation, although the conclusions as discussed in the discussion paragraph report correlation. For instance, it is an unbalanced representation of the main results by focussing on secondary findings, while reducing the importance of the main findings, or reflects cherry-picking from the most conspicuous results. Or the stated results in the abstract in qualitative studies do not appear in the main text.*
6.1.3	The conclusions in the abstract do not adequately reflect the conclusions in the main text.	*The conclusions in the abstract are short-sighted compared to the actual conclusions in the main text. Conclusions can be stated in the discussion paragraph and/or the conclusion paragraph.*

8 November 2018, DEF in Word format

6.1.4	The objectives/research questions of the study are differently phrased in the introduction and the discussion.	*When reporting objectives/research questions in the discussion. Different wording: does not need to include the exact wording, however the meaning/connotation should be similar. Different ordering of objectives/research questions.*
6.1.5	The outcome measure does not adequately reflect the objectives/research questions of the study.	*The objectives /research questions cannot be answered with the outcome measure that is studied*
6.1.6	The main results discussed in the discussion paragraph do not adequately address the original objectives/research questions as posed in the introduction.	*The research questions and/or objectives that were stated in the introduction section are not or only partly answered by the main results*
6.1.7	The order of presenting the results in de discussion is inconsistent with the ordering of the objectives/research questions as posed in the introduction.	*Not an actual QRP, but it does conflict with transparency in presenting the study's findings. If there's just one objective/research question, this item is not applicable (no structuring possible) and should be scored -8.*
6.1.8	The conclusions do not adequately reflect the objectives of the study.	*The objectives of the study are not met by the conclusions the study arrives at. Conclusions can be stated in the discussion paragraph and/or the conclusion paragraph. Either the study along the way shifted perspective, however no justification is provided. Or the write-up of the conclusions is flawed. Framing conclusion as extension to the discussion is not a QRP (undesirable, however beyond the scope if this indicator).*
6.1.9	The conclusions do not adequately reflect the findings as presented in the results paragraph.	*The conclusions deviate from the the main findings. Conclusions can be stated in the discussion paragraph and/or the conclusion paragraph. The conclusion section does often not contain actual conclusions. The actual conclusion is often presented in the discussion section. Hence, conclusions in the discussion section are considered conclusions as well. Concluding statements will be marked, those statements that are only used to frame results (emphasizing importance of the study) are not considered conclusions. Key messages (in a box as seperate section in some journals) are also considered conclusions.*<br>*For instance, it is an unbalanced representation of the main results by focussing on secondary findings, while reducing the importance of the main findings, or reflects cherry-picking from the most conspicuous*

For peer review only

8 November 2018, DEF in Word format

		results. *If new results are presented in the discussion section, then this is a QRP. (Assessors should not recalculate results)*
6.1.10	The outcome measure used does not allow the conclusions that are stated.	*For instance: the conclusions are about the quality of the health care system, whereas the outcome measure was 'satisfaction with home-care for elderly'*
6.1.11	The conclusion/discussion distracts from main outcomes by overstating the relevance of secondary outcomes.	*The main outcomes are ignored or their importance reduced, while favouring secondary outcomes. Most space is taken by discussing these secondary outcomes.*
6.1.12	The conclusions are not supported by the results as presented in context of the referenced literature.	*If the conclusion is not based on the results, but only on referenced literature, then this is noted as QRP (as aligns with 6.1.9). The extent of the conclusions is broader/more far fetching than the findings of the study, backed-up by discussed literature, justify. Conclusions can be stated in the discussion paragraph and/or the conclusion paragraph. For instance, a relationship between IV and DV is exaggerated. Conclusions cannot be stated based on referenced literature alone, main results are the fundament for the conclusions, that may be extended based on referenced literature.*
6.1.13	Recommendations do not adequately reflect the results in context of the referenced literature.	*Recommendations: what can/should be done with the studies findings? Recommendations are based on the results from the study, not only on the referenced literature. The extent of the recommendations is broader/more far fetching than the findings of the study, backed-up by discussed literature, justify. For instance, a relationship between IV and DV is exaggerated. QRP if no justification for the suggested recommendation is provided. QRP if no recommendation is provided.*
6.1.14	Implications for policy and practice do not adequately reflect the results in the context of the referenced literature.	*Implications: what are the consequences for policy and practice if the recommendations are followed-up? What would happen if the recommendations are carried out. (e.g. recommendations = implement the intervention in this setting, implication = the outcomes may improve by this much.) QRP if no justification for suggested implication is provided, QRP if no implication is provided. Originally: implications for*

For peer review only - http://bmjopen.bmj.com/site/about/guidelines.xhtml

		policy and practice are poorly mentioned. Instruction: implications for practise and policy are well-balanced and give actual meaning to the findings of the study in context of practice and/or policy.
6.1.15	Lack of distinction between results and discussion. The results section contains elements of discussion and interpretation beyond the scope of explaining the results.	*Applicable to all designs. Pilot included qualitative study, but also applies to quantitative studies. Results are placed in the context of literature beyond the theoretical model of the study.*

6.2	*Main results are not or inadequately interpreted into the context of evidence*	
6.2.1	Supporting evidence is poorly documented.	*Only limited evidence to support the main results is provided and only superficially discussed. No thorough reflection of the findings in perspective of supporting evidence.*
6.2.2	Contradicting evidence is poorly documented.	*Only limited evidence to oppose against the main results is provided and only superficially discussed. No thorough reflection of the findings in perspective of contradicting evidence.*
6.2.3	Evidence is used inappropriately to support the findings (i.e. the argument is not supported by the actual message of the cited evidence). Will be measured as: Evidence seems to be used selectively to support the findings, given the title of the referenced evidence.	*State inappropriately cited references, and explain why inappropriate: the evidence ascribed to the reference deviates from what could be assumed based on the title of the reference. Includes supporting results through self-citation (without further explanation of self-citation). Self-citation is not a QRP if clearly stated "in an earlier study we found..." If no references are used to support the results (QRP 6.2.1/2), then this is no QRP (QRP is avoided by not using literature), thus assessment is not possible ans should be scored -9.*
6.2.4	The main source of evidence to support the results is based on the same underlying data.	*Most supporting evidence is grounded in the same data source as was used for the reviewed study (not necessarely self-citing), inducing circularity in argumentation.*

6.3	*Limitations are not adequately mentioned*	
6.3.1	Sources, direction and magnitude of bias are not or poorly discussed, or just listed without further discussion.	*Are the (relevant) limitations mentioned? The implications of the study design, methodology, sampling, context, etc. for risk of biasing study findings are not thoroughly discussed.*

8 November 2018, DEF in Word format

6.3.2	The possible impact of the limitations on the results (i.e., magnitude and direction of any potential sources of bias) is not or poorly discussed.	*Is the impact of limitations discussed (if no limitations are mentioned then this is considered a QRP). The extent to which potential risks of bias affect the interpretation of the findings is not thoroughly discussed.*

6.4	*Unjustified generalisations*	
6.4.1	The sampling methodology does not allow the type of generalization provided.	*The sample is too specific, small, or flawed (for instance by attrition, selection bias) for the generalization that is made.*
6.4.2	Generalization of findings to populations not included in the original sample is not justified.	*The included sample is too specific, small or flawed (for instance by attrition, selection bias) and no or inadequate evidence is provided to support the generalization that is made. Population does not include geographical location (this is a separate QRP). Population includes population characteristics such as gender, ethnicity, age, etc.*
6.4.3	Generalization of findings to time periods not included in the original study is not justified.	*The characteristics of the included time period are too specific (for instance in election period, affecting the policy that was studied) and no or inadequate evidence is provided to support the generalization that is made*
6.4.4	Generalization of findings to geographical locations not included in the original study is not justified.	*The characteristics of the included igeographical location(s) are too specific to generalise to other geographical locations (for instance very urbanised area to rural setting) and no or inadequate evidence is provided to support the generalization that is made*
6.4.5	Generalization of findings to settings/institutions not included in the original study is not justified.	*The characteristics of the included institutions are too specific to generalise to other institutions (for instance hospital regulations to nursing homes) and no or inadequate evidence is provided to support the generalization that is made*

6.5	*Unjustified causation*	
6.5.1	Causative wording is used in the hypothesis/research question, although there is no theory supporting causation.	*Quantitative: hypothesis is not justified/allowed since there's no theory to support a causal relationship*
6.5.2	A causal relationship is claimed, although the research design is not appropriate to determine causation	*No causation based on the results of the present study may be assumed if no RCT is conducted… (or longitudinal cohort?)*

For peer review only

	(methods lack control of potential confounding or systematic bias).	
6.5.3	A causal relationship is claimed although potential sources of bias and their potential impact on the findings were not discussed.	*No or inadequate discussion is included concerning the impact of potential sources of bias on the possible causation that was found in the results*
6.5.4	A potential causal relationship claimed in the discussion paragraph is not justified.	*When a causal relation may not be assumed solely based on the study's findings, no or inadequate supporting and contradicting evidence is used to discuss the possible causation that was found in the results.*

6.6	*Effect size*	
6.6.1	The relevance of statistically significant results with small effect size is overstated.	*Importance of findings is exaggerated. Although (some) results are statistically significant, the clinical/practical relevance is minor due to small effect size/causation is unlikely.*
6.6.2	The possible clinical relevance of statistically nonsignificant results is not addressed.	*Importance of findings is dismissed, since no statistical significance was reached. Although the findings reflect likely causation and non-significance was likely due to lack of power.*
6.6.3	Non-significant results are discussed without addressing significance	*Results are discussed as if they were significant, without addressing they are not, or what the uncertainty is.*

6.7	*Inappropriate use of language*	
6.7.1	Hyperboles and exaggerating adjectives are unjustifiably used (such as: key, groundbreaking, ideal, excellent, great, brilliant, extraordinary, impressive, completely, absolutely, entirely, everywhere, everything, nothing, beyond any doubt, definitely).	*The use of adjectives that exaggerate the relevance of the findings, conclusions and messages. Not actually counting adjectives, if one hyperbole is used and attracted the attention. Hyperbolic adjective use per se is no QRP, only in relation to results/conclusions, to exaggerate the study's findings.*
6.7.2	Jargon, technical and complex language, that does not fit the journal audience, are used without properly explaining the meaning.	*The journal audience is not properly addressed by the language used. Language use seems to be overly complex to impress or distract the reader.*

7 Miscellaneous

7.1	Overall qualitative evaluation of the study (e.g. quality, reporting style).	*If a certain aspect impacts the answer to multiple questions, specify in "other comments". E.g. if the discussion section does not contain main results, then this item cannot be assessed.*

For peer review only - http://bmjopen.bmj.com/site/about/guidelines.xhtml

7.2	Other comments.	

8 Advice needed from second assessor

8.1	About the contents of the article	*What advice is needed, state question.*
8.2	Second assessment recommended	*First assessor doubts about assessment and requests second opinion.*

For peer review only

February 5th, 2019

Supplement to the methods section of:

The occurrence and nature of questionable research practices in the reporting of messages and conclusions in international scientific Health Services Research publications: A structured assessment of publications authored by researchers in the Netherlands

Additional information to the methods of the development of the definition and measurement instrument for "questionable research practices in the reporting of messages and conclusions in scientific health services research publications"

This document describes the methods used to develop a definition of questionable research practices (QRPs) in the reporting of messages and conclusions, and to construct a measurement instrument that allows for the identification of questionable research practices in the reporting of messages and conclusions in Health Services Research (HSR).

Methodology
Methods included an explorative review of definitions in literature, a consultation meeting with the project group, institution/department leaders of Dutch HSR institutions and project advisors (n=13), semi-structured interviews with 13 HSR institutes (n=19) and an expert consultation (n=5).

Setting
13 HSR groups, departments, or institutions (hereafter referred to as "HSR institutions") in the Netherlands, including both academic and non-academic institutions participated in this study. These institutions all agreed to participate in an effort to assure the overall quality of HSR publications in the Netherlands.

Literature review
First, a literature review was conducted searching for existing definitions of questionable research practices in the reporting of conclusions and messages, and operationalisations of QRPs. Search terms included in different order and combination: 'questionable research practices', 'spin', 'over interpretation', 'discordant conclusions', 'QRPs', 'outcome reporting bias', 'questionable conclusions' and 'responsible conclusions'. Documents were included if they described methods to measure questionable research practices in scientific publication, or provided definitions of the above key terms. Referred documents that fit the criteria were also included in the review.

After identifying the main literature that suited our aim, we came to a preliminary definition of QRPs based on Boutron 2010, Ochobo 2013, and Horton 1995 [1-3].

An extensive list of possible types of QRPs in the reporting of messages and conclusions was developed, based on the EQUATOR checklists[4] and instruments from previous studies. For example, instruments for identifying 'spin', reporting of qualitative research and other QRPs such as [3,5-7]. Spin in this context refers to "a way to distort science reporting without actually lying")

Consultation meeting
Second, we presented the preliminary QRP definition and the first draft of items referring to QRPs (see page 3) during a consultation meeting of participating HSR institutions on 6 June 2017. The meeting lasted three hours, during which the research project and the preliminary definition and draft of QRP items was discussed. Representatives of the participating HSR institutions (n=7), project advisors (n=2) and project group members (n=4) attended the meeting. The attendees discussed their thoughts about the definition and its operationalisation. Detailed notes from this meeting were summarized and shared with the representatives of all participating institutes (including those who did not attend).

The central conclusion of the meeting was to focus on the 'measurability' of the QRPs. An important consideration in developing the instrument for the assessment of scientific publication is to focus on the possibility to measure the QRPs. Therefore, the focus should be on QRPs that can be quantified. These should be distinguished from QRPs that, although possibly important, are not quantifiable.

For peer review only - http://bmjopen.bmj.com/site/about/guidelines.xhtml

February 5th, 2019

Semi-structured interviews
Third, we conducted fourteen semi-structured interviews with nineteen leaders/representatives of the thirteen HSR institutions. These representatives had to have a clear overview of the process of reporting research in their institute. One of the institutions was represented by two separate departments, hence two representatives were separately interviewed. Three interviews were conducted with both the institute leader and a second representative. One of the interviews included three representatives of an institution. The aim of the interviews was to discuss our draft of QRP items and identify additional measurable QRPs in the reporting of messages and conclusions in HSR, explore potential causes of QRPs in messages and conclusions, and to discuss experiences of the institute leaders with these QRPs. A semi-structured interview guide was developed by the project team (see page 4-5). During the interview, we presented the interviewees with a draft of QRP items. The draft list was iteratively adjusted, i.e. after each interview we drafted a new version including the findings of the previous interviews.

Interviewees were approached through e-mail to schedule an appointment. Two researchers conducted the interviews of which thirteen took place at the participating institutions and one interview took place in a public space. During the first interview, both researchers were present to align the approach. The remainder of the interviews were equally divided between them. The interviews lasted one hour. In concordance with ethical guidelines, the goal of the interview was explained at the start of the interview and permission to audio-record the interview was obtained.

With the support of the recordings, a report was written and shared with the interviewees for validation. All interviewees confirmed the reports, after mostly minor edits to the report. From the interview reports, we drew up a new draft of the list of QRP items (see page 6-7). In the research group, we specifically paid attention to correct wording of the QRPs.

Expert consultation
Fourth, ten leading international health services researchers were asked to provide feedback on this list of QRP items. These HSR experts were invited through e-mail in which we explained the aim of the study, and included the definition of QRPs and the list of QRP items. Five experts provided their comments to the items. Five experts did not respond after a reminder, or indicated not having time to review the QRP items. Feedback was summarized, and comments were used to adapt the QRP definition and list of QRP items.

Measurement instrument
We developed the measurement instrument in Excel format by taking items from earlier developed checklists (EQUATOR and COREQ) and the list of QRPs. The measurement instrument was completed after a final consensus meeting of the research group. The measurement instrument exists of three sections: 1) bibliographic information of the publication (eg. funder, journal, number of authors), 2) basic methodological information (eg. included population, analyses method) and 3) possible QRPs in messages and conclusions. A pilot was conducted to assess the feasibility and usability of the instrument. In the pilot, two project members independently assessed five international HSR publications to identify modifications needed to improve the items in the instrument, and to align the interpretation of the items. The project group discussed the proposed modifications, resulting in the final version: the data extraction form (see supplementary material 1.)

For peer review only - http://bmjopen.bmj.com/site/about/guidelines.xhtml

February 5th, 2019

List of possible questionable research practices presented during the consultation meeting and the interviews

With each interview, new QRP's were added to the list which were then presented during the next interview.

Definition: Questionable reporting of messages and conclusions:

> *"The use of reporting, from whatever motive, consciously or unconsciously, to make conclusions or messages weaker or stronger than results justify."*

For peer review only

Potential

1. Poorly set results into context of totality of evidence
2. No mention of contradictory evidence
3. Discrepancy between the aim of the study and the conclusion
4. No reporting objectives, aim or research question
5. Concealing limitations
6. Lack of transparency of methods used
7. *Not reporting a hypothesis*
8. Selective reporting of results in conclusion
9. No mention of [statistical] uncertainty

Actual

1. Discrepancy between the title, abstract and the article
2. Describing unjustified causation
3. Inappropriate citing
4. Authorial rhetoric
5. Misleading graphs and tables
6. Unjustified generalisations (mismatch between study population, sex, geographical entities and time period)
7. *Stating the [intervention/measure] is beneficial despite statistically nonsignificant difference for the primary outcome*
8. *Distract the reader from statistically nonsignificant results*
9. Not explaining the comparator/context of the intervention

For peer review only - http://bmjopen.bmj.com/site/about/guidelines.xhtml

February 5th, 2019

Interview guide used during the semi-structured interviews (in Dutch)

Interviewprotocol eerste consultatieronde juni/juli 2017

Toelichting op het interview

Het doel van het ZonMw Project is om te komen tot aanbevelingen ter bevordering van verantwoord rapporteren over gezondheidszorgonderzoek (responsible conclusions and messages in health services research).

Hoewel de projectleiding primair bij het AMC ligt, is het binnen het project nadrukkelijk een gedeelde verantwoordelijkheid van alle dertien betrokken instituten om te komen tot voorstellen ter bevordering van verantwoord rapporteren van gezondheidszorgonderzoek. We houden in deze eerste fase interviews met de hoofden en vertegenwoordigers van de betrokken HSR instituten. Tijdens het interview worden de volgende onderwerpen besproken:

1) Potentiele oorzaken van QRPs in het vormen van conclusies en berichten
2) Het meten van QRP in conclusies en berichten in HSR
3) Uw ervaring met Responsible en Questionable Research Practices.

Het interview zal 1 uur in beslag nemen. Indien u daarvoor toestemming geeft, zal het gesprek worden opgenomen, en notities van het gesprek zullen worden uitgewerkt. Het gespreksverslag zal vervolgens ter verificatie aan u worden voorgelegd. Het gesprek wordt vertrouwelijk behandeld; alleen de onderzoekers op dit project zullen inzicht hebben in de inhoud van dit gesprek. De rapportage van de bevindingen zal op geaggregeerd niveau plaatsvinden. Uitspraken zullen daarbij niet-herleidbaar tot persoon en/of instituut worden gerapporteerd.

Wij zullen eerst onze bevindingen tot nu toe kort toelichten, en vervolgens verdergaan met het interview.

1) Bevindingen startbijenkomst
    a. Positieve start
    b. Nadruk op betrokkenheid alle instituten (veel feedbackloops)

For peer review only - http://bmjopen.bmj.com/site/about/guidelines.xhtml

February 5th, 2019

Vragen

Probleem onderkenning

1. Acht U het zinvol om naar ongeoorloofd rapporteren (QRP) bij gezondheidszorg onderzoek in NL te kijken?
    a. Indien ja, waarom denkt U dat het een reëel probleem is
    In de omvang, neemt het toe of af?

Beleid & structuur (wat doe u in uw rol als instituutshoofd om dit probleem aan te pakken)

2. Kunt u iets vertellen over de wijze waarop er binnen uw instituut wordt omgegaan met het stimuleren van verantwoord rapporteren van (HSR) onderzoek?
    a. Is er specifiek beleid op het verantwoord rapporteren van HSR?
    Indien ja, kunt u dat toelichten? (Open doorvragen, voorbeelden)

    b. Is er specifiek beleid, procedures, werkwijzen om QRPs in rapporteren van HSR te voorkomen?
    Indien ja, kunt u dat toelichten? (Open doorvragen, voorbeelden)

Definitie QRPs

Tot nu toe hebben wij de volgende QRPs geïdentificeerd in het rapporteren van onderzoek in wetenschappelijke publicaties [lijst QRPs].

3. Bent u het eens met (de formulering van) deze QRPs en heeft u opmerkingen en aanvullingen op deze lijst?
    a. Wat wilt u veranderen en of toevoegen?
4. Wij willen een keuze maken uit specifieke, goed te meten QRPs in het rapporteren van conclusies en berichten in HSR. Welke QRPs voldoen naar uw mening aan deze criteria?

Ervaringen

5. Wat zijn uw ervaringen met het rapporteren van resultaten van gezondheidszorgonderzoek?
    a. Wat gaat naar uw ervaring goed?
    b. Wat zijn in uw ervaringen knelpunten? Kunt u voorbeelden noemen waarin deze knelpunten naar voren kwamen?

Toelichting framework

Op dit moment hebben wij de factoren van invloed QRP als volgt weergeven [framework].

6. Zijn dit volgens u juiste factoren?
7. Wat zijn naar uw mening (nog meer) belangrijke factoren van invloed op RRPs en QRPs?
8. Welke factoren zou u als eerste aanpakken? Waarom deze factoren? Wat verwacht u daarvan?

Afsluiting

9. Heeft U nog aanvullende suggesties hoe in samenwerking met de andere gezondheidszorgonderzoeksinstituten in NL kan worden bijgedragen aan verantwoord rapporteren?

For peer review only - http://bmjopen.bmj.com/site/about/guidelines.xhtml

February 5th, 2019

QRP list and comment form used for expert consultation

Experts provided comments in the comment boxes

Questionable reporting of conclusions and messages in Health Services Research

Expert consultation

Definition:

"To frame, from whatever motive, consciously or unconsciously, conclusions or messages as an answer to the research question that are not justified by the results"

[Comments concerning definition]

Measuring questionable reporting of conclusions & messages

Title, abstract, main text, and conclusions do not align

1.1. The title does not align with the main text.
1.2. The abstract does not align with the main text.
1.3. The conclusions in the abstract do not align with the conclusions in the main text.
1.4. The objectives/research questions of the study are differently phrased in the introduction. and the discussion.

[Comments concerning category 1]

Conclusions do not reflect the objectives and results properly

2.1. The main results in the discussion do not follow from the research questions.
2.2. The conclusions do not align with the results in the main text.
2.3. The order of presenting the results is inconsistent with the research questions.
2.4. The conclusion/discussion distracts from main outcomes by overstating the relevance of secondary outcomes.
2.5. The relevance of statistically significant results with small effect sizes is overstated.
2.6. Possible clinical relevance of statistically insignificant results is not addressed.
2.7. The conclusions do not reflect the objectives of the study.
2.8. The conclusions are not supported by the results in context of the discussed literature.
2.9. Recommendations do not follow from the results and discussed literature.
2.10. Implications for policy and practice are poorly mentioned.

[Comments concerning category 2]

Main results are poorly put into the context of evidence

3.1. Supporting evidence is poorly mentioned.
3.2. Contradicting evidence is poorly mentioned.
3.3. Citations are used inappropriately to support the conclusions (i.e. the actual message of the cited source does not align with the conclusion it should support).
3.4. Self-citations or studies based on the same data are the main source of supporting evidence.

[Comments concerning category 3]

For peer review only - http://bmjopen.bmj.com/site/about/guidelines.xhtml

February 5th, 2019

Limitations are not properly mentioned

4.1. Limitations are discussed only superficially (for instance only on one level, e.g. the measurement level, design, sample).
4.2. No sources of bias are mentioned.
4.3. The possible effect of the limitations on the results is not discussed.

[Comments concerning category 4]

Unjustified generalizations

5.1. The time of data collection does not align with the time for which the conclusions are presented.
5.2. The study sample does not align with the population the conclusions are generalized to.
5.3. No justifications are offered for generalizations:
  5.3.1. In time.
  5.3.2. In geographical location.
  5.3.3. To setting/institution.

[Comments concerning category 5]

Unjustified causation

6.1. Causative wording that is used that is not allowed by the study design.
6.2. A causal relationship is claimed without mentioning any theoretical explanation of the relation.

[Comments concerning category 6]

Inappropriate language use

7.1. Hyperboles and exaggerating adjectives are used without justification (such as: ideal, excellent, great, brilliant, extraordinary, impressive, completely, absolutely, entirely, everywhere, everything, nothing, beyond any doubt, definitely).
7.2. Jargon, technical and complex language are used without properly explaining the meaning.

[Comments concerning category 7]

For peer review only - http://bmjopen.bmj.com/site/about/guidelines.xhtml

February 5th, 2019

References

1. Boutron I, Dutton S, Ravaud P, Altman DG: 'Spin' in reports of randomized controlled trials with nonstatistically significant primary outcomes. 6th International Congress on Peer Review and Biomedical Publication, Vancouver. (Accessed 30 March 2010), [http://www.ama-assn.org/public/peer/abstracts_2009.html#30].
2. Horton R. The rhetoric of research. *BMJ* 1995; **310**(6985): 985.
3. Ochodo EA, de Haan MC, Reitsma JB, Hooft L, Bossuyt PM, Leeflang MM. Overinterpretation and misreporting of diagnostic accuracy studies: evidence of "spin". *Radiology* 2013; **267**(2): 581-8.
4. Altman DG, Simera I, Hoey J, Moher D, Schulz K. EQUATOR: reporting guidelines for health research. *Lancet* 2008; **371**.
5. Stevens GA, Alkema L, Black RE, et al. Guidelines for Accurate and Transparent Health Estimates Reporting: the GATHER statement. *PLoS Med* 2016; **13**(6): e1002056.
6. Tong A, Sainsbury P, Craig J. Consolidated criteria for reporting qualitative research (COREQ): a 32-item checklist for interviews and focus groups. *Int J Qual Health Care* 2007; **19**(6): 349-57.
7. Boutron I, Dutton S, Ravaud P, Altman DG. REporting and interpretation of randomized controlled trials with statistically nonsignificant results for primary outcomes. *JAMA* 2010; **303**(20): 2058-64.

For peer review only

For peer review only - http://bmjopen.bmj.com/site/about/guidelines.xhtml

Supplementary material 3

Table 1. Occurrence of QRPs in the reporting of messages and conclusions in HSR publications (n=116), ordered from most frequently to least frequently occurring (%).

Questionable research practices (QRPs) in reporting messages and conclusions	% publications with QRP	% publications without QRP	% publications for which QRP not assessable
Implications for policy and practice do not adequately reflect the results in the context of the referenced literature.	**69·0	31·1	0·0
Recommendations do not adequately reflect the results in the context of the referenced literature.	***65·5	34·5	0·0
Contradicting evidence is poorly documented.	63·8	36·2	0·0
Conclusions do not adequately reflect the findings as presented in the results section.	46·6	51·7	1·7
Possible impact of the limitations on the results is not or poorly discussed.	44·0	56·0	0·0
Conclusions are not supported by the results as presented in the context of the referenced literature.	43·1	54·3	2·6
The conclusions do not adequately reflect the objectives of the study.	35·3	61·2	3·4
Supporting evidence is poorly documented.	31·9	68·1	0·0
Sources. direction and magnitude of bias are not or poorly discussed. or just listed without further discussion.	27·6	72·4	0·0
The conclusions in the abstract do not adequately reflect the conclusions in the main text.	22·4	75·0	2·6
The main results discussed in the discussion paragraph do not adequately address the original objectives/research questions as posed in the introduction.	20·7	75·9	3·4
The outcome measure used does not allow the conclusions that are stated. *	18·1	81·9	0·0
Lack of distinction between results and discussion. The results section contains elements of discussion and interpretation beyond the scope of explaining the results.	17·2	82·8	0·0
The sampling methodology does not allow the type of generalization provided.	15·5	84·5	0·0
The objectives/research questions of the study are differently phrased in the introduction and the discussion.	14·7	36·2	49·1
The order of presenting the results in de discussion is inconsistent with the ordering of the objectives/research questions as posed in the introduction.	14·7	75·0	10·3
Hyperboles and exaggerating adjectives are unjustifiably used	12·1	87·9	0·0
The title does not adequately reflect the main findings.	11·2	88·8	0·0
The abstract does not adequately reflect the main findings.	10·3	89·7	0·0
A potential causal relationship claimed in the discussion paragraph is not justified.	10·3	89·7	0·0
The outcome measure does not adequately reflect the objectives/research questions of the study. *	9·6	90·4	0·0
A causal relationship is claimed. although the research design is not appropriate to determine causation.	9·6	90·4	0·0
The relevance of statistically significant results with small effect size is overstated. *	9·6	90·4	0·0
Generalising findings to settings/institutions not included in the original study is not justified.	9·5	89·7	1·0
The conclusion/discussion distracts from main outcomes by overstating the relevance of secondary outcomes. *	8·4	91·6	0·0

For peer review only

Non-significant results are discussed without addressing significance.	8·4	91·6	0·0
Generalising findings to geographical locations not included in the original study is not justified.	6·0	94·0	0·0
Evidence is used inappropriately to support the findings.	5·2	94·9	0·0
A causal relationship is claimed although potential sources of bias and their potential impact on the findings were not discussed. *	3·6	96·4	0·0
Jargon. technical and complex language. that does not fit the journal audience. are used without properly explaining the meaning.	3·4	96·6	0·0
The main source of evidence for supporting the results is based on the same underlying data.	2·6	96·6	0·9
Generalising findings to populations not included in the original sample is not justified.	2·6	97·4	0·0
Causative wording is used in the hypothesis/research question, although there is no theory to support causation. *	2·4	97·6	0·0
Possible clinical relevance of statistically non-significant results is not addressed. *	2·4	97·6	0·0
Generalising findings to time periods not included in the original study is not justified.	0·0	100·0	0·0

** QRPs only applicable to quantitative research-based publications (n=83)*
*** 50 ·0% of publications did not mention implications for policy or practice.*
**** 34·5% of publications did not mention recommendations for policy or practice.*

For peer review only

STROBE Statement—checklist of items that should be included in reports of observational studies

	Item No	Recommendation	
**Title and abstract**	1	(*a*) Indicate the study's design with a commonly used term in the title or the abstract	No
		(*b*) Provide in the abstract an informative and balanced summary of what was done and what was found	Page 2
**Introduction**			
Background/rationale	2	Explain the scientific background and rationale for the investigation being reported	Page 3
Objectives	3	State specific objectives, including any prespecified hypotheses	Page 3
**Methods**			
Study design	4	Present key elements of study design early in the paper	Page 4
Setting	5	Describe the setting, locations, and relevant dates, including periods of recruitment, exposure, follow-up, and data collection	Page 4
Participants	6	(*a*) *Cohort study*—Give the eligibility criteria, and the sources and methods of selection of participants. Describe methods of follow-up<br>*Case-control study*—Give the eligibility criteria, and the sources and methods of case ascertainment and control selection. Give the rationale for the choice of cases and controls<br>*Cross-sectional study*—Give the eligibility criteria, and the sources and methods of selection of participants	Page 4
		(*b*) *Cohort study*—For matched studies, give matching criteria and number of exposed and unexposed<br>*Case-control study*—For matched studies, give matching criteria and the number of controls per case	n.a.
Variables	7	Clearly define all outcomes, exposures, predictors, potential confounders, and effect modifiers. Give diagnostic criteria, if applicable	Page 5
Data sources/ measurement	8*	For each variable of interest, give sources of data and details of methods of assessment (measurement). Describe comparability of assessment methods if there is more than one group	*Page 4-5*
Bias	9	Describe any efforts to address potential sources of bias	Page 4<br>Page 5
Study size	10	Explain how the study size was arrived at	Page 4
Quantitative variables	11	Explain how quantitative variables were handled in the analyses. If applicable, describe which groupings were chosen and why	Page 5
Statistical methods	12	(*a*) Describe all statistical methods, including those used to control for confounding	Page 5
		(*b*) Describe any methods used to examine subgroups	n.a.

For peer review only - http://bmjopen.bmj.com/site/about/guidelines.xhtml

and interactions	
(*c*) Explain how missing data were addressed	No missing data.
(*d*) *Cohort study*—If applicable, explain how loss to follow-up was addressed<br>*Case-control study*—If applicable, explain how matching of cases and controls was addressed<br>*Cross-sectional study*—If applicable, describe analytical methods taking account of sampling strategy	n.a.
(*e*) Describe any sensitivity analyses	n.a.

Continued on next page

For peer review only

**Results**			
Participants	13*	(a) Report numbers of individuals at each stage of study—eg numbers potentially eligible, examined for eligibility, confirmed eligible, included in the study, completing follow-up, and analysed	n.a.
		(b) Give reasons for non-participation at each stage	n.a.
		(c) Consider use of a flow diagram	n.a.
Descriptive data	14*	(a) Give characteristics of study participants (eg demographic, clinical, social) and information on exposures and potential confounders	Page 6
		(b) Indicate number of participants with missing data for each variable of interest	n.a.
		(c) *Cohort study*—Summarise follow-up time (eg, average and total amount)	n.a.
Outcome data	15*	*Cohort study*—Report numbers of outcome events or summary measures over time	n.a.
		Case-control study—Report numbers in each exposure category, or summary measures of exposure	
		Cross-sectional study—Report numbers of outcome events or summary measures	
Main results	16	(*a*) Give unadjusted estimates and, if applicable, confounder-adjusted estimates and their precision (eg, 95% confidence interval). Make clear which confounders were adjusted for and why they were included	Page 8
		(*b*) Report category boundaries when continuous variables were categorized	n.a.
		(*c*) If relevant, consider translating estimates of relative risk into absolute risk for a meaningful time period	n.a.
Other analyses	17	Report other analyses done—eg analyses of subgroups and interactions, and sensitivity analyses	n.a.
**Discussion**			
Key results	18	Summarise key results with reference to study objectives	Page 9
Limitations	19	Discuss limitations of the study, taking into account sources of potential bias or imprecision. Discuss both direction and magnitude of any potential bias	Page 9
Interpretation	20	Give a cautious overall interpretation of results considering objectives, limitations, multiplicity of analyses, results from similar studies, and other relevant evidence	Page 10-11
Generalisability	21	Discuss the generalisability (external validity) of the study results	Page 9
**Other information**			
Funding	22	Give the source of funding and the role of the funders for the present study and, if applicable, for the original study on which the present article is based	Page 11

*Give information separately for cases and controls in case-control studies and, if applicable, for exposed and unexposed groups in cohort and cross-sectional studies.

For peer review only - http://bmjopen.bmj.com/site/about/guidelines.xhtml

Note: An Explanation and Elaboration article discusses each checklist item and gives methodological background and published examples of transparent reporting. The STROBE checklist is best used in conjunction with this article (freely available on the Web sites of PLoS Medicine at http://www.plosmedicine.org/, Annals of Internal Medicine at http://www.annals.org/, and Epidemiology at http://www.epidem.com/). Information on the STROBE Initiative is available at www.strobe-statement.org.

For peer review only

For peer review only - http://bmjopen.bmj.com/site/about/guidelines.xhtml
